# Identifying Vibrations that Control Non-adiabatic Relaxation of Polaritons in Strongly Coupled Molecule–Cavity Systems

**DOI:** 10.1021/acs.jpclett.2c00826

**Published:** 2022-06-30

**Authors:** Ruth H. Tichauer, Dmitry Morozov, Ilia Sokolovskii, J. Jussi Toppari, Gerrit Groenhof

**Affiliations:** † Nanoscience Center and Department of Chemistry, University of Jyväskylä, P.O. Box 35, 40014 Jyväskylä, Finland; ‡ Nanoscience Center and Department of Physics, University of Jyväskylä, P.O. Box 35, 40014 Jyväskylä, Finland

## Abstract

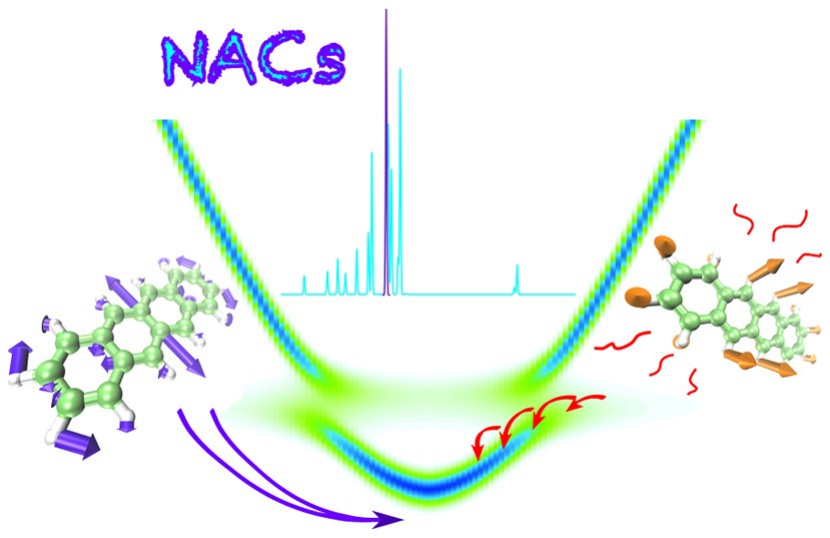

The strong light–matter coupling regime, in which excitations of materials hybridize with excitations of confined light modes into polaritons, holds great promise in various areas of science and technology. A key aspect for all applications of polaritonic chemistry is the relaxation into the lower polaritonic states. Polariton relaxation is speculated to involve two separate processes: vibrationally assisted scattering (VAS) and radiative pumping (RP), but the driving forces underlying these two mechanisms are not fully understood. To provide mechanistic insights, we performed multiscale molecular dynamics simulations of tetracene molecules strongly coupled to the confined light modes of an optical cavity. The results suggest that both mechanisms are driven by the same molecular vibrations that induce relaxation through nonadiabatic coupling between dark states and polaritonic states. Identifying these vibrational modes provides a rationale for enhanced relaxation into the lower polariton when the cavity detuning is resonant with specific vibrational transitions.

Recent observations of low-threshold lasing,^1−4^ of enhanced exciton transport,^5−9^ and of modified reactivity under strong light–matter coupling^10−14^ suggest that the strong coupling regime may have great potential in material science.^15,16^ This regime is reached when the rate of energy exchange between excitations in the material and confined light modes, as exist for example in Fabry–Pérot cavities (Figure 1a) or near plasmonic surfaces, exceeds the intrinsic decay rates of both the material and photonic modes.^17^ Under these conditions, excitations in the material hybridize with the confined light modes to form new light–matter states, called polaritons.^18^

**Figure 1:**
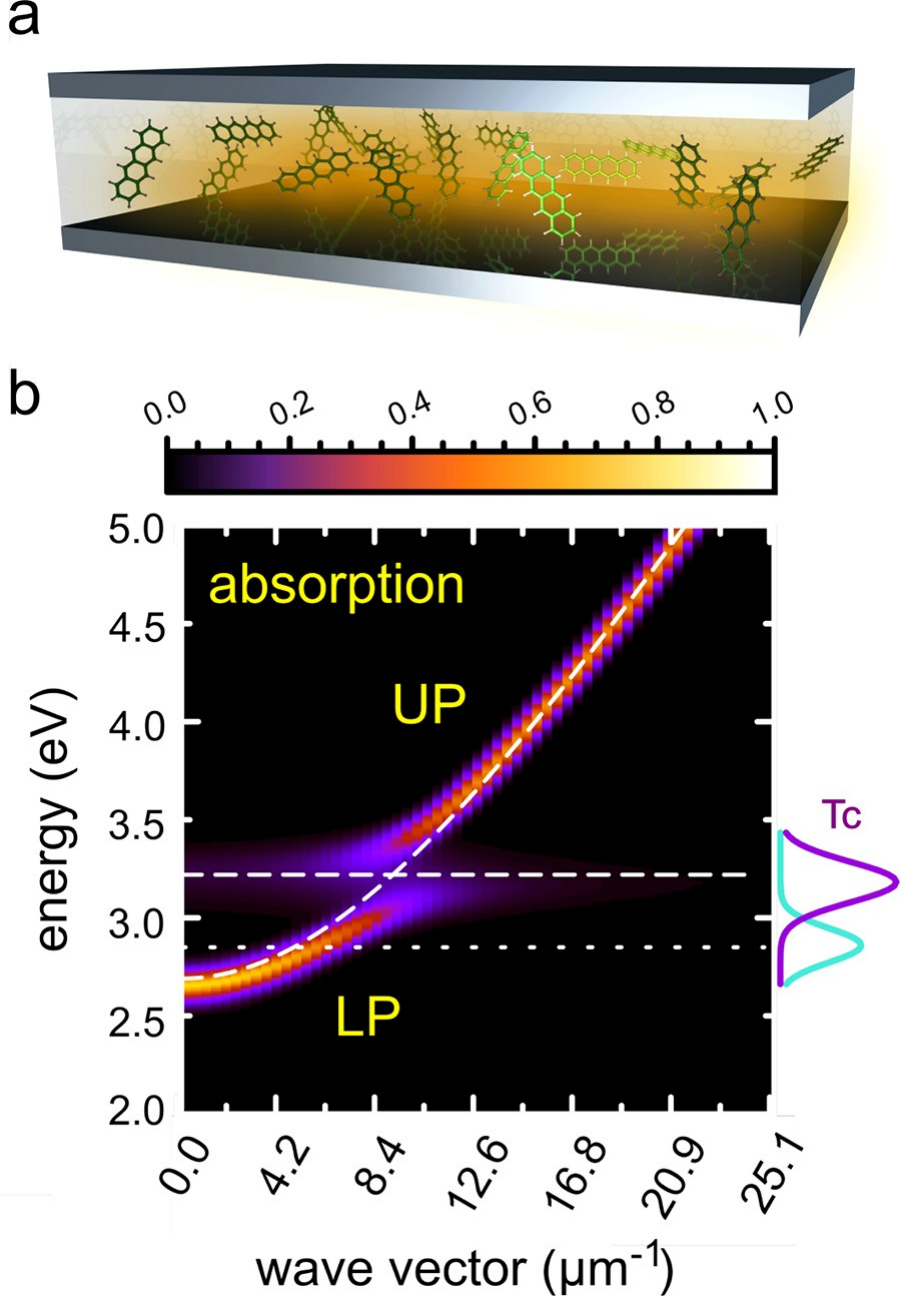
Panel a shows a schematic representation of a Fabry–Pérot cavity filled with tetracene (Tc) molecules (not to scale). Panel b shows the (normalized) absorption as a function of wave vector (*k*_*z*_ = 2*πn*/*L*_cav_) of a one-dimensional cavity of length *L*_cav_ = 15 μm with a vacuum field strength of 0.000176777 au (0.909024 MVcm^–1^) and a cavity resonance at *k* = 0 μm^–1^ of 2.69 eV, filled with 512 Tc molecules. The dispersion of the empty cavity is shown as a dashed white line, as is the excitation energy maximum of Tc at 300 K (3.22 eV, evaluated at the TDA-CAMB3LYP/6-31G(d) level of theory). The absorption (violet) and emission spectra (turquoise) of bare Tc at 300 K are plotted on the right *y* axis. The upper (UP) and lower polariton (LP) branches are indicated.

Within the rotating wave approximation (RWA), valid under weak driving conditions typically employed in strong coupling experiments, the multimode Tavis–Cummings model provides conceptual insight into polariton formation when *N* optically active molecules strongly couple to the *n*_mode_ confined light modes of an optical cavity:^19,20^
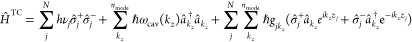
1Here, σ̂_*j*_^+^ (σ̂_*j*_^–^) is the operator that excites (de-excites) the *j*th molecule at position *z*_*j*_ with excitation energy *hν*_*j*_ (horizontal dashed white line in Figure 1b) from the electronic ground (excited) state |S_0_^*j*^⟩ (|S_1_^*j*^⟩) to the electronic excited (ground) state |S_1_^*j*^⟩ (|S_0_^*j*^⟩); *â*_*k_z_*_ (*â*_*k_z_*_^†^) is the photonic annihilation (creation) operator for an excitation of a cavity mode with wave vector *k*_*z*_ and energy ℏω_cav_(*k*_*z*_) (curved dashed line in Figure 1b). The strong light–matter interaction is modeled within the dipolar or long-wavelength approximation through *g*_*jk*_*z*__:^21^
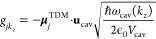
2where **u**_cav_ is a unit vector in the direction of the electric field of the cavity vacuum field, ϵ_0_ is the vacuum permittivity, *V*_cav_ is the cavity mode volume, and **μ**_*j*_^TDM^ is the transition dipole moment of molecule *j*.

The eigenstates of the multimode Tavis–Cummings Hamiltonian are coherent superpositions of excitations in the molecules and of excitations in the cavity modes:
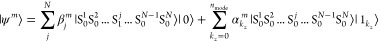
3with eigenenergies *E*_*m*_. The β_*j*_^*m*^ and α_*k*_*z*__^*m*^ expansion coefficients reflect the contribution of the molecular excitations (|S_1_^*j*^⟩) and of the cavity modes (|1_*k_z_*_⟩) to polariton |ψ^*m*^⟩. Due to thermal fluctuations, the excitation energies and transition dipole moments of the molecules span a distribution rather than a single value. Therefore, at thermal equilibrium, the expansion coefficients are all different.

For large, realistic molecule–cavity systems, only few states have a significant contribution from the cavity modes.^22^ These *bright* states can absorb light and because they inherit the dispersion of the cavity modes, they form the wave-vector dependent lower (LP) and upper polariton (UP) branches, as shown in Figure 1b. These branches are separated by the Rabi splitting, defined as the energy gap between the UP and LP at the wave vector where the molecular excitation energy matches the cavity mode energy. In contrast, the large majority of states lack such photonic contribution and are therefore *dark*. Without contributions from the cavity modes, dark states have energies that do not depend on the wave vector and thus lack dispersion.

While entropy favors population of the much more numerous dark states,^23^ altered chemistry, enhanced transport, and lasing are attributed to the LP. Therefore, it is essential to understand how to maximize transfer into LP states. Optical pumping provides a convenient way to populate those states directly via resonant excitation into the LP branch^24^ or indirectly either via resonant excitation into the UP branch^25,26^ or via nonresonant excitation into an uncoupled electronic excited state of a molecule.^27−31^ Alternatively, molecules can also be promoted into an (uncoupled) excited state via electrical injection.^32^ In contrast to direct excitation into the LP, the indirect routes require relaxation from the pumped state into the LP.^33^ Although polariton relaxation has been investigated to a great extent in both experiments^26−31,34−39^ and theory,^22,40−54^ it remains unclear what drives this process or how to reverse-engineer the insights from these works for rational design of new molecule–cavity systems. The latter requires relaxation to be predicted from first-principles with quantum chemistry methods, which is the purpose of the present work.

On the basis of previous theoretical models,^40,41,43,47,50,55^ as well as results from both photoluminescence^29,31,56−58^ and polariton condensation experiments,^1−3^ two main mechanisms have been proposed for driving relaxation from the dark state manifold into the LP branch: vibrationally assisted scattering (VAS) and radiative pumping (RP). In VAS, transitions between dark and bright states are accompanied by discrete changes in the eigenstates of specific molecular vibrations.^29,31,40,41^ Therefore, VAS is manifested by an increased photoluminescence intensity from points on the LP branch with energy gaps to the dark state manifold that match the frequency of these vibrational modes. Although energy gaps at which such enhancements are observed often coincide with molecular Raman frequencies,^26,29,56^ not all Raman-active modes contribute to VAS.^31^ Consequently, it remains difficult to decide in advance which vibrational modes to target through the cavity design in order to control relaxation into the LP. The second mechanism, radiative pumping (RP), is speculated to involve direct exchange of photons between uncoupled molecules and polaritonic states, but the details of this mechanism are even less understood. On the basis of a reasonable agreement between the molecular fluorescence and polaritonic photoluminescence lifetimes,^57,58^ it was proposed that a photon, emitted by an uncoupled molecule is reabsorbed by a polaritonic state within the cavity and from there emitted to the outside.

To acquire atomistic insights into these mechanisms and understand their driving forces, we performed multiscale molecular dynamics (MD) simulations,^54,59^ in which we tracked the relaxation from the dark state manifold into the LP. As model system, we used tetracene (Tc), a member of the acene family of polycyclic aromatic compounds that have been extensively used in strong coupling experiments.^1,60,61^ The electronic ground (S_0_) and excited (S_1_) states of Tc molecules were modeled with density functional theory (DFT),^62^ and time-dependent density functional theory (TDDFT),^63^ within the Tamm–Dancoff approximation (TDA),^64^ respectively, using the CAM-B3LYP functional^65,66^ in combination with the 6-31G(d) basis set.^67^ At this level of theory, the vertical excitation energy of Tc is 3.22 eV in the S_0_ minimum geometry, while the energy gap to the ground state is 2.85 eV in the S_1_ minimum. Using Ehrenfest,^68^ or mean-field, dynamics, we computed classical MD trajectories of *N* Tc molecules with their geometric centers evenly distributed along the *z* axis of the cavity (Figure S1) and with their electronic transition dipole moments aligned to the vacuum field of both single-mode and multimode optical cavities. These trajectories thus evolved on the mean-field potential energy surface provided by the total time-dependent polaritonic wave function, |Ψ(*t*)⟩, which was expanded in the basis of the time-independent eigenfunctions of the Tavis–Cummings Hamiltonian (eq 3) that depend only on the positions of the atoms in the molecules:^52,54^
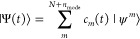
4Here, the *c*_*m*_(*t*) are the time-dependent expansion coefficients that are integrated along with the classical trajectories, using the unitary propagator in the local diabatic basis, which is inherently stable also in the case of (trivial) crossings between potential energy surfaces.^69^ A more detailed description of the methods employed in this work is provided as Supporting Information (SI).

To understand the nature of the dark states, defined here as states with a total contribution of cavity mode excitations below a numerical threshold (i.e., ∑_*k*_*z*__^*n*_mode_^ α_*k*_*z*__^*m*^ < 0.05, eq 3), and, in particular, identify the lowest-energy dark state (DS_0_), we simulated the dynamics after nonresonant excitation into the S_1_ electronic state of one of the *N* molecules (molecule 1). These simulations were performed with *N* = 1, 2, 4, 16, 32, 64, and 128 Tc molecules strongly coupled to a *single* confined light mode with energy ℏω_cav_ = 3.22 eV. Here, we use a single rather than a multimode cavity to control the energy gap between the dark states and the single LP state via the Rabi splitting, defined as
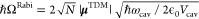
5The Rabi splitting was kept constant at ∼429 meV for all systems by scaling the mode volume of the cavities, *V*_cav_, with the number of molecules, *N*, at the start of the simulation (Table S1).

The results of these simulations, discussed in the SI, suggest that after nonresonant excitation into the S_1_ electronic state of the first molecule, this molecule relaxes into the minimum on its S_1_ potential energy surface, while the other molecules remain in their S_0_ minimum geometries (Figure S3, SI). This finding is in line with results from previous simulations, which also suggest that in the strong coupling regime molecules can still access the S_1_ minimum.^70−73^ We next inspected the eigenstates of the Tavis–Cummings Hamiltonian in this combination of molecular configurations. The contribution of the molecule in the S_1_ minimum energy geometry (molecule 1) to this lowest energy dark state (i.e., |β_1_^DS_0_^|2, eq 3), listed in Table S1, rapidly converges to unity upon increasing the number of molecules. Since in experiment the number of molecules is much larger than we can include in our simulations, these results suggest that in reality one-photon excitations are almost completely localized onto single molecules in the dark state manifold, confirming an earlier assumption by Agranovich and co-workers about the nature of the lowest energy dark state.^40,41^

Before addressing the molecular mechanism of relaxation from the lowest energy dark state (DS_0_) into the LP, we first investigated how the relaxation rate depends on the number of molecules, *N*, that are strongly coupled to the cavity. This information is important, because in our simulations we typically employ a much smaller number of molecules than in experiments. We thus performed simulations with *N* = 16, 32, 64, and 128 Tc molecules strongly coupled to a single confined light mode with the same energy as before (ℏω_cav_ = 3.22 eV). The simulations were started in the lowest energy dark state DS_0_ (i.e., |*c*_DS_0__(0)|^2^ = 1 in eq 4), identified above, in which the excitation is localized onto a single Tc molecule that has relaxed into the S_1_ minimum, far below the cavity frequency and hence no longer that strongly coupled.^40,41^ Initial velocities were randomly selected from a Maxwell–Boltzmann distribution at 300 K. For quantitative comparisons between the different ensemble sizes, we assigned the same initial velocities to the separate molecules in each of the ensembles by setting the seed of the random number generator that determines what velocities are selected for each atom in the molecule, equal to the index of that molecule. These initial conditions can cause inhomogeneous broadening, but only on longer time scales.

In Figure 2a, we plot the time evolution of the population in the LP state for the four ensemble sizes. The total population transferred from the lowest energy dark state DS_0_ into the LP during the first 10 fs of the simulation decreases with increasing numbers of molecules. To understand this trend, we invoke the results from first-order time-dependent perturbation theory applied to a two-level system. Because at the start of the simulation the DS_0_ and LP are separated from all other states by at least ∼395 meV and ∼210 meV, respectively, we consider this a sufficiently valid approximation for the early time evolution of this system. Within this approximation, the population of the LP is given by the Rabi formula:
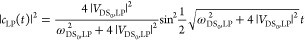
6where *c*_LP_ is the expansion coefficient of the LP state in the total polaritonic wave function (eq 4); ℏω_DS_0_,LP_ = *E*_LP_ – *E*_DS_0__, the energy gap between DS_0_ and the LP; and *V*_DS_0_,LP_, the perturbation term, which is the nonadiabatic coupling between these states. The nonadiabatic coupling, or vibronic coupling, determines the mixing of polaritonic states as a result of molecular vibrations.^74−76^ The nonadiabatic coupling *V*_DS_0_,LP_ depends on the overlap between the nonadiabatic coupling vector **d**_DS_0_,LP_ and the velocities of the atoms **Ṙ**:^77^
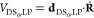
7where **Ṙ** is a vector containing the velocities of all atoms in the system, and the nonadiabatic coupling vector **d**_DS_0_,LP_ is given by
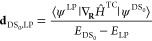
8The direction of this vector determines in what direction population is transferred between the adiabatic states, while its magnitude determines how much population is transferred.

**Figure 2:**
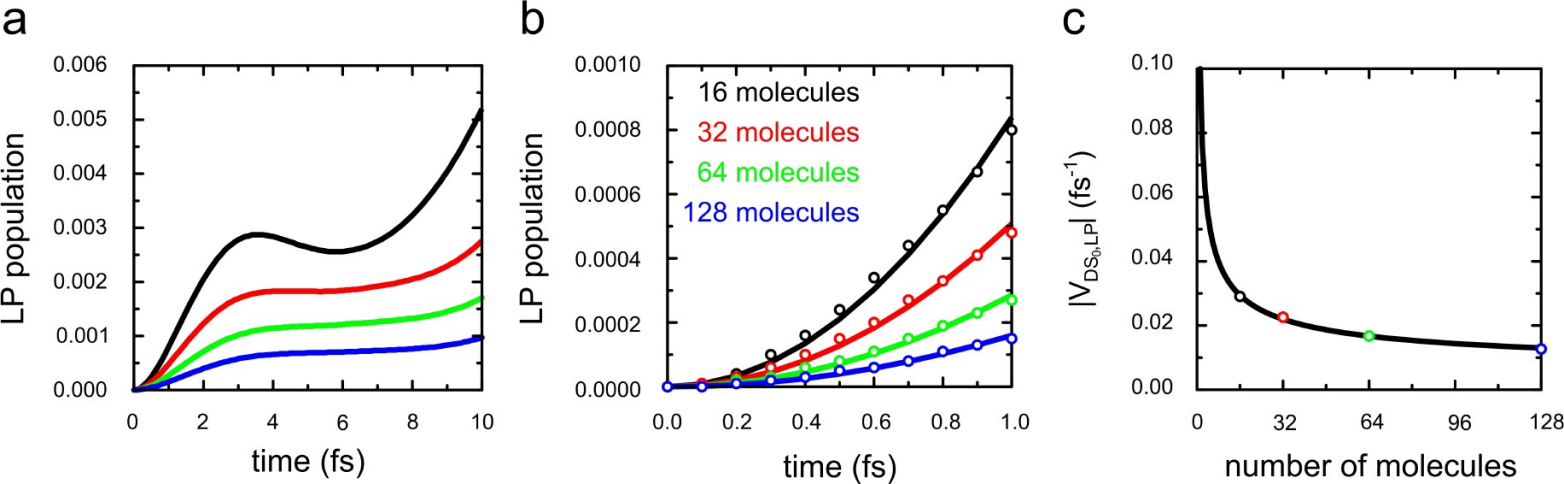
(a) Population of the LP after excitation into the lowest energy dark state (DS_0_) for *N* = 16 (black), 32 (red), 64 (green) and 128 (blue) Tc molecules strongly coupled to a single confined light mode. The vacuum field of the cavity is adjusted to achieve a Rabi splitting of 429 meV. (b) Zoom-in on the population during the first femtosecond. Open circles are the data points from the trajectories, continuous lines fits to the Rabi formula (eq 6). (c) Absolute value of the perturbation term |*V*_DS_0_,LP_|, obtained from the fits in panel b, for the various ensemble sizes of strongly coupled molecules. Open circles are data points; the continuous line is a fit to *a*/√*N* + *b*, with *a* = 0.10 fs^–1^ and *b* = 4 × 10^–3^ fs^–1^.

Because we keep the Rabi splitting constant by scaling the cavity mode volume with the number of molecules, the nonadiabatic coupling vector is inversely proportional to the square root of the number of molecules strongly coupled to the cavity (see SI for a derivation). Indeed, fitting the Rabi formula (eq 6) to the population of the LP during the first femtosecond (Figure 2c) yields estimates for |*V*_DS_0_,LP_| that scale approximately as 1/√*N*. Importantly, because the number of molecules that we can include in our simulations is much smaller than the number of molecules in a typical Fabry–Pérot cavity, we overestimate the rate at which the LP is populated from the dark state manifold as compared to experiment. Conversely, the transfer from the LP into the dark state manifold should be comparable to experiment, as the number of dark states into which the population can transfer (i.e., the density of final states) increases with *N*, canceling the 1/*N* dependence of the |*V*_LP,DS_0__|^2^ perturbation term in eq 6. In principle, therefore, under equilibrium conditions, we would overestimate the population in the LP, which should be taken into account when comparing results between simulations and experiments. Nevertheless, because the rate decreases with increasing *N*,^22,51,78^ we speculate that in real Fabry–Pérot cavities with approximately 10^6^ molecules inside the mode volume,^51,78^ the lifetime of the DS_0_ state will be sufficiently long to reach thermal equilibrium, in particular if the energy gap to the LP states is large. We therefore assume that before relaxation from the DS_0_ state into the LP occurs, all molecules are in their vibrational ground state.

In the SI, we also show that the nonadiabatic coupling vector **d**_DS_0_,LP_ connecting the lowest energy dark state (DS_0_) and the LP is dominated by gradients of the Tavis–Cummings Hamiltonian (eq 1) with respect to atomic displacements of the molecule that is in the S_1_ minimum geometry. Indeed, in simulations in which the atoms of that molecule are kept fixed, there is no population transfer from DS_0_ into the LP, whereas in simulations with the other molecules fixed, transfer is observed (Figure S5, SI).

Because the molecular displacements responsible for nonadiabatic transitions between the polaritonic states are linear combinations of the vibrational modes, we computed these modes within the harmonic approximation by diagonalizing the Hessian and plotted their overlaps with the nonadiabatic coupling vector as a function of the vibrational mode energy (i.e., ℏω_*n*_^vib^ for 1 ≤ *n* ≤ 84) in Figure 3a. To understand which of the vibrational modes can induce population transfer between the DS_0_ and LP, we performed 84 short MD simulations of the 16 molecule cavity system. These simulations were initiated in the lowest energy dark state, DS_0_, in which the first Tc molecule is in the S_1_ minimum geometry, while the other 15 molecules are in the S_0_ geometry. In each simulation, we selectively activated one of the 84 vibrational modes of the molecule in the S_1_ minimum energy geometry by providing initial atomic velocities in the direction of the displacements of that mode (see SI for details). To quantify the extent of population transfer from the DS_0_ state into the more photonic LP state during the simulation, we projected the excitation of the single cavity mode (i.e., ⟨1|⟨S_0_^1^S_0_^2^...S_0_^*N*–1^S_0_^*N*^|, eq 3) onto the total time-dependent polaritonic wave function (|Ψ(*t*)⟩, eq 4). The top panel in Figure 3b shows this photonic weight (i.e., |∑_*m*_∑_*j*_*c*_*m*_α_*j*_^*m*^|^2^) at 1 fs as a function of the vibrational energy of the mode along which the initial velocities were directed. The observation that transfer predominantly occurs if we activate vibrational modes that overlap with the nonadiabatic coupling vector suggests that the relaxation from the dark state manifold into the LP is selectively mediated by these vibrations.

**Figure 3:**
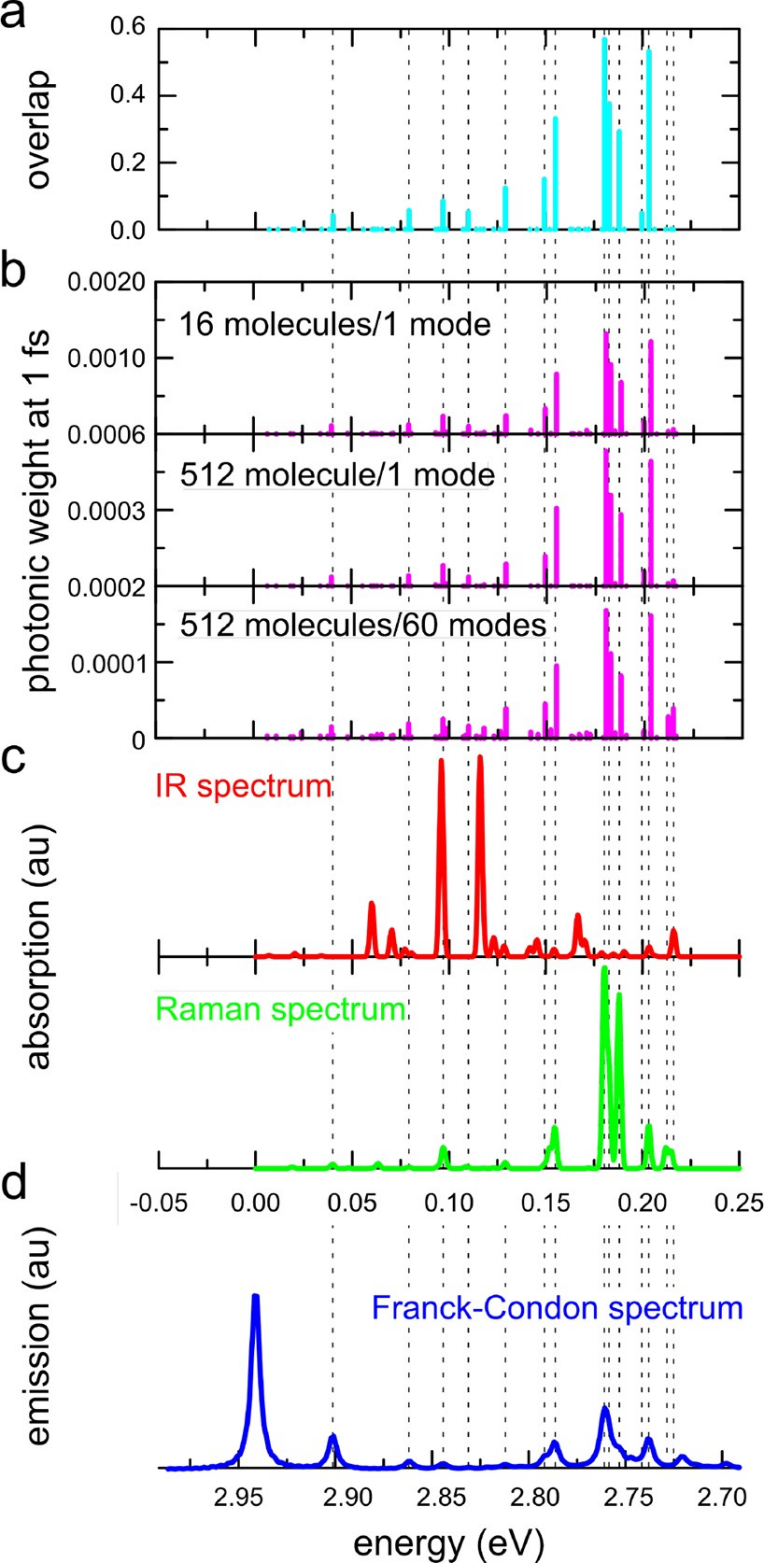
(a) Overlap between the nonadiabatic coupling vector **d**_DS_0_,LP_ (eq 8) and the vibrational modes of Tc, plotted as a function of their energies. (b) Total photonic weight (∑_*m*_∑_*j*_*c*_*m*_α_*j*_^*m*^|^2^) at 1 fs in simulations of 16 and 512 Tc molecules coupled to a single-mode cavity and of 512 Tc molecules coupled to a Fabry–Pérot cavity modeled with 60 modes (Figure 1). The 84 trajectories of these systems were started in the DS_0_ state, with initial atomic velocities along one of the vibrational modes of the Tc molecule in the S_1_ geometry. (c) Calculated infrared (IR) and Raman spectra of Tc. (d) Calculated Franck–Condon fluorescence spectrum, plotted with an inverted *x* axis to match the normal mode energies. The dashed vertical lines are a guide for the eye.

In the 16 molecule system used above, the LP is higher in energy than the DS_0_ (in the cavity of Figure 1, such a situation would correspond to LP states with *k*_*z*_ > 8.4 μm^–1^), whereas in experiments VAS is typically observed when the LP state is lower in energy than the dark state manifold.^29,31^ We therefore repeated the simulations for a system in which the energy of the LP is below the dark state manifold. In this system, we strongly coupled 512 Tc molecules to a single-mode cavity, resonant with the Tc excitation at 3.22 eV and with a vacuum field strength of 0.000493 au (2.5 MVcm^–1^, *V*_cav_ = 144.1 nm^3^). In this system, the lowest energy dark state DS_0_, in which the first Tc molecule is in the S_1_ minimum geometry while the other 511 molecules are in the S_0_ geometry, is 39.1 meV higher in energy than the LP. As before, these simulations were initiated in the DS_0_ state. In each simulation, we again selectively activated one of the 84 vibrational modes of the first Tc molecule, by providing initial atomic velocities in the direction of the displacement of that vibrational mode (see SI for details). The middle panel in Figure 3b shows the contribution of the cavity mode at 1 fs as a function of the vibrational energy of the vibrational mode that was activated. As in the 16 molecule ensemble, enhanced transfer was observed for vibrational modes that overlap with the nonadiabatic coupling vector, confirming that also when the energy of the LP state is below that of the DS_0_ state, relaxation into the LP is mediated by specific vibrations, rather than by all vibrations.

Because Raman spectra have been used to identify vibrational modes that can mediate relaxation,^26,29,31^ we also computed the Raman spectrum. In Figure 3c, we compare the Raman intensities of the vibrational modes to the overlap of these modes with the nonadiabatic coupling vector. Because Tc belongs to the D_2h_ point group, both terms in the nonadiabatic coupling vector are fully symmetric (A_g_, eq 23 in the SI).^72,73^ Therefore, only fully symmetric vibrations of the same A_g_ symmetry can induce population transfer. Since fully symmetric vibrational modes are also Raman-active, these vibrations appear in the Raman spectrum of Figure 3c. However, because in Tc vibrational modes of B_1g_, B_2g_, and B_3g_ symmetry are Raman-active as well, the Raman spectrum also contains peaks due to vibrations that cannot induce population transfer (e.g., vibrational mode 15 of B_1g_ symmetry at 63 meV), in line with experiments.^29,31^

Whereas for Tc the modes that drive relaxation can be identified based on symmetry arguments, for molecules that lack internal symmetry (i.e., C_1_), these modes can only be identified by computing their overlap with the nonadiabatic coupling vector. To illustrate this, we have also performed simulations of a single-mode cavity with 16 rhodamine molecules. The results of these simulations, discussed in the SI and shown in Figure S8, suggest that indeed the Raman spectrum does not predict all of the modes that can drive the relaxation between the DS_0_ and the LP state, whereas these modes are easily identified from their overlap with the nonadiabatic coupling vector.

The nonadiabatic coupling vector contains two terms (eq 20, SI), the first of which is the gradient difference vector (i.e., ∇_**R**_*V*_S_1__(**R**) – ∇_**R**_*V*_S_0__(**R**)). Therefore, as in previous works,^72,73^ the modes that drive the relaxation process could be identified from their overlap with the gradient difference vector as well. Indeed, because the gradient difference vector at the S_0_ or S_1_ minima (eq 23 in the SI) describes the displacement between these minima, all modes that are Franck–Condon active can induce population transfer from the DS_0_ state into the LP. These modes can either be computed or identified from the vibronic progression in an absorption or emission spectrum (Figure 3d for Tc, Figure S8d for rhodamine). In contrast to the Raman and vibronic spectra, the IR spectrum, also shown in Figure 3c, is of limited value for predicting vibrational modes that enhance relaxation into the LP for both Tc and rhodamine.

As shown by Pechukas, during nonadiabatic transitions, a semiclassical trajectory evolves under a force in the direction of the nonadiabatic coupling vector.^79,80^ The work performed by this force dissipates the energy gap, Δ*E*_DS_0_,LP_ = *E*_LP_ – *E*_DS_0__, between the adiabatic states into a displacement.^81^ Whereas in our classical MD simulations, population can transfer at any energy gap due to the absence of energy quantization in the vibrational modes, in reality, transfer will only occur if the work performed by the Pechukas’ force equals the energy of a vibrational transition between eigenstates of the vibrational mode along which that force acts. Therefore, in addition to the overlap with the nonadiabatic coupling vector (eq 8), population transfer also requires that the energy spacing between the eigenstates of the vibrational mode matches the energy gap between the polaritonic states.

Because the latter aspect of molecular vibrations cannot be easily modeled in classical MD simulations, we performed quantum dynamics simulations of a simple model system instead, in which the LP and DS_0_ polaritonic surfaces are described by one-dimensional harmonic potentials coupled through the nonadiabatic coupling. The details of this model are presented in the SI. The parameters for the vibrational frequency, energy gap, and nonadiabatic coupling in this model were derived from the simulation with 512 Tc molecules. As shown in Figure S9, if the energy gap, Δ*E*_DS_0_,LP_, between the DS_0_ and LP states is the same as the vibrational energy spacing, *ℏω*^vib^, population transfers rapidly from the vibrational ground state in DS_0_ (|0⟩_DS_0__) into the vibrational first excited state in the LP (|1⟩_LP_). In contrast, if the gap between the LP and DS_0_ states does not match the energy difference between the vibrational levels, even by as little as a few meV, there is no transfer. On the basis of these results, we speculate that the two requirements for vibrations to mediate population transfer from the dark state manifold into the LP, namely, overlap with the nonadiabatic coupling vector **d**_DS_0_,LP_ and resonance with Δ*E*_DS_0_,LP_, provide a rationale for the luminescence enhancement at specific vibrational frequencies observed experimentally.^26,29,31,56^ While previous theoretical analyses had already included a phenomenological decay channel involving vibrational modes,^31,40,41,47,50^ the added value of our work is that such modes can actually be predicted from first-principles computations of nonadiabatic coupling vectors and vibrational normal modes.

Finally, we investigated the radiative pumping (RP) mechanism, which has been proposed to dominate the relaxation process if the fluorescence spectrum of the molecule overlaps with the LP branch.^26,57,58^ This mechanism is speculated to involve a direct exchange of a photon between an excited molecule and the LP branch. To mimic the initial conditions under which RP is speculated to occur, we modeled a molecule–cavity system with 512 Tc molecules in a periodic one-dimensional cavity (Figure 1b),^42,54^ in which the DS_0_ state at 2.85 eV is nearly degenerate with several states on the LP branch. The cavity was red-detuned with ℏω_0_ = 2.69 eV at *k*_*z*_ = 0 μm^–1^. To increase the number of LP states with energies near the DS_0_ state, the dispersion of this cavity, , was modeled with 60 discrete modes, i.e., *k*_*z*_ = 2π*n*/*L*_cav_ with *L*_cav_ = 15 μm the cavity length and 0 ≤ *n* < 59 the mode index. As before, the 84 short trajectories were initiated with velocities along the displacement vectors of each of the vibrational modes of the Tc molecule in the S_1_ minimum geometry. To reduce the coupling to the cavity and increase localization of the excitation, this molecule was oriented such that the angle between its transition dipole moment and the cavity vacuum field was 80°.

In contrast to the single-mode cavities, there are many states forming the LP branch in this system. Therefore, to quantify population transfer from the DS_0_ state into all of these LP states, we summed the projections of all cavity mode excitations (eq 3) onto the total polaritonic wave function (eq 4), i.e., ∑_*k*_⟨1_*k*_|⟨S_0_^1^S_0_^2^...S_0_^*N*^|Ψ(*t*)⟩. In the third row of Figure 3b, we plot the sum of these projections at 1 fs as a function of the vibrational mode energy. As in the single-mode cavity systems, population transfer occurs most efficiently for displacements of vibrational modes that overlap with the nonadiabatic coupling vector **d**_DS_0_,LP_ (Figure 3a).

Thus, even though we do not explicitly account for the emission or reabsorption of photons in our simulations, we still observe relaxation from the DS_0_ state into the LP branch when these states nearly overlap, as required for RP. We also observe that this process is driven by the same vibrational modes as when there is an energy gap between these states. We therefore suggest that the mechanism previously identified as RP^57,58^ does not involve a direct exchange of a photon but rather should be considered a form of VAS, which does not require a change in the vibrational eigenstates, because there is no gap between the DS_0_ and LP states.

In conclusion, the results of our atomistic MD simulations of tetracene molecules strongly coupled to the confined light modes of a Fabry–Pérot cavity suggest that displacements of specific vibrational modes drive nonadiabatic relaxation from the dark state manifold into the lower polariton. These vibrations can be identified by their overlap with the nonadiabatic coupling vector **d**_DS_0_,LP_ between the lowest-energy dark state DS_0_, in which the excitation is localized onto a single molecule,^40,41^ and the LP, in which the excitation is delocalized over many molecules and cavity modes. The efficiency of this relaxation depends on the energy gap between the lowest energy dark state DS_0_ and the LP, not only because the magnitude of **d**_DS_0_,LP_ is inversely proportional to that gap (eq 8) but also because the frequencies of the vibrational modes need to match that gap for population transfer to occur. Because it is possible to identify these modes with *ab initio* calculations, our work paves the way for systematic optimization of relaxation channels in molecule–cavity systems for specific applications. Finally, we could also show that the nonadiabatic coupling vector **d**_DS_0_,LP_ scales as the inverse of the square root of the number of strongly coupled molecules. Because simulations are typically performed with fewer molecules than in experiments, this scaling allows one to identify finite-size artifacts that must be taken into account when comparing results between simulations and experiments. In the context of our work, the scaling implies that, in the thermodynamic limit with approximately 10^6^ molecules inside the mode volume of a Fabry–Pérot cavity,^51,78^ the nonadiabatic coupling vectors would approximately be 50 times smaller than in our simulations with 512 molecules but still be in line with the (sub)picosecond relaxation times measured experimentally.^25,39,82,83^

## Supporting Information Available

Details on the multiscale molecular dynamics model, simulation details, characterization of the lowest-energy dark state DS_0_, analysis of the nonadiabatic coupling vector, simulations of a rhodamine-cavity system, and details on the model system for vibronic transitions between polaritons (PDF)
Transparent Peer Review report available (PDF)

## References

[ref1] Kéna-Cohen S.; Forrest S. R. Room-temperature polariton lasing in an organic single-crystalmicrocavity. Nat. Photonics 2010, 4, 371–375. 10.1038/nphoton.2010.86.

[ref2] Ramezani M.; Halpin A.; Fernández-Domínguez A. I.; Feist J.; Rodriguez S. R.-K.; Garcia-Vidal F. J.; Gomez Rivas J. Plasmon-exciton-polariton lasing. Optica 2017, 4, 31–37. 10.1364/OPTICA.4.000031.

[ref3] Zasedatelev A. V.; Baranikov A. V.; Sannikov D.; Urbonas D.; Scafirimuto F.; Shishkov V. Y.; Andrianov E. S.; Lozovik Y. E.; Scherf U.; Stöferle T.; Mahrt R. F.; Lagoudakis P. G. Single-photon nonlinearity at room temperature. Nature 2021, 597, 493–497. 10.1038/s41586-021-03866-9. 34552252

[ref4] Akselrod G. M.; Young E. R.; Bradley M. S.; Bulović V. Lasing through a strongly-coupled mode by intra-cavity pumping. Opt. Express 2013, 21, 12122–12128. 10.1364/OE.21.012122. 23736432

[ref5] Lerario G.; Ballarini D.; Fieramosca A.; Cannavale A.; Genco A.; Mangione F.; Gambino S.; Dominici L.; De Giorgi M.; Gigli G.; Sanvitto D. High-speed flow of interacting organic polaritons. Light: Sci. Appl. 2017, 6, e16212 10.1038/lsa.2016.212. 30167229 PMC6062184

[ref6] Rozenman G. G.; Akulov K.; Golombek A.; Schwartz T. Long-Range Transport of Organic Exciton-Polaritons Revealed by Ultrafast Microscopy. ACS Photonics 2018, 5, 105–110. 10.1021/acsphotonics.7b01332.

[ref7] Du M.; Martínez-Martínez L. A.; Ribeiro R. F.; Hu Z.; Menon V. M.; Yuen-Zhou J. Theory for polariton-assisted remote energy transfer. Chem. Sci. 2018, 9, 6659–6669. 10.1039/C8SC00171E. 30310599 PMC6115621

[ref8] Hou S.; Khatoniar M.; Ding K.; Qu Y.; Napolov A.; Menon V. M.; Forrest S. R. Ultralong-Range Energy Transport in a Disordered Organic Semiconductor at Room Temperature Via Coherent Exciton-Polariton Propagation. Adv. Mater. 2020, 32, 2002127 10.1002/adma.202002127. 32484288

[ref9] Georgiou K.; Jayaprakash R.; Othonos A.; Lidzey D. G. Ultralong-Range Polariton-Assisted Energy Transfer in Organic Microcavities. Angew. Chem., Int. Ed. 2021, 60, 16661–16667. 10.1002/anie.202105442. PMC8361947 33908681

[ref10] Hutchison J. A.; Schwartz T.; Genet C.; Devaux E.; Ebbesen T. W. Modifying Chemical Landscapes by Coupling to Vacuum Fields. Angew. Chem., Int. Ed. 2012, 51, 1592–1596. 10.1002/anie.201107033. 22234987

[ref11] Stranius K.; Hertzog M.; Borjesson K. Selective manipulation of electronically excited states through strong light-matter interactions. Nat. Commun. 2018, 9, 2273 10.1038/s41467-018-04736-1. 29891958 PMC5995866

[ref12] Thomas A.; Lethuillier-Karl L.; Nagarajan K.; Vergauwe R. M. A.; George J.; Chervy T.; Shalabney A.; Devaux E.; Genet C.; Moran J.; Ebbesen T. W. Tilting a ground-state reactivity landscape by vibrational strong coupling. Science 2019, 363, 615–619. 10.1126/science.aau7742. 30733414

[ref13] Vergauwe R. M. A.; Thomas A.; Nagarajan K.; Shalabney A.; George J.; Chervy T.; Seidel M.; Devaux E.; Torbeev V.; Ebbesen T. W. Cavity Catalysis by Cooperative Vibrational Strong Coupling of Reactant and Solvent Molecules. Angew. Chem., Int. Ed. 2019, 58, 15324–15328. 10.1002/anie.201908876. PMC6856831 31449707

[ref14] Lather J.; Bhatt P.; Thomas A.; Ebbesen T. W.; George J. Cavity Catalysis by Cooperative Vibrational Strong Coupling of Reactant and Solvent Molecules. Angew. Chem., Int. Ed. 2019, 58, 10635–10638. 10.1002/anie.201905407. PMC6771697 31189028

[ref15] Garcia-Vidal F. J.; Ciuti C.; Ebbesen T. W. Manipulating matter by strong coupling to vacuum fields. Science 2021, 373, eabd0336 10.1126/science.abd0336. 34244383

[ref16] Ribeiro R. F.; Martinez-Martinez L. A.; Du M.; Campos-Gonzalez-Angulo J.; Yuen-Zhou J. Polariton chemistry: controlling molecular dynamics with optical cavities. Chem. Sci. 2018, 9, 6325–6339. 10.1039/C8SC01043A. 30310561 PMC6115696

[ref17] Törmä P.; Barnes W. L. Strong coupling between surface plasmon polaritons and emitters: a review. Rep. Prog. Phys. 2015, 78, 013901 10.1088/0034-4885/78/1/013901. 25536670

[ref18] Feist J.; Galego J.; Garcia-Vidal F. J. Polaritonic chemistry with organic molecules. ACS Photonics 2018, 5, 205–216. 10.1021/acsphotonics.7b00680.

[ref19] Jaynes E. T.; Cummings F. W. Comparison of quantum and semiclassical radiation theories with to the beam maser. Proc. IEEE 1963, 51, 89–109. 10.1109/PROC.1963.1664.

[ref20] Tavis M.; Cummings F. W. Approximate solutions for an N-molecule radiation-field Hamiltonian. Phys. Rev. 1969, 188, 692–695. 10.1103/PhysRev.188.692.

[ref21] Kowalewski M.; Bennett K.; Mukamel S. Non-adiabatic dynamics of molecules in optical cavities. J. Chem. Phys. 2016, 144, 054309 10.1063/1.4941053. 26851923

[ref22] del Pino J.; Feist J.; Garcia-Vidal F. J. Quantum Theory of Collective Strong Coupling of Molecular Vibrations with a Microcavity Mode. New J. Phys. 2015, 17, 053040 10.1088/1367-2630/17/5/053040.

[ref23] Scholes G. D.; DelPo C. A.; Kudisch B. Entropy Reorders Polariton States. J. Phys. Chem. Lett. 2020, 11, 6389–6395. 10.1021/acs.jpclett.0c02000. 32678609

[ref24] Vasista A. B.; Menghrajani K. S.; Barnes W. L. Polariton assisted photoemission from a layered molecular material: role of vibrational states and molecular absorption. Nanoscale 2021, 13, 14497–14505. 10.1039/D1NR03913J. 34473173 PMC8412029

[ref25] Virgili T.; Coles D.; Adawi A. M.; Clark C.; Michetti P.; Rajendran S. K.; Brida D.; Polli D.; Cerullo G.; Lidzey D. G. Ultrafast polariton relaxation dynamics in an organic semiconductor microcavity. Phys. Rev. B 2011, 83, 245309 10.1103/PhysRevB.83.245309.

[ref26] Hulkko E.; Pikker S.; Tiainen V.; Tichauer R. H.; Groenhof G.; Toppari J. J. Effect of molecular Stokes shift on polariton dynamics. J. Chem. Phys. 2021, 154, 154303 10.1063/5.0037896. 33887943

[ref27] Lidzey D.; Fox A.; Rahn M.; Skolnick M.; Agranovich V.; Walker S. Experimental Study of Light Emission from Strongly Coupled Organic Semiconductor Microcavities Following Nonresonant Laser Excitation. Phys. Rev. B 2002, 65, 195312-1–195312-10. 10.1103/PhysRevB.65.195312.

[ref28] Lodden G.; Holmes R. Electrical Excitation of Microcavity Polaritons by Radiative Pumping from a Weakly Coupled Organic Semiconductor. Phys. Rev. B 2010, 82, 125317 10.1103/PhysRevB.82.125317.

[ref29] Coles D. M.; Michetti P.; Clark C.; Tsoi W.; Adawi A. M.; Kim J.; Lidzey D. G. Vibrationally Assisted Polariton-Relaxation Processes in Strongly Coupled Organic-Semiconductor Microcavities. Adv. Funct. Mater. 2011, 21, 3691–3696. 10.1002/adfm.201100756.

[ref30] Coles D. M.; Grant R.; Lidzey D. G.; Clark C.; Lagoudakis P. G. Imaging the polariton relaxation bottleneck in strongly coupled organic semiconductor microcavities. Phys. Rev. B 2013, 88, 121303 10.1103/PhysRevB.88.121303.

[ref31] Somaschi N.; Mouchliadis L.; Coles D.; Perakis I. E.; Lidzey D. G.; Lagoudakis P. G.; Savvidis P. G. Ultrafast polariton population build-up mediated by molecular phonons in organic microcavities. Appl. Phys. Lett. 2011, 99, 143303 10.1063/1.3645633.

[ref32] Tischler J. R.; Bradley M. S.; Bulovic V.; Song J. H.; Nurmikko A. Strong Coupling in a Microcavity LED. Phys. Rev. Lett. 2005, 95, 036401 10.1103/PhysRevLett.95.036401. 16090759

[ref33] Fassioli F.; Park K. H.; Bard S. E.; Scholes G. D. Femtosecond Photophysics of Molecular Polaritons. J. Phys. Chem. Lett. 2021, 12, 11444–11459. 10.1021/acs.jpclett.1c03183. 34792371

[ref34] Lidzey D. G.; Bradley D. D. C.; Skolnick M. S.; Virgili T.; Walker S.; Whittaker D. M. Strong exciton-photon coupling in an organic semiconductor microcavity. Nature 1998, 395, 53–55. 10.1038/25692.

[ref35] Lidzey D.; Virgili T.; Bradley D.; Skolnick M.; Walker S.; Whittaker D. Observation of Strong Exciton-Photon Coupling in Semiconductor Microcavities Containing Organic Dyes and J-aggregates. Opt. Mater. 1999, 12, 243–247. 10.1016/S0925-3467(99)00051-8.

[ref36] Lidzey D. G.; Bradley D. D. C.; Virgili T.; Armitage A.; Skolnick M.; Walker S. Room Temperature Polariton Emission from Strongly Coupled Organic Semiconductor Microcavities. Phys. Rev. Lett. 1999, 82, 3316–3319. 10.1103/PhysRevLett.82.3316.

[ref37] Lidzey D.; Bradley D.; Armitage A.; Walker S.; Skolnick M. Photon-Mediated Hybridization of Frenkel Excitons in Organic Semiconductor Microcavities. Science 2000, 288, 1620–1623. 10.1126/science.288.5471.1620. 10834836

[ref38] Baieva S.; Hakamaa O.; Groenhof G.; Heikkilä T. T.; Toppari J. J. Dynamics of strongly coupled modes between surface plasmon polaritons and photoactive molecules: the effect of the Stokes shift. ACS Photonics 2017, 4, 28–37. 10.1021/acsphotonics.6b00482.

[ref39] Mewes L.; Wang M.; Ingle R. A.; Börjesson K.; Chergui M. Energy relaxation pathways between light-matter states revealed by coherent two-dimensional spectroscopy. Commun. Phys. 2020, 3, 157 10.1038/s42005-020-00424-z.

[ref40] Agranovich V. M.; Litinskaia M.; Lidzey D. G. Cavity polaritons in microcavities containing disordered organic semiconductors. Phys. Rev. B 2003, 67, 085311 10.1103/PhysRevB.67.085311.

[ref41] Litinskaya M.; Reineker P.; Agranovich V. M. Fast polariton relaxation in strongly coupled organic microcavities. J. Lumin. 2004, 110, 364–372. 10.1016/j.jlumin.2004.08.033.

[ref42] Michetti P.; La Rocca G. C. Polariton states in disordered organic microcavities. Phys. Rev. B 2005, 71, 115320 10.1103/PhysRevB.71.115320.

[ref43] Litinskaya M.; Reineker P. Balance between incoming and outgoing cavity polaritons in a disordered organic microcavity. J. Lumin. 2007, 122–123, 418–420. 10.1016/j.jlumin.2006.01.175.

[ref44] Agranovich V.; Gartstein Y. Nature and Dynamics of Low-Energy Exciton Polaritons in Semiconductor Microcavities. Phys. Rev. B 2007, 75, 075302 10.1103/PhysRevB.75.075302.

[ref45] Litinskaya M. Propagation and Localization of Polaritons in Disordered Organic Microcavities. Phys. Lett. A 2008, 372, 3898–3903. 10.1016/j.physleta.2008.02.062.

[ref46] Michetti P.; La Rocca G. C. Simulation of J-aggregate microcavity photoluminescence. Phys. Rev. B 2008, 77, 195301 10.1103/PhysRevB.77.195301.

[ref47] Mazza L.; Fontanesi L.; La Rocca G. C. Organic-based microcavities with vibronic progressions: Photoluminescence. Phys. Rev. B 2009, 80, 235314 10.1103/PhysRevB.80.235314.

[ref48] Michetti P.; La Rocca G. C. Exciton-phonon scattering and photoexcitation dynamics in J-aggregate microcavities. Phys. Rev. B 2009, 79, 35325 10.1103/PhysRevB.79.035325.

[ref49] Michetti P.; La Rocca G. C. Polariton-polariton scattering in organic microcavities at high excitation densities. Phys. Rev. B 2010, 82, 115327 10.1103/PhysRevB.82.115327.

[ref50] Mazza L.; Kena-Cohen S.; Michetti P.; La Rocca G. C. Microscopic theory of polariton lasing via vibronically assisted scattering. Phys. Rev. B 2013, 88, 075321 10.1103/PhysRevB.88.075321.

[ref51] Martínez-Martínez L. A.; Eizner E.; Kéna-Cohen S.; Yuen-Zhou J. Triplet harvesting in the polaritonic regime: A variational polaron approach. J. Chem. Phys. 2019, 151, 054106 10.1063/1.5100192.

[ref52] Groenhof G.; Climent C.; Feist J.; Morozov D.; Toppari J. J. Tracking Polariton Relaxation with Multiscale Molecular Dynamics Simulations. J. Chem. Phys. Lett. 2019, 10, 5476–5483. 10.1021/acs.jpclett.9b02192. PMC6914212 31453696

[ref53] Arnardottir K. B.; Moilanen A. J.; Strashko A.; Törmä P.; Keeling J. Multimode Organic Polariton Lasing. Phys. Rev. Lett. 2020, 125, 233603 10.1103/PhysRevLett.125.233603. 33337197

[ref54] Tichauer R. H.; Feist J.; Groenhof G. Multiscale simulations of molecular polaritons: the effect of multiple cavity modes on polariton relaxation. J. Chem. Phys. 2021, 154, 104112 10.1063/5.0037868. 33722041

[ref55] Chovan J.; Perakis I. E.; Ceccarelli S.; Lidzey D. G. Controlling the interactions between polaritons and molecular vibrations in strongly coupled organic semiconductor microcavities. Phys. Rev. B 2008, 78, 045320 10.1103/PhysRevB.78.045320.

[ref56] Ballarini D.; De Giorgi M.; Gambino S.; Lerario G.; Mazzeo M.; Genco A.; Accorsi G.; Giansante C.; Colella S.; D'Agostino S.; Cazzato P.; Sanvitto D.; Gigli G. Polariton-Induced Enhanced Emission from an Organic Dye under the Strong Coupling Regime. Adv. Optical Mater. 2014, 2, 1076–1081. 10.1002/adom.201400226.

[ref57] Grant R. T.; Michetti P.; Musser A. J.; Gregoire P.; Virgili T.; Vella E.; Cavazzini M.; Georgiou K.; Galeotti F.; Clark C.; Clark J.; Silva C.; Lidzey D. G. Efficient Radiative Pumping of Polaritons in a Strongly Coupled Microcavity by a Fluorescent Molecular Dye. Adv. Optical Mater. 2016, 4, 1615–1623. 10.1002/adom.201600337.

[ref58] Lüttgens J. M.; Berger F. J.; Zaumseil J. Population of Exciton-Polaritons via Luminescent sp^3^ Defects in Single-Walled Carbon Nanotubes. ACS Photonics 2021, 8, 182–193. 10.1021/acsphotonics.0c01129. 33506074 PMC7821305

[ref59] Luk H.-L.; Feist J.; Toppari J. J.; Groenhof G. Multiscale Molecular Dynamics Simulations of Polaritonic Chemistry. J. Chem. Theory Comput. 2017, 13, 4324–4335. 10.1021/acs.jctc.7b00388. 28749690

[ref60] Berghuis A. M.; Halpin A.; Le-Van Q.; Ramezani M.; Wang S.; Murai S.; Gomez Rivas J. Enhanced Delayed Fluorescence in Tetracene Crystals by Strong Light-Matter Coupling. Adv. Funct. Mater. 2019, 29, 1901317–1901328. 10.1002/adfm.201901317.

[ref61] Berghuis A. M.; Serpenti V.; Ramezani M.; Wang S.; Gomez Rivas J. Light–matter coupling strength controlled by the orientation of organic crystals in plasmonic cavities. J. Chem. Phys. C 2020, 124, 12030–12038. 10.1021/acs.jpcc.0c00692.

[ref62] Hohenberg P.; Kohn W. Imhomogeneous Electron Gas. Phys. Rev. 1964, 136, B864–B871. 10.1103/PhysRev.136.B864.

[ref63] Runge E.; Gross E. K. U. Density-Functional Theory for Time-Dependent Systems. Phys. Rev. Lett. 1984, 52, 997–1000. 10.1103/PhysRevLett.52.997.

[ref64] Hirata S.; Head-Gordon M. Time-dependent density functional theory within the Tamm–Dancoff approximation. Chem. Phys. Lett. 1999, 314, 291–299. 10.1016/S0009-2614(99)01149-5.

[ref65] Becke A. D. A new mixing of Hartree-Fock and local density-functional theories. J. Chem. Phys. 1993, 98, 1372 10.1063/1.464304.

[ref66] Yanai T.; Tew D. P.; Handy N. C. A new hybrid exchange-correlation functional using the Coulomb-attenuating method (CAM-B3LYP). Chem. Phys. Lett. 2004, 393, 51–57. 10.1016/j.cplett.2004.06.011.

[ref67] Ditchfield R.; Hehre W. J.; Pople J. A. Self-Consistent Molecular-Orbital Methods. IX. An Extended Gaussian-Type Basis for Molecular-Orbital Studies of Organic Molecules. J. Chem. Phys. 1971, 54, 724–728. 10.1063/1.1674902.

[ref68] Ehrenfest P. Bemerkung über die angenäherte Gültigkeit der klassischen Mechanik innerhalb der Quantenmechanik. Z. Phys. 1927, 45, 455–457. 10.1007/BF01329203.

[ref69] Granucci G.; Persico M.; Toniolo A. Direct semiclassical simulation of photochemical processes with semiempirical wave functions. J. Chem. Phys. 2001, 114, 10608–10615. 10.1063/1.1376633.

[ref70] Groenhof G.; Toppari J. J. Coherent Light Harvesting through Strong Coupling to Confined Light. J. Phys. Chem. Lett. 2018, 9, 4848–4851. 10.1021/acs.jpclett.8b02032. 30085671 PMC6129961

[ref71] Vendrell O. Coherent dynamics in cavity femtochemistry: Application of the multi-configuration time-dependent Hartree method. Chem. Phys. 2018, 509, 55–65. 10.1016/j.chemphys.2018.02.008.

[ref72] Vendrell O. Collective Jahn-Teller Interactions through Light-Matter Coupling in a Cavity. Phys. Rev. Lett. 2018, 121, 253001 10.1103/PhysRevLett.121.253001. 30608830

[ref73] Ulusoy I. S.; Gomez J. A.; Vendrell O. Modifying the Nonradiative Decay Dynamics through Conical Intersections via Collective Coupling to a Cavity Mode. J. Phys. Chem. A 2019, 123, 8832–8844. 10.1021/acs.jpca.9b07404. 31536346

[ref74] Yarkony D. R. Nonadiabatic Quantum Chemistry—Past, Present, and Future. Chem. Rev. 2012, 112, 481–498. 10.1021/cr2001299. 22050109

[ref75] Worth G. A.; Cederbaum L. A. Beyond Born-Oppenheimer: Molecular Dynamics Through a Conical Intersection. Annu. Rev. Phys. Chem. 2004, 55, 127–158. 10.1146/annurev.physchem.55.091602.094335. 15117250

[ref76] Azumi T.; Matsuzaki K. What does the term ”vibronic coupling” mean?. Photochem. Photobiol. 1977, 25, 315–326. 10.1111/j.1751-1097.1977.tb06918.x.

[ref77] Crespo-Otero R.; Barbatti M. Recent Advances and Perspectives on Nonadiabatic Mixed Quantum-Classical Dynamics. Chem. Rev. 2018, 118, 7026–7068. 10.1021/acs.chemrev.7b00577. 29767966

[ref78] Eizner E.; Martínez-Martínez L. A.; Yuen-Shou J.; Kéna-Cohen S. Inverting Singlet and Triplet Excited States using Strong Light-Matter Coupling. Arxiv 2016, 1–29. 10.1126/sciadv.aax4482 PMC6897552 31840063

[ref79] Pechukas P. Time-dependent semiclassical scattering theory. I. Potential scattering. Phys. Rev. 1969, 181, 166–174. 10.1103/PhysRev.181.166.

[ref80] Pechukas P. Time-dependent semiclassical scattering theory. II. Atomic collisions. Phys. Rev. 1969, 181, 174–185. 10.1103/PhysRev.181.174.

[ref81] Coker D.; Xiao L. Methods for molecular dynamics with nonadiabatic transitions. J. Chem. Phys. 1995, 102, 496–510. 10.1063/1.469428.

[ref82] Schwartz T.; Hutchison J. A.; Leonard J.; Genet C.; Haacke S.; Ebbesen T. W. Polariton Dynamics under Strong Light-Molecule Coupling. ChemPhysChem 2013, 14, 125–131. 10.1002/cphc.201200734. 23233286

[ref83] Delpo C.; Kudisch B.; Park K.; Khan S.; Fassioli F.; Fausti D.; Rand B.; Scholes G. Polariton Transitions in Femtosecond Transient Absorption Studies of Ultrastrong Light-Molecule Coupling. J. Phys. Chem. Lett. 2020, 11, 2667–2674. 10.1021/acs.jpclett.0c00247. 32186878 PMC8154840

