# Supplementary material for: Identifying Vibrations that Control Non-adiabatic Relaxation of Polaritons in Strongly Coupled Molecule–Cavity Systems
Source: J Phys Chem Lett. 2022 Jun 30;13(27):6259–67. doi: 10.1021/acs.jpclett.2c00826 (PMC9289944; doi:10.1021/acs.jpclett.2c00826)
Supplement: Supplementary file 1 — jz2c00826_si_001.pdf [file jz2c00826_si_001.pdf]

Supporting Information
for
Identifying Vibrations that Control Non-Adiabatic Relaxation of Polaritons In Strongly Coupled Molecule-Cavity Systems

Ruth H. Tichauer,[†] Dmitry Morozov,[†] Ilia Sokolovskii,[†] J. Jussi toppari,[‡] and Gerrit Groenhof[*,†]

† *Nanoscience Center and Department of Chemistry, University of Jyväskylä, P.O. Box 35, 40014 Jyväskylä, Finland.*

‡ *Nanoscience Center and Department of Physics, University of Jyväskylä, P.O. Box 35, 40014 Jyväskylä, Finland.*

E-mail: gerrit.x.groenhof@jyu.fi

Contents

1 Multi-scale molecular dynamics simulation model **3**

1.1 Extension of the Tavis-Cummings Hamiltonian to molecular systems 3

1.2 One-dimensional periodic cavity 5

1.3 Semi-classical molecular dynamics 7

2 Simulation Details **8**

2.1 Tetracene model 8

2.2 Molecular dynamics of Tetracene-cavity systems 8

2.3 Rhodamine model 9

2.4 Molecular dynamics of Rhodamine-cavity systems 9

2.5 Simulation Analysis 10

2.6 Infra-Red, Raman and Franck-Condon spectra simulation 11

3 Characterization of the lowest-energy dark state **12**

4 Analysis of non-adiabatic coupling vector **15**

5 Relaxation in a strongly coupled Rhodamine-cavity system **21**

6 Model system for vibronic transitions between polaritons **22**

References **25**

1 Multi-scale molecular dynamics simulation model

1.1 Extension of the Tavis-Cummings Hamiltonian to molecular systems

The multi-scale Molecular Dynamics (MD) method for simulating molecules under strong light-matter coupling was presented previously.[1–3] For completeness, we provide a concise overview here. The method is based on the Tavis-Cummings Hamiltonian of quantum optics, and models the interaction between N molecules and n_{mode} confined light modes in a one-dimensional Fabry-Pérot cavity (Figure S1):[3–6]

$$\begin{aligned}\hat{H}^{\text{TC}} \;=\; & \sum_j^N h\nu_j(\mathbf{R}_j)\hat{\sigma}_j^+\hat{\sigma}_j^- + \sum_{k_z}^{n_{\text{mode}}} \hbar\omega_{\text{cav}}(k_z)\hat{a}_{k_z}^\dagger \hat{a}_{k_z} + \\ & \sum_j^N \sum_{k_z}^{n_{\text{mode}}} \hbar g_j(k_z)\left(\hat{\sigma}_j^+\hat{a}_{k_z}\mathbf{f}_z(z_j) + \hat{\sigma}_j^-\hat{a}_{k_z}^\dagger\mathbf{f}_z^*(z_j)\right) + \\ & \sum_i^N V_{\text{S}_0}(\mathbf{R}_i)\end{aligned} \tag{1}$$

Here, $\hat{\sigma}_j^+$ ($\hat{\sigma}_j^-$) is the operator that excites (de-excites) molecule j from the electronic ground (excited) state $|\text{S}_0^j(\mathbf{R}_j)\rangle$ ($|\text{S}_1^j(\mathbf{R}_j)\rangle$) to the electronic excited (ground) state $|\text{S}_1^j(\mathbf{R}_j)\rangle$ ($|\text{S}_0^j(\mathbf{R}_j)\rangle$); $\mathbf{R}_j$ is the vector of the Cartesian coordinates of all atoms in molecule j, centered at z_j; $\hat{a}_{k_z}$ ($\hat{a}_{k_z}^\dagger$) is the annihilation (creation) operator of an excitation of a cavity mode mode with wave-vector k_z; $\mathbf{f}_z(z_j) = e^{ik_z z_j}$ is the function describing the form of the quantized electromagnetic (EM) field modes, here taken to be that of plane waves with in-plane momentum k_z; $h\nu_j(\mathbf{R}_j)$ is the excitation energy of molecule j, defined as:

$$h\nu_j(\mathbf{R}_j) = V_{\text{S}_1}^{\text{mol}}(\mathbf{R}_j) - V_{\text{S}_0}^{\text{mol}}(\mathbf{R}_j) \tag{2}$$

with $V_{\text{S}_0}^{\text{mol}}(\mathbf{R}_j)$ and $V_{\text{S}_1}^{\text{mol}}(\mathbf{R}_j)$ the adiabatic potential energy surfaces of molecule j in the electronic ground (S_0) and excited (S_1) state, respectively. The last term in Equation 1 is the total potential energy of the system in the absolute ground state (*i.e.* with no excitations in neither the molecules

nor the cavity modes), defined as the sum of the ground-state potential energies of all molecules in the cavity.

The $V_{S_0}^{mol}(\mathbf{R}_j)$ and $V_{S_1}^{mol}(\mathbf{R}_j)$ adiabatic potential energy surfaces can be modeled with *ab initio*, density functional theory (DFT), or hybrid Quantum Mechanics / Molecular Mechanics (QM/MM) methods. While these methods provide access to both ground and excited state potential energy surfaces as well as transition dipole moments for complex systems with many molecular degrees of freedom, the accuracy depends on the level of theory in combination with the size of the atomic basis set. High accuracy results therefore require very large computational efforts. In practice, a trade-off is sought between accuracy and computational efficiency, which often renders the results of calculations qualitative, rather than quantitative. Furthermore, because the high dimensionality of the potential energy surfaces precludes a quantum mechanical description of the nuclear degrees of freedom, classical mechanics is used instead. Therefore, nuclear vibrations are not quantized and population transfers between the adiabatic states have to be modelled in an *ad hoc* manner with surface hopping,[7] or Ehrenfest dynamics.[8] In addition, the sampling of all relevant configurations may require more computational resources (and time) than is available. Nevertheless, the main advantage of atomistic models is that despite their limited accuracy, large and complex systems can be modeled directly, and provide *qualitative* insights into the effect of the chemical structure on the dynamics and energetics of a process.

The third term in Equation 1 describes the light-matter interaction within the dipolar approximation through $g_j(k_z)$:

$$g_j(k_z) = -\boldsymbol{\mu}_j(\mathbf{R}_j) \cdot \mathbf{u}_{\text{cav}} \sqrt{\frac{\hbar\omega_{\text{cav}}(k_z)}{2\epsilon_0 V_{\text{cav}}}} \tag{3}$$

where $\mathbf{u}_{\text{cav}}$ is the unit vector indicating the direction of the electric component of cavity vacuum field, here along the y-direction (Figure S1); ϵ_0 the vacuum permittivity; and V_{cav} the volume into which the mode with in-plane moment k_z is confined.

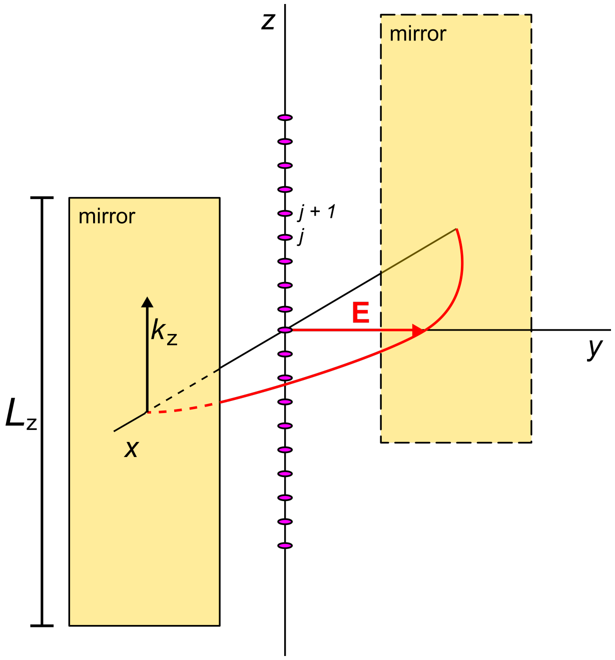

Figure S1: One-dimensional (1D) Fabry-Pérot cavity model. Two reflecting mirrors are located at $-\frac{1}{2}x$ and $\frac{1}{2}x$, confining light modes along this direction, while free propagation along the z direction is possible for plane waves with in-plane momentum k_z and energy $\hbar\omega(k_z)$. The vacuum field vector (red) points along the y-axis, reaching a maximum amplitude at $x = 0$ where the N molecules (magenta ellipses) are placed, distributed along the z-axis at positions z_j with $1 \leq j \leq N$.

1.2 One-dimensional periodic cavity

As in Michetti and La Rocca,[6] we impose periodic boundary conditions in the z-direction, and thus restrict the wave vectors, k_z, to discrete values: $k_z = 2\pi n/L_z$ with $n \in \mathbb{Z}$ and L_z the length of the 1D cavity. With these approximations the molecular Tavis-Cummings Hamiltonian in Equation 1 can be represented as a $(N + n_{\text{mode}})$ by $(N + n_{\text{mode}})$ matrix with four blocks:

$$\mathbf{H}^{\text{TC}} = \begin{pmatrix} \mathbf{H}^{\text{mol}} & \mathbf{H}^{\text{int}} \\ \mathbf{H}^{\text{int}\dagger} & \mathbf{H}^{\text{cav}} \end{pmatrix} \tag{4}$$

The upper left block, $\mathbf{H}^{\text{mol}}$, is a $N \times N$ matrix containing the single-photon excitations of the molecules. Because we neglect direct excitonic interactions between the molecules this block is

diagonal, with elements labeled by the molecule indices j:

$$H^{\text{mol}}_{j,j} = \langle 0|\langle \text{S}^1_0(\mathbf{R}_1)\text{S}^2_0(\mathbf{R}_2)..\text{S}^j_1(\mathbf{R}_j)..\text{S}^N_0(\mathbf{R}_N)|\hat{H}^{\text{TC}}|\text{S}^1_0(\mathbf{R}_1)\text{S}^2_0(\mathbf{R}_2)..\text{S}^j_1(\mathbf{R}_j)..\text{S}^N_0(\mathbf{R}_N)\rangle|0\rangle \quad (5)$$

for $1 \leq j \leq N$. Each matrix element of $\mathbf{H}^{\text{mol}}$ thus represents the potential energy of a molecule (j) in the electronic excited state $|\text{S}^j_1(\mathbf{R}_j)\rangle$ while all other molecules, $i \neq j$, are in the electronic ground state $|\text{S}^i_0(\mathbf{R}_i)\rangle$:

$$H^{\text{mol}}_{j,j} = V^{\text{mol}}_{\text{S}_1}(\mathbf{R}_j) + \sum_{i\neq j}^{N} V^{\text{mol}}_{\text{S}_0}(\mathbf{R}_i) \quad (6)$$

The $|0\rangle$ in Equation 5 indicates that the single-photon Fock states of all cavity modes are empty.

The lower right block, $\mathbf{H}^{\text{cav}}$, is a $n_{\text{mode}} \times n_{\text{mode}}$ matrix (with $n_{\text{mode}} = n_{\text{max}} - n_{\text{min}} + 1$) containing the single-photon excitations of the cavity modes, and is also diagonal:

$$H^{\text{cav}}_{b,b} = \langle 1_b|\langle \text{S}^1_0(\mathbf{R}_1)\text{S}^2_0(\mathbf{R}_2)..\text{S}^N_0(\mathbf{R}_N)|\hat{H}^{\text{TC}}|\text{S}^1_0(\mathbf{R}_1)\text{S}^2_0(\mathbf{R}_2)..\text{S}^N_0(\mathbf{R}_N)\rangle|1_b\rangle \quad (7)$$

for $n_{\text{min}} \leq b \leq n_{\text{max}}$. Here $|1_b\rangle$ is the single-photon Fock state of cavity mode b with wave-vector $k_z = 2\pi b/L_z$. In these matrix elements, all molecules are in the electronic ground state (S_0), while cavity mode b is excited. The energy is therefore the sum of the cavity energy at k_z, and the molecular ground state energies:

$$H^{\text{cav}}_{b,b} = \hbar\omega_{\text{cav}}(2\pi b/L_z) + \sum_{j}^{N} V^{\text{mol}}_{\text{S}_0}(\mathbf{R}_j) \quad (8)$$

Here $\hbar\omega_{\text{cav}}(k_z)$ is the cavity dispersion (dashed curve in Figure 1b, main text):

$$\hbar\omega_{\text{cav}}(k_z) = \sqrt{\hbar^2\omega_0^2 + \hbar^2c^2k_z^2} \quad (9)$$

where $\hbar\omega_0$ is energy at $k_z = 0$, and c the speed of light.

The two $N \times n_{\text{mode}}$ off-diagonal blocks $\mathbf{H}^{\text{int}}$ and $\mathbf{H}^{\text{int}\dagger}$ in the multi-mode Tavis-Cummings Hamiltonian (Equation 4) model the interactions between the molecules and the cavity modes.

These matrix elements are approximated as the overlap between the transition dipole moment of molecule j and the electric field of the cavity mode b at the center z_j of that molecule:

$$H_{j,b}^{\text{int}} = -\boldsymbol{\mu}_j^{\text{TDM}} \cdot \mathbf{u}_{\text{cav}} \sqrt{\frac{\hbar\omega_{\text{cav}}(k_z)}{2\epsilon_0 V_{\text{cav}}}} \langle 0_b | \langle \mathrm{S}_1^j | \hat{\sigma}_j^+ \hat{a}_b e^{i2\pi b z_j / L_z} | \mathrm{S}_0^j \rangle | 1_b \rangle \tag{10}$$

for $1 \leq j \leq N$ and $n_{\text{min}} \leq b \leq n_{\text{max}}$.

1.3 Semi-classical molecular dynamics

The multiple cavity-mode Tavis-Cummings Hamiltonian (Equation 4) is diagonalized at each time step of the MD simulation to obtain $N + n_{\text{mode}}$ (adiabatic) polaritonic eigenstates ψ^m and energies E^m:

$$\psi^m = \sum_j^N \beta_j^m |\mathrm{S}_0^1 \mathrm{S}_0^2 .. \mathrm{S}_1^j .. \mathrm{S}_0^{N-1} \mathrm{S}_0^N \rangle |0\rangle + \sum_{b=n_{\text{min}}}^{n_{\text{max}}} \alpha_b^m |\mathrm{S}_0^1 \mathrm{S}_0^2 .. \mathrm{S}_0^j .. \mathrm{S}_0^{N-1} \mathrm{S}_0^N \rangle |1_b\rangle \tag{11}$$

Because the energies of the electronic ground and excited state, as well as the transition dipole moments of the molecules are functions of the positions of their atoms, the basis functions in which the adiabatic polaritonic eigenstates ψ^m are expanded, depend on the coordinates of all atoms in all molecules, and this dependency carries over to the polaritonic states. Semi-classical MD trajectories of all molecules are computed by numerically integrating Newton's equations of motion. The total polaritonic wave function $\Psi(t)$ is coherently propagated along with the classical degrees of freedom of the N molecules as a time-dependent superposition of the $N + n_{\text{mode}}$ time-independent adiabatic polaritonic states:

$$\Psi(t) = \sum_m^{N+n_{\text{mode}}} c_m(t) \psi^m \tag{12}$$

where $c_m(t)$ are the time-dependent expansion coefficients of the time-independent polaritonic basis functions ψ^m defined in Equation 11. A unitary propagator in the *local* diabatic basis is used to integrate these coefficients,[2,9] while the nuclear degrees of freedom of the N molecules evolve on

the mean-field potential energy surface:

$$V(\mathbf{R}) = \langle \Psi(t)|\hat{H}^{\mathrm{TC}}|\Psi(t)\rangle \tag{13}$$

Because the simulations in this work were run for only a few femtoseconds, cavity decay was neglected. Including decay into this model is, however, possible, as we have shown in previous works.[2,3]

2 Simulation Details

2.1 Tetracene model

Prior to the MD simulations, the ground-state (S_0) geometry of the tetracene molecule was optimized at the CAM-B3LYP/6-31G(d) level of density functional theory (DFT),[10–13] while the excited state (S_1) geometry of tetracene was optimized at the TDA-CAM-B3LYP/6-31G(d) level.[14] A 10 ps MD simulation was performed for an isolated tetracene in the ground state at 300 K, using a stochastic thermostat with a relaxation time of 0.1 ps.[15] The equilibration simulation was used to compute the absorption spectrum shown in Figure 1b of the main text (see below).

2.2 Molecular dynamics of Tetracene-cavity systems

For the simulations in the single-mode cavities, the cavity mode was resonant with the tetracene excitation energy, which is 3.22 eV at the TDA-CAM-B3LYP/6-31G(d) level of theory. For simulations in the multi-mode cavities, the cavity was red-detuned, with a cavity energy of $\hbar\omega_0 = 2.69$ eV at $k_z = 0$ and cavity length of $L_z = 15$ μm. The dispersion of this cavity was modelled with 60 modes ($n_{\text{max}} = 59$), corresponding to an energy cut-off at 5.84 eV, which is sufficiently high above the S_1 energy of tetracene. Unless stated otherwise, the molecules were oriented to maximize the coupling strength by aligning their transition dipole moments to the polarization of the vacuum field inside the cavity at the start of the simulations (*i.e.*, $\mathbf{u}_y$ in Equation 10, Figure S1). In the

multi-mode simulations, the molecules were distributed evenly along the z-axis of the cavity.

Non-resonant excitation of a molecule was achieved by taking the linear combination of the eigenstates (Equation 11) of the Tavis-Cummings Hamiltonian that localizes the excitation on the first molecule, *i.e.*, $\langle 0|\langle S_1^1 S_0^2 ... S_0^N|\Psi(0)\rangle = 1$. Excitation into the DS_0 state was achieved by exciting directly into that state with a hypothetical $\delta(t, E)$ pulse. The integration time step in the classical molecular dynamics simulations was 0.1 fs. Selective activation of a vibrational mode was achieved by providing as initial atomic velocities the mass-unweighted normal mode vector, which was obtained by diagonalizing the molecular Hessian. This vector was multiplied by a factor 5, such that the initial temperature was around 300 K in all simulations. All cavity simulations were performed with Gromacs 4.5.3,[16] in which the multi-mode Tavis-Cummings model presented above, was implemented (available for download from GitHub at https://github.com/rhti/gromacs_qed),[3] using the QM/MM interface to TeraChem[17,18] for ensembles with up until 128 molecules, and to Gaussian16[19] for ensembles with over 128 molecules.

2.3 Rhodamine model

Prior to the MD simulations, we used density functional theory (DFT),[10–13] to optimise the ground-state (S_0) geometry of Rhodamine (Rh), shown in FigureS2, with the CAM-B3LYP functional[11,12] and the 6-31G(d) basis set.[13] The excited state (S_1) was optimized using time-dependent density functional theory (TDDFT),[20] within the Tamm-Dancoff approximation (TDA).[14] At this level of theory, the vertical excitation energy of Rh is 3.49 eV in the S_0 minimum geometry, while the energy gap to the ground state is 3.40 eV in the S_1 minimum.

2.4 Molecular dynamics of Rhodamine-cavity systems

Using Ehrenfest,[8] or mean-field, dynamics, we computed classical MD trajectories of 16 Rh molecules strongly coupled to a single-mode cavity, resonant with the Rh excitation at 3.49 eV and with a vacuum field strength of 0.0006 a.u. The molecules were oriented to maximize the coupling strength by aligning their transition dipole moments to the polarization of the vacuum

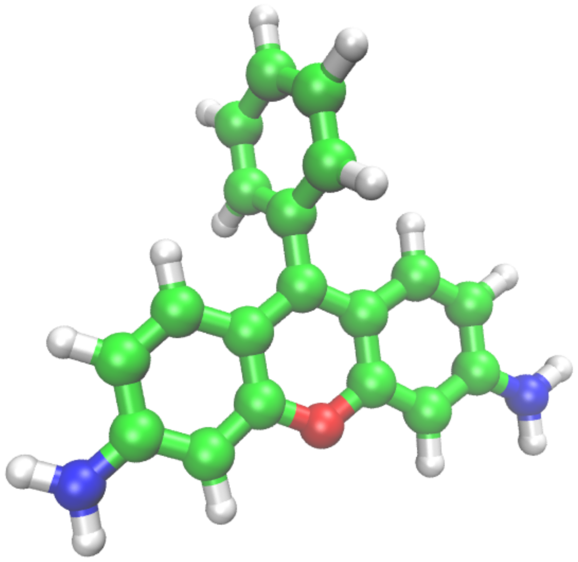

Figure S2: Rhodamine model used in our simulations

field inside the cavity at the beginning of the simulations (*i.e.*, $\mathbf{u}_y$ in Equation 10, Figure S1). In this set-up, a Rabi splitting energy of $\Omega_{\text{Rabi}} = 446$ meV was achieved. The integration time step was 0.1 fs for all cavity simulations, which were performed with Gromacs 4.5.3,[16] in which the multi-mode Tavis-Cummings model presented above, was implemented, using the QM/MM interface to TeraChem.[17,18]

2.5 Simulation Analysis

The absorption spectrum of the molecules was computed from trajectories in the ground (S_0) state. These spectra were composed of the S_1-S_0 energy gaps (1,000 snapshots) by super-position of Gaussian functions:[21]

$$I_{\text{mol}}^{\text{abs}}(E) \propto \sum_i^s \Delta E_i \exp[-\frac{(E-\Delta E_i)^2}{2\sigma^2}](\mu_i^{\text{TDM}})^2 \quad (14)$$

where $I_{\text{mol}}^{\text{abs}}(E)$ is the intensity of the molecular absorption as a function of excitation energy (E), s the number of snapshots included in the analysis, ΔE_i the excitation energy in snapshot i and μ_i^{TDM}

the transition dipole moment. A width of $\sigma = 0.05$ eV was chosen for the convolution.

Following Lidzey and coworkers,[22] we define the "visibility", I^m, of polaritonic state ψ^m as the total incoherent photonic contribution to that state (*i.e.*, $I^m \propto \sum_b^{n_{\max}} |\alpha_b^m|^2$). Thus, the k_z-dependent, one-photon absorption spectra of the strongly coupled molecule-cavity systems were computed as follows: For each frame of the trajectory, the polaritonic states were computed and the energy gaps of these states with respect to the overall ground state (*i.e.*, E^0, with all molecules in S_0, no photon in the cavity: $|S_0^1 S_0^2 ... S_0^N\rangle|0\rangle$) were extracted for all wave vectors, b, multiplied by $|\alpha_b^m|^2$ and summed up into a superposition of Gaussian functions:

$$I^{\text{abs}}(E,b) \propto \sum_i^s \left[\sum_m^{N+n_{\max}+1} |\alpha_{b,i}^m|^2 \exp[-\frac{(E-\Delta E_i^m)^2}{2\sigma^2}] \right] \tag{15}$$

Here, $I^{\text{abs}}(E,b)$ is the absorption intensity as a function of excitation energy E and in-plane momentum b ($k_z = 2\pi b/L_z$), s the number of trajectory frames included in the analysis, ΔE_i^m the excitation energy of polaritonic state m in frame i ($\Delta E_i^m = E_i^m - E_i^0$) and $\alpha_{b,i}^m$ the expansion coefficient of cavity mode b in polaritonic state m in that frame (equation 11). A width of $\sigma = 0.05$ eV was chosen for all convolutions in this work. The spectra were plotted with Mathematica, version 11.3.[23]

The photonic contribution of the total polaritonic wave function was computed as the sum of the projections of the cavity modes basis states (*i.e.*, $|S_0^1 S_0^2 .. S_0^N\rangle|1_b\rangle$) onto the time-dependent wave function:

$$\rho_{\text{pho}}(t) = \sum_{b=1}^{n_{\text{mode}}} \left| \langle 1_b | \langle S_0^1 S_0^2 .. S_0^N | \Psi(t) \rangle \right|^2 = \sum_{b=1}^{n_{\text{mode}}} \left| \sum_m^{N+n_{\text{mode}}} c_m(t) \alpha_b^m \right|^2 \tag{16}$$

where $N + n_{\text{mode}}$ is the number of polaritonic states.

2.6 Infra-Red, Raman and Franck-Condon spectra simulation

Infra-red and Raman spectra were obtained by performing a normal mode analysis, starting from the optimized molecular geometries in the ground and excited electronic states. The vibrational modes in the electronic ground state (S_0) were obtained by diagonalizing the Hessian at the CAM-

B3LYP/6-31G(d) level of theory, while the modes in the electronic excited state (S_1) were obtained by diagonalizing the Hessian at the TDA-CAM-B3LYP/6-31G(d) level of theory. The stick spectra were convoluted with a Gaussian function of width σ= 0.5 meV. Vibronic absorption and emission spectra were computed within the Franck-Condon approximation, which includes Duschinsky rotation, but no Herzberg-Teller contributions. We used the implementation of the generalized time-dependent approach for computing one-photon absorption (OPA) and emission (OPE) vibronic spectra by Liang *et al.*,[24] with the following parameters: temperature of 0 K, damping factor of 20 cm^{-1} and a time-domain propagation range of 40000 au. All calculations were performed with the Q-Chem 5.3 quantum chemistry package.[25]

We note that the normal modes in the S_0 and S_1 states are not fully identical. In addition to frequency shifts, some modes also mix, in the sense that the modes in the excited state are linear combinations of multiple modes in the ground state and *vice versa*. Nevertheless, inspection of the Duschinsky matrix confirmed that all mode displacements in the ground state that overlap with the non-adiabatic coupling vector, are linear combinations of modes that also overlap with the non-adiabatic coupling vector in the excited state. Therefore the conclusions of this work do not depend on whether excited or ground state modes are used.

3 Characterization of the lowest-energy dark state

Non-resonant pumping conditions were mimicked by starting the simulations in a linear superposition of the polaritonic states, in which the excitation is fully localized onto the first molecule. Although in experiment non-resonant excitation is typically achieved by pumping a higher-energy electronic state of the molecule, Kasha's rule suggests a rapid decay into the S_1 state,[26] and we therefore consider starting the simulations in the molecular S_1 state a reasonable approximation. The simulations were initiated with all molecules in their S_0 minimum energy geometry, and with zero kinetic energy. Simulations were performed with $N = 1$, 2, 4, 16, 32, 64 and 128 Tc molecules strongly coupled to a *single* confined light mode with energy $\hbar\omega_{\text{cav}} = 3.22$ eV. The Rabi splitting

kept constant at $\sim$ 429 meV for all systems by scaling the mode volume of the cavities, V_{cav}, with the number of molecules, N, at the start of the simulation (Table S1).

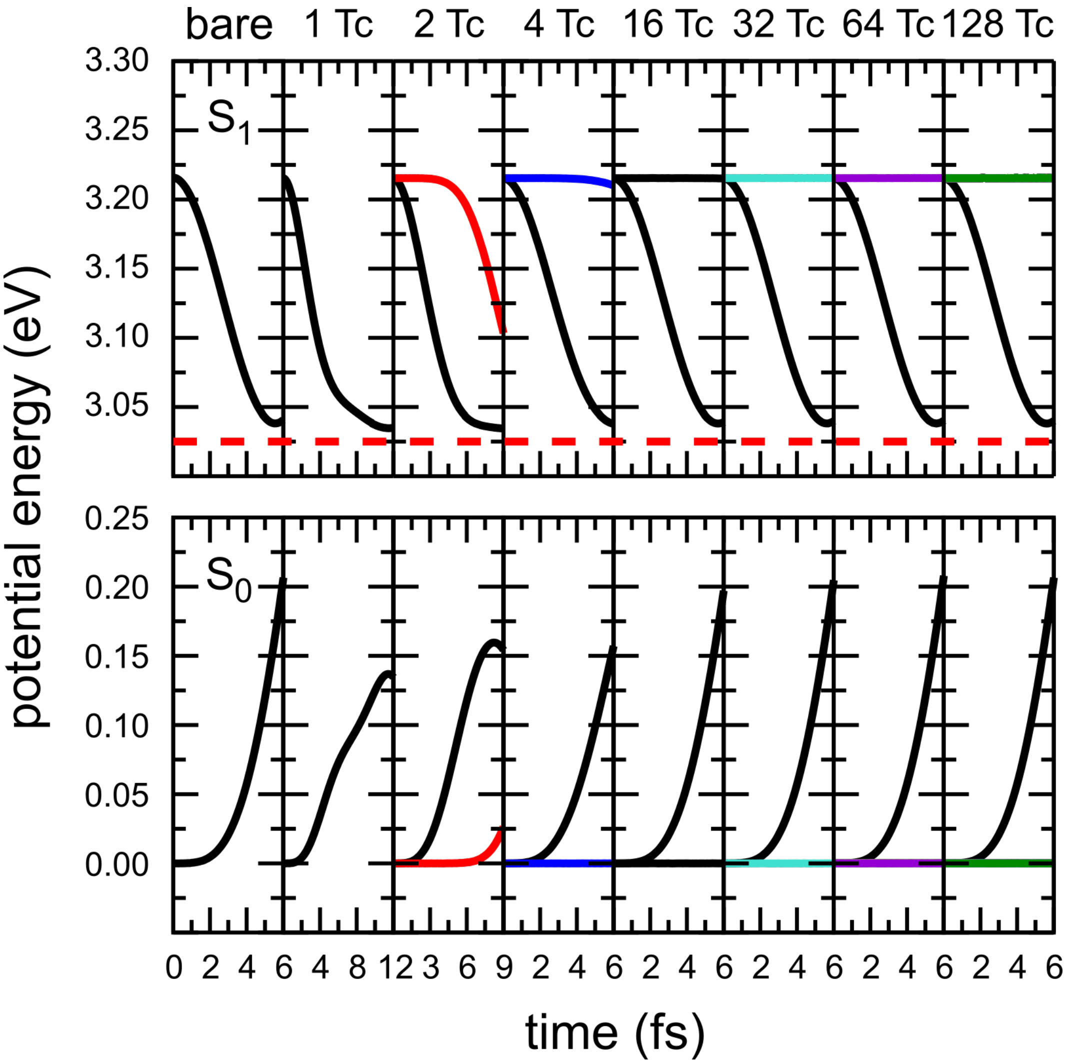

Figure S3: Evolution of the ground (S_0) and excited state (S_1) potential energy of a bare Tc molecule (*i.e.*, outside of the cavity), and of 1, 2, 4, 16, 32, 64 and 128 Tc molecules strongly coupled to a single-mode cavity resonant with the S_1 excitation energy of Tc. The vacuum field strength was adjusted to have the same Rabi splitting ($\sim$ 429 meV) in all cavity systems at the start of the simulation. The black line corresponds to the potential energy of molecule 1, the potential energies of the other molecules are shown in various colors. Note that because the molecules were all in the same configuration at the start of the simulation, their S_0 and S_1 potential energies remain similar and the curves overlap. The red dotted line indicates the energy value of the S_1 minimum, obtained by geometry optimization of the bare molecule in the S_1 state.

The evolution of the potential energy of the Tc molecules is plotted in Figure S3 for a bare molecule (*i.e.*, outside the cavity) as well as for 1, 2, 4, 16, 32, 64 and 128 molecules inside the

Table 1: Contribution of the excitation on the molecule in the S_1 minimum energy geometry (molecule 1) to the lowest energy dark state $|\beta_1^{DS_0}|^2$ when all other molecules are in the S_0 minimum. Cavity volumes, V_{cav}, were scaled by the number of molecules, N, to have a Rabi splitting of $\hbar\Omega^{Rabi} = 429$ meV when all molecules are in the S_0 geometry

N	V_{cav} (nm^3)	$\lvert\beta_1^{DS_0}\rvert^2$
16	15.6	0.950
32	31.1	0.972
64	62.3	0.985
128	124.5	0.992
256	249.1	0.996
512	498.1	0.998
1024	996.2	0.999
2048	1969.1	1.000

cavity. The plots suggest that after non-resonant excitation into the S_1 electronic state of the first molecule, this molecule relaxes towards a minimum on its S_1 potential energy surface (dashed line) within 6-12 fs. Because, in contrast to previous work,[2,3] no molecular environment was included here to avoid obscuring the interpretation, the molecular dynamics are under damped. Therefore, the Tc molecule does not get trapped, but rather oscillates around the minimum on the S_1 potential energy surface instead. Nevertheless, the results of the simulations suggest that inside the cavity, the local S_1 minimum is accessible to molecules that are non-resonantly excited, irrespective of the number of molecules, or cavity field strength, in line with results from previous simulations.[27,28]

Assuming, therefore, that dissipation will eventually dampen the dynamics and trap the excited Tc molecule in the S_1 minimum geometry, while the other molecules remain in their S_0 minimum geometries, we inspected the eigenstates of the Tavis-Cummings Hamiltonian in this combination of molecular configurations. The contribution of the molecule in the S_1 minimum energy geometry (molecule 1) to the lowest energy dark state, $|\beta_1^{DS_0}|^2$ (Equation 11), listed in Table 1, rapidly converges to unity upon increasing the number of molecules.

4 Analysis of non-adiabatic coupling vector

The non-adiabatic coupling vector connecting displacements in the vibrational modes of molecule i to transitions between polaritonic states ψ^l and ψ^m can be expressed as a Hellmann-Feynmann force:

$$\mathbf{d}_{ml} = \langle \psi^m | \nabla_{a\in i} | \psi^l \rangle = \frac{\langle \psi^m | \nabla_{a\in i} \hat{H}^{\text{TC}} | \psi^l \rangle}{E_l - E_m} \tag{17}$$

with $\nabla_{a\in i}$ the gradient with respect to the displacement of an atom a in molecule i and E_l the adiabatic energy of polaritonic state l. After substitution of the expression for the polaritonic eigenstates ψ^m (Equation 11), the Hellman-Feynman term becomes:[3]

$$\begin{aligned}\langle \psi^m | \nabla_{a\in j} \hat{H}^{\text{TC}} | \psi^l \rangle \;=\;& (\beta_j^m)^* \beta_j^l \nabla_{a\in j} V_{\text{S}_1}^{\text{mol}}(\mathbf{R}_j) + \nabla_{a\in j} V_{\text{S}_0}^{\text{mol}}(\mathbf{R}_j) \times \left[\delta_{ml} - (\beta_j^m)^* \beta_j^l\right] + \\ & (\beta_j^m)^* \nabla_{a\in j} \boldsymbol{\mu}_j \cdot \mathbf{u}_{\text{cav}} \sum_b^{n_{\max}} (\alpha_b^l) \sqrt{\frac{\hbar\omega_{\text{cav}}(2\pi b/L_y)}{2\epsilon_0 V_{\text{cav}}}} e^{2\pi i b y_j/L_y} + \\ & \beta_j^l \nabla_{a\in j} \boldsymbol{\mu}_j \cdot \mathbf{u}_{\text{cav}} \sum_b^{n_{\max}} (\alpha_b^m)^* \sqrt{\frac{\hbar\omega_{\text{cav}}(2\pi b/L_y)}{2\epsilon_0 V_{\text{cav}}}} e^{-2\pi i b y_j/L_y}\end{aligned} \tag{18}$$

In what follows, we assume a single-mode cavity, *i.e.* $n_{\text{mode}} = 1$. We also distinguish between dark states, which lack a photonic component (*i.e.*, $\alpha_b^m = 0$) and bright states, which contain a photonic component (*i.e.*, $\alpha_b^m \neq 0$). Although such strict distinction is possible only in absence of any disorder, it provides a conceptually simpler description for analysing the non-adiabatic coupling vector in Equation 17.

With these simplifications, we can derive an expression for the non-adiabatic coupling vector between the lower polariton (LP) and the relaxed dark state (DS_0), in which molecule 1 is in the S_1 geometry, while all other molecules are in their S_0 geometries. For sufficiently large ensembles of molecules, the coefficient associated with the excitation on the first molecule in the DS_0 state is one ($\beta_1^{\text{DS}_0} = 1$, Table 1, main text), while the coefficients associated with the other molecules are zero ($\beta_{i\neq 1}^{\text{DS}_0} = 0$) as well as the coefficient associated with the excitation of the single cavity mode ($\alpha^{\text{DS}_0} = 0$, Equation 11). Under these hypothetical conditions, the molecules in the S_0 geometry

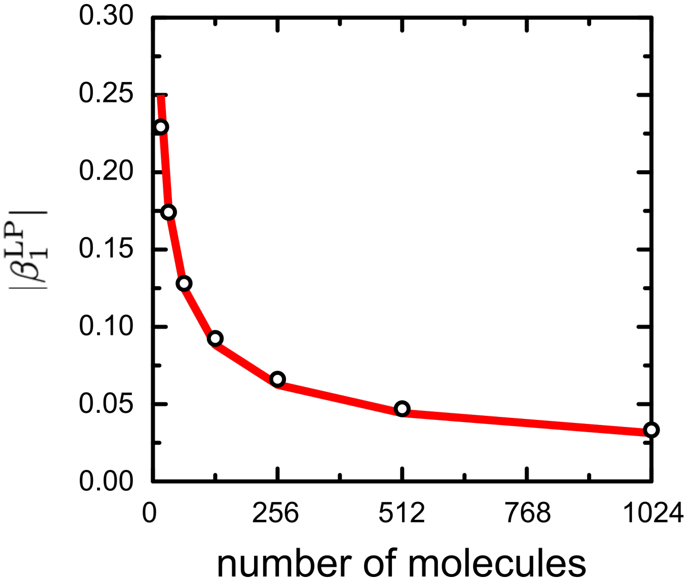

Figure S4: Expansion coefficient associated with the Tc molecule in the S_1 minimum energy geometry ($\beta_1^{\rm LP}$), in the lower polariton state for molecules in a single-mode cavity. The other Tc molecules are in the S_0 minimum energy geometry. Dots are the $|\beta_1^{\rm LP}|$ values; red curve is a fit to $a/\sqrt{N} + b$ with $a = 0.90$ and $b = 0.01$.

contribute equally to the lower polariton with a coefficient proportional to $1/\sqrt{2N}$. The contribution of the molecule in the S_1 state is slightly different because its energy gap to the ground state is smaller. Nevertheless, the contribution of this molecule to the LP is still inversely proportional to $\sqrt{N}$, as we show in Figure S4. With these assumptions, the Hellmann-Feynman term between the relaxed dark state (DS_0) and the LP, associated with a displacement of an atom in any of the molecules that are in the S_0 minimum, reduces to:

$$\begin{aligned}
\langle \psi^{\rm LP} | \nabla_{a \in j \neq 1} \hat{H}^{\rm TC} | \psi^{\rm DS_0} \rangle &= (\beta_{j\neq 1}^{\rm LP})^* \beta_{j\neq 1}^{\rm DS_0} \nabla_{a\in j \neq 1} V_{\rm S_1}^{\rm mol}(\mathbf{R}_j) - (\beta_{j\neq 1}^{\rm LP})^* \beta_{j\neq 1}^{\rm DS_0} \nabla_{a\in j \neq 1} V_{\rm S_0}^{\rm mol}(\mathbf{R}_j) - \\
&\quad (\beta_{j\neq 1}^{\rm LP})^* \nabla_{a\in j\neq 1} \boldsymbol{\mu}_j \cdot \mathbf{u}_{\rm cav} (\alpha^{\rm DS_0}) \sqrt{\frac{\hbar\omega_{\rm cav}}{2\epsilon_0 V_{\rm cav}}} - \\
&\quad \beta_{j\neq 1}^{\rm DS_0} \nabla_{a\in j\neq 1} \boldsymbol{\mu}_j \cdot \mathbf{u}_{\rm cav} (\alpha^{\rm LP})^* \sqrt{\frac{\hbar\omega_{\rm cav}}{2\epsilon_0 V_{\rm cav}}} \\
&= 0
\end{aligned} \tag{19}$$

In contrast, for a displacement of an atom in the molecule that is in the S_1 minimum (*i.e.*, molecule 1) the Hellmann-Feynman term becomes:

$$
\begin{aligned}
\langle\psi^{\text{LP}}|\nabla_{a\in 1}\hat{H}^{\text{TC}}|\psi^{\text{DS}_0}\rangle &= (\beta_1^{\text{LP}})^*\beta_1^{\text{DS}_0}\nabla_{a\in 1}V_{\text{S}_1}^{\text{mol}}(\mathbf{R}_1) - (\beta_1^{\text{LP}})^*\beta_1^{\text{DS}_0}\nabla_{a\in 1}V_{\text{S}_0}^{\text{mol}}(\mathbf{R}_1) - \\
& (\beta_1^{\text{LP}})^*\nabla_{a\in 1}\boldsymbol{\mu}_1\cdot\mathbf{u}_{\text{cav}}(\alpha^{\text{DS}_0})\sqrt{\tfrac{\hbar\omega_{\text{cav}}}{2\epsilon_0 V_{\text{cav}}}} - \\
& \beta_1^{\text{DS}_0}\nabla_{a\in 1}\boldsymbol{\mu}_1\cdot\mathbf{u}_{\text{cav}}(\alpha^{\text{LP}})^*\sqrt{\tfrac{\hbar\omega_{\text{cav}}}{2\epsilon_0 V_{\text{cav}}}} \qquad (20)\\
&= \tfrac{0.9}{\sqrt{N}}\left[\nabla_{a\in 1}V_{\text{S}_1}^{\text{mol}}(\mathbf{R}_1) - \nabla_{a\in 1}V_{\text{S}_0}^{\text{mol}}(\mathbf{R}_1)\right] - \\
& \sqrt{\tfrac{1}{2}}\nabla_{a\in 1}\boldsymbol{\mu}_1\cdot\mathbf{u}_{\text{cav}}\sqrt{\tfrac{\hbar\omega_{\text{cav}}}{2\epsilon_0 V_{\text{cav}}}}
\end{aligned}
$$

where the factor 0.9 was obtained from the fit to β_1^{LP} in Figure S4. This analysis suggests that the non-adiabatic coupling vector for transitions between the DS_0 and LP states is dominated by atomic displacements of the molecule that is in the S_1 geometry.

To verify this result, we performed MD simulations of 16 tetracene molecules, strongly coupled to a single-mode cavity with a cavity resonance $\hbar\omega_{\text{cav}} = 3.22$ eV and a cavity volume $V_{\text{cav}} = 15.6$ nm^3. The simulations were started in the DS_0 state with one of the tetracene molecules in the S_1 geometry, and all others in the S_0 geometry. Initial velocities were randomly selected from a Maxwell-Boltzmann distribution at 300 K. Two simulations were performed: in first simulation, the atoms of the molecule that is in the S_1 minimum (molecule 1), were kept fixed, whereas in the second simulation the atoms of all molecules that are in the S_0 geometry, were frozen. In Figure S5 we plot the population of the LP during the simulations. As shown in Figure S5a, there is no population transfer if the molecule that is in the S_1 geometry, is frozen. In contrast, when the other molecules are frozen, there is population transfer from the DS_0 state into the LP (Figure S5b). These results thus confirm that only displacements of atoms in the molecule that is in the S_1 state, can drive non-adiabatic transitions between the DS_0 and LP states.

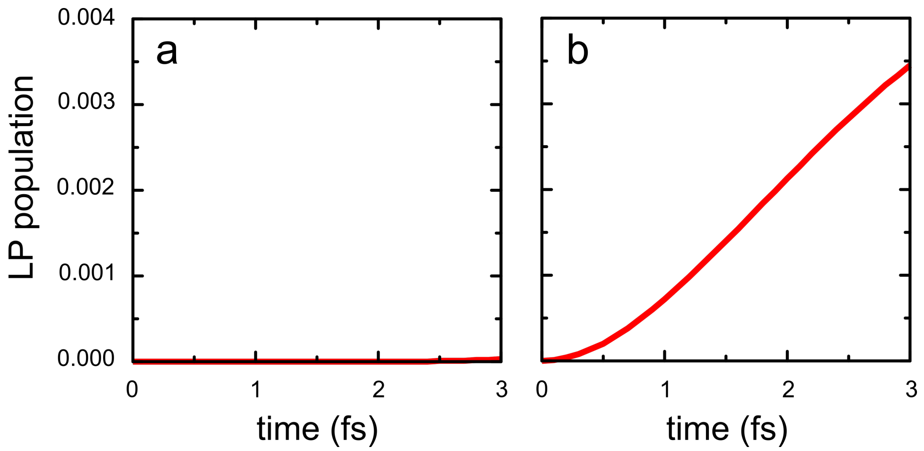

Figure S5: Population of the lower polariton (LP) in simulations of 16 tetracene molecules strongly coupled to a single-mode cavity, starting in the lowest energy dark state (DS_0) with one molecule in the S_1 minimum energy geometry, and all other molecules in the S_0 minimum energy geometry. In panel a, the molecule in the S_1 minimum energy geometry is kept frozen, whereas in panel b, all molecules that are in the S_0 minimum energy geometry, are frozen.

Because molecule 1 is in its S_1 minimum energy geometry, while all other molecules are in their S_0 minimum energy geometries (*i.e.*, $\nabla_{a\in 1}V_{S_1}^{mol}(\mathbf{R}_1) = 0$ and $\nabla_{a\in j}V_{S_0}^{mol}(\mathbf{R}_j)$=0 for $j \neq 1$), the Hellmann-Feynman term in Equation 20 can be simplified further:

$$\langle\psi^{LP}|\nabla_{a\in 1}\hat{H}^{TC}|\psi^{DS_0}\rangle = -\frac{0.9}{\sqrt{N}}\nabla_{a\in 1}V_{S_0}^{mol}(\mathbf{R}_1) - \sqrt{\frac{1}{2}}\nabla_{a\in 1}\boldsymbol{\mu}_1\cdot\mathbf{u}_{cav}\sqrt{\frac{\hbar\omega_{cav}}{2\epsilon_0 V_{cav}}} \quad (21)$$

Substituting this expression into equation 17 yields the non-adiabatic coupling vector connecting the lowest energy dark state to the lower polariton:

$$\langle\psi^{LP}|\nabla_{a\in 1}|\psi^{DS_0}\rangle = \frac{1}{E_{LP}-E_{DS_0}}\left(\frac{0.9}{\sqrt{N}}\nabla_{a\in 1}V_{S_0}^{mol}(\mathbf{R}_1) + \sqrt{\frac{1}{2}}\nabla_{a\in 1}\boldsymbol{\mu}_1\cdot\mathbf{u}_{cav}\sqrt{\frac{\hbar\omega_{cav}}{2\epsilon_0 V_{cav}}}\right) \quad (22)$$

The first term on the right hand side suggest that in order to mediate non-adiabatic population transfer between the DS_0 and LP, the molecular vibrations must have a geometric displacement, or Huang-Rhys factor, between the electronic ground (S_0) and excited (S_1) states. Because such modes also are Franck-Condon active, they show up in vibronically resolved absorption and emission spectra. The first term scales inversely with the square root of N. In contrast, the second term involving the cavity vacuum field and transition dipole moment of the molecules, is independent

of N. Even if the number of molecules that fit inside the mode volume of a real Fabry-Pérot is finite, N can be very large and it is therefore unclear which of these two terms would dominate the non-adiabatic coupling vector in experiment. Nevertheless, as shown in Figure S6, both terms overlap with the same vibrational modes of tetracene. Therefore, to reach the main conclusion of this work that specific vibrations control the non-adiabatic relaxation from the dark states into the lower polariton, it is irrelevant which term dominates the non-adiabatic coupling.

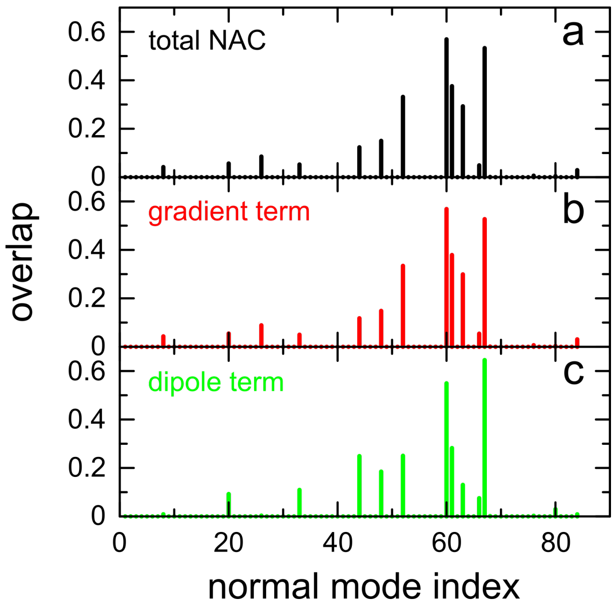

Figure S6: Overlaps between the normalized vibrational normal modes of tetracene and the normalized full non-adiabatic coupling vector (a), the normalized gradient contribution to the non-adiabatic coupling vector (b, first term on right hand side of equation 22), and the transition dipole moment contribution to the non-adiabatic coupling vector (c, second term on the right hand side of equation 22).

We note that because the energy gap between DS_0 and LP is proportional to the Rabi splitting, the prefactor also scales as $1/\sqrt{N}$. However, for comparing different ensemble sizes in this work, we kept the Rabi splitting the same by scaling the mode volume of the cavity V_{cav} with N: $V_{\text{cav}} = V_{\text{cav}}^{(16)} N/16$, with $V_{\text{cav}}^{(16)}$ the mode volume used for simulations with 16 molecules in the

cavity. Therefore,

$$\begin{aligned}\langle\psi^{\rm LP}|\nabla_{a\in 1}|\psi^{\rm DS_0}\rangle &= \frac{1}{E_{\rm LP}-E_{\rm DS_0}}\left(\frac{0.9}{\sqrt{N}}\nabla_{a\in 1}V_{\rm S_0}^{\rm mol}(\mathbf{R}_1)+\sqrt{\frac{1}{2}}\nabla_{a\in 1}\boldsymbol{\mu}_1\cdot\mathbf{u}_{\rm cav}\sqrt{\frac{\hbar\omega_{\rm cav}}{2\epsilon_0 V_{\rm cav}^{(16)}N/16}}\right)\\ &= \frac{1}{\sqrt{N}}\frac{1}{E_{\rm LP}-E_{\rm DS_0}}\left(0.9\nabla_{a\in 1}V_{\rm S_0}^{\rm mol}(\mathbf{R}_1)+\sqrt{\frac{1}{2}}\nabla_{a\in 1}\boldsymbol{\mu}_1\cdot\mathbf{u}_{\rm cav}\sqrt{\frac{8\hbar\omega_{\rm cav}}{\epsilon_0 V_{\rm cav}^{(16)}}}\right)\end{aligned} \tag{23}$$

By keeping the Rabi splitting the same, also the energy gap remains the same. Hence, at *constant* Rabi splitting, the non-adiabatic coupling vector scales with $1/\sqrt{N}$. To verify this result, we computed the norm of this vector at the start of the simulation for various ensemble sizes. These norms are plotted in Figure S7 together with the best fit to a function $f(N) = a/\sqrt{N}$. The reasonably good fit suggests that for a constant Rabi splitting, the non-adiabatic coupling vector is indeed proportional to the inverse square root of the number of molecules strongly coupled to the cavity.

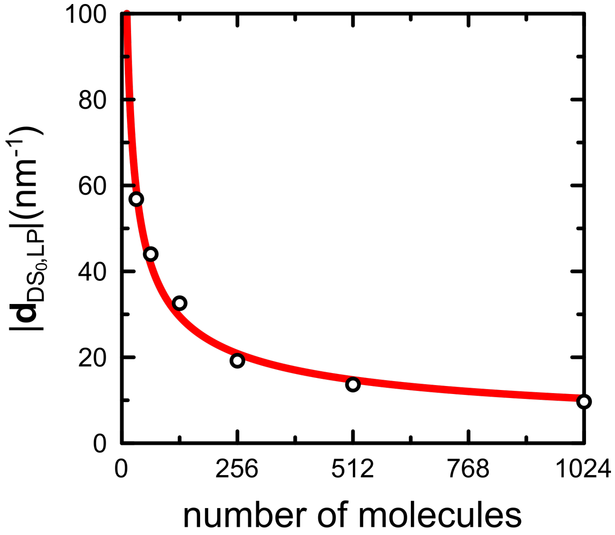

Figure S7: Norm of the non-adiabatic coupling vector between the lower polariton (LP) and lowest energy dark state (DS_0), in which one Tc molecule is in the S_1 minimum energy geometry, while all other Tc molecules are in the S_0 minimum energy geometry. The open dots are the calculated norms at the start of the simulation; the red line is a fit to $a/\sqrt{N}$ with $a = 337.74$ nm^{-1}.

5 Relaxation in a strongly coupled Rhodamine-cavity system

In Figure S8a we plot the overlap between the non-adiabatic coupling vector connecting the DS_0 state and LP on the one hand, and the vibrational modes of Rhodamine on the other hand, as a function of the vibrational energy. To understand which of the vibrational modes can induce population transfer between the DS_0 and LP, we performed 105 short MD simulations of a cavity with 16 Rh molecules. These simulations were initiated in the lowest energy dark state, DS_0, in which the first Rh molecule is in the S_1 minimum geometry, while the other 15 molecules are in the S_0 geometry. In each simulation, we selectively activated one of the 105 vibrational modes of the molecule in the S_1 minimum energy geometry by providing as initial atomic velocities the mass-unweighted normal mode vector, which was obtained within the harmonic approximation by diagonalizing the molecular Hessian. This vector was multiplied by a factor 5, such that the initial temperature was around 300 K.

To quantify the extent of population transfer from the DS_0 state into the more photonic LP state during the simulation, we projected the excitation of the single cavity mode (*i.e.*, $\langle 1|\langle S_0^1 S_0^2 .. S_0^{N-1} S_0^N|$, Equation 11) onto the total time-dependent polaritonic wave function ($|\Psi(t)\rangle$, Equation 12). The top panel in Figure S8b shows this photonic weight (*i.e.*, $|\sum_m \sum_j c_m \alpha_j^m|^2$) at 1 fs as a function of the vibrational energy of the mode along which the initial velocities were directed. The observation that population transfer predominantly occurs if we activate vibrational modes that overlap with the non-adiabatic coupling vector, suggests that also for Rhodamine, which lacks internal symmetry, the relaxation from the dark state manifold into the LP is selectively mediated by these vibrations.

As for Tetracene, we also computed the IR, Raman and vibronic spectra (Figure S8). In contrast to Tetracene, not all modes that can drive population transfer, are Raman-active. Therefore, for Rhodamine, which lacks internal symmetry, the Raman spectrum is less useful for predicting such modes. Instead, these modes can only be identified from their overlap with the non-adiabatic coupling vector.

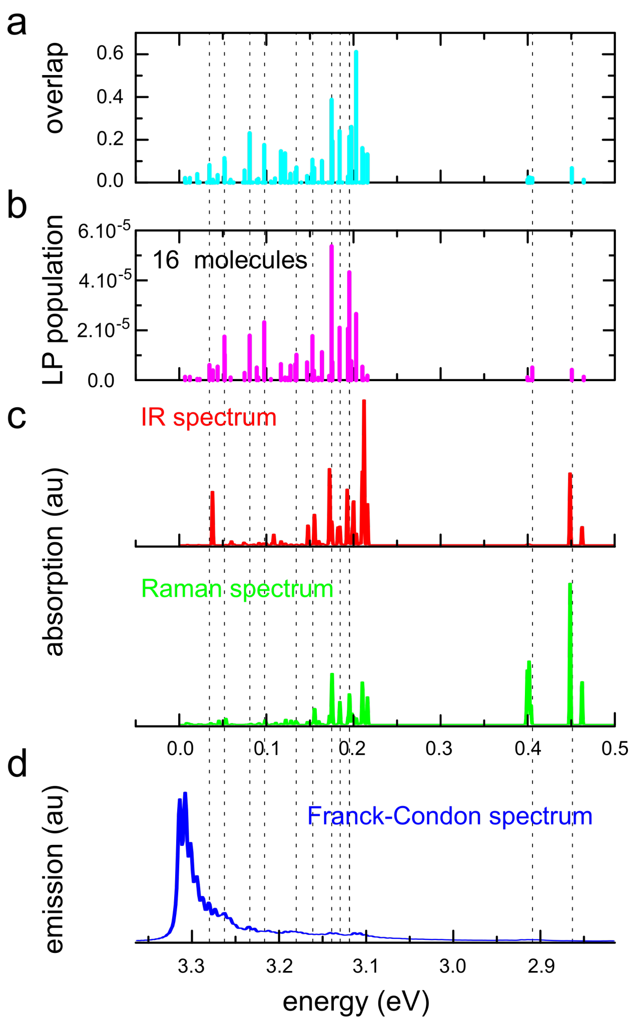

Figure S8: Panel a: Overlap between the non-adiabatic coupling vector $\mathbf{d}_{\mathrm{DS}_0,LP}$ and the vibrational modes of Rhodamine, plotted as a function of their energies. Panel b: Total photonic weight ($|\sum_m \sum_j c_m \alpha_j^m|^2$) at 1 fs in simulations of 16 Rh molecules coupled to a single-mode cavity. The 105 trajectories were started in the DS_0 state, with initial atomic velocities along one of the vibrational modes of the Rh molecule in the S_1 geometry. Panel c: calculated Infra-red (IR), and Raman spectra of Rh. Panel d: calculated Franck-Condon fluorescence spectrum, plotted with an inverted x-axis to match the normal mode energies. The dashed vertical lines are a guide for the eye.

6 Model system for vibronic transitions between polaritons

To understand if in addition to overlap with the non-adiabatic coupling vector, the vibrational mode energy also plays a role in the relaxation from the DS_0 state into the LP, we constructed a simple model system. The model, illustrated in Figure S9, contains two polaritonic states, described by harmonic potentials. The minima of these potentials are separated in energy by $\Delta E_{\mathrm{LP,DS}_0}$, the gap

between the LP and DS_0 states. Within the Born representation, the wave function of this model system is:

$$\Psi = \sum_m \chi_m \psi^m \tag{24}$$

where ψ^m are the eigenstates of the Tavis-Cummings Hamiltonian ($\hat{H}^{\text{TC}}$), here restricted to ψ^{LP} and ψ^{DS_0}. The associated potential energy surfaces (V_m) are modeled as harmonic potentials:

$$V_m = \frac{1}{2} M \omega_m^2 (x - x_{0m})^2 \tag{25}$$

with ω_m the frequency of the vibration in ψ^m and x_{0m} the position of the minimum of the harmonic potential in polaritonic state m and M the effective mass. The vibrational functions $|\chi_m\rangle$ are the vibrational eigenstates in these harmonic potentials, *i.e*: $|0\rangle_{\text{LP}}$, $|1\rangle_{\text{LP}}$, .. $|n\rangle_{\text{LP}}$ and $|0\rangle_{\text{DS}_0}$, $|1\rangle_{\text{DS}_0}$, ... $|n\rangle_{\text{DS}_0}$ with energies $E_n^{\text{LP}} = (n + 1/2)\hbar\omega_{\text{LP}}$ and $E_n^{\text{DS}_0} = (n + 1/2)\hbar\omega_{\text{DS}_0}$. The Schrödinger equation for this system is

$$\begin{aligned} \hat{H}^{\text{tot}}\Psi &= E\Psi \\ \textstyle\sum_m \left(\hat{T}_N \chi_m \psi^m + \hat{H}^{\text{TC}} \psi^m \chi_m \right) &= E \textstyle\sum_m \chi_m \psi^m \end{aligned} \tag{26}$$

with $\hat{T}_N$ the nuclear kinetic energy operator, and $\hat{H}^{\text{TC}}$ the Tavis-Cummings Hamiltonian. Multiplying from the left with polaritonic state $\langle \psi^l |$ yields:

$$\begin{aligned} \textstyle\sum_m \langle \psi^l | \hat{T}_N | \psi^m \rangle |\chi_m\rangle + V_l |\chi_l\rangle &= E|\chi_l\rangle \\ -\textstyle\sum_m \frac{\hbar^2}{M} \langle \psi^l | \nabla_N | \psi^m \rangle \nabla_N |\chi_m\rangle + \left[-\frac{\hbar}{2M} \frac{\partial^2}{\partial x^2} + \frac{1}{2} M \omega_l^2 (x - x_{0l})^2 \right] |\chi_l\rangle &= E|\chi_l\rangle \\ -\textstyle\sum_m \frac{\hbar^2}{M} \mathbf{d}_{lm} \cdot \nabla_N |\chi_m\rangle + \hbar\omega_l (n_l + \frac{1}{2}) |\chi_l\rangle &= E|\chi_l\rangle \end{aligned} \tag{27}$$

where we omitted the $\langle \psi^n | \nabla_N^2 | \psi^m \rangle$ term, because it is much smaller than $\langle \psi^n | \nabla_N | \psi^m \rangle$. The quantum number n_l indicates the eigenstates of the harmonic oscillator in polaritonic state ψ^l. The

non-adiabatic coupling between the polaritonic states is conveniently evaluated using the stepping operators:

$$-\frac{\hbar^2}{M}\mathbf{d}_{lm}\nabla_N|n\rangle = -\sqrt{\frac{\hbar\omega}{2M}}\mathbf{d}_{lm}(\hat{a}-\hat{a}^\dagger)|n\rangle \tag{28}$$

where $\hat{a}|n\rangle = \sqrt{n}|n-1\rangle$ and $\hat{a}^\dagger|n\rangle = \sqrt{n+1}|n+1\rangle$ and we introduced the short-hand notation $|n\rangle$ for $|\chi_n\rangle$.

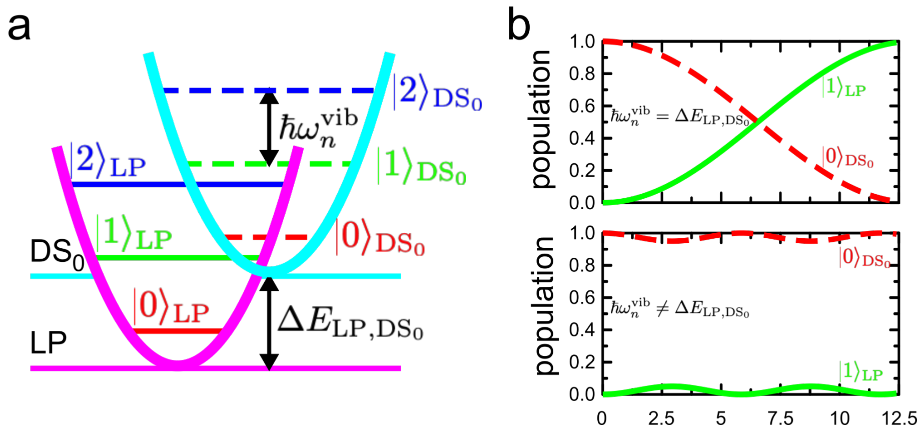

Figure S9: Panel a illustrates the model for vibrational assisted relaxation from the dark state manifold into the LP. The polaritonic potential energy surfaces of the LP and lowest energy dark state (DS_0) with one molecule in the S_1 minimum energy geometry and the other molecules in the S_0 geometry, are modeled as harmonic functions. The vibrational eigenstates are indicated. The simulations are initiated in the lowest vibrational energy eigenstate $|0\rangle_{\mathrm{DS}_0}$ of the molecule in the S_1 geometry on the DS_0 potential. The plots in panel b show the population of the $|0\rangle_{\mathrm{DS}_0}$ and $|1\rangle_{\mathrm{LP}}$ vibrational states as a function of simulation time when the energy gap between the LP and DS_0 ($\Delta E_{\mathrm{LP,DS}_0}$) is the same as the energy spacing ($\hbar\omega^{\mathrm{vib}}$) of the vibrational mode (top), and when the energy gap is not the same as energy spacing of that vibrational mode (bottom)

Using a maximum of 4 vibrational eigenstates in both LP and DS_0 potentials, we solved the time-dependent Schrödinger equation for this system, starting in $|0\rangle_{\mathrm{DS}_0}$. This initial condition was obtained as a linear combination of the eigenstates of the total Hamiltonian (Equation 26). We performed two simulations: in the first simulation the energy gap between the DS_0 and LP state was identical to the energy gap between the vibrational eigenstates in the LP state ($\hbar\omega_{\mathrm{LP}} = E_{\mathrm{DS}_0} - E_{\mathrm{LP}}$), while in the second simulation, the energy gaps differed by 1 meV ($\hbar\omega_{\mathrm{LP}} \neq E_{\mathrm{DS}_0} - E_{\mathrm{LP}}$). Vibrational frequency and mass were obtained from the normal mode analysis, while the magnitude of the non-adiabatic coupling vector was extracted from the simulation with 512 tetracene molecules

strongly coupled to the single-mode cavity. Figure S9b shows how the population of the vibrational states $|0\rangle_{\mathrm{DS}_0}$ and $|1\rangle_{\mathrm{LP}}$ evolve during the simulations. Efficient population transfer from the ground vibrational state in the lowest energy dark state (DS_0) into the first vibrational excited state in the LP occurs efficiently only if the energy gaps match (top panel in Figure S9b). In contrast, when the gaps are different, transfer is inefficient (bottom panel in Figure S9b)

References

(1) Luk, H.-L.; Feist, J.; Toppari, J. J.; Groenhof, G. Multiscale Molecular Dynamics Simulations of Polaritonic Chemistry. *J. Chem. Theory Comput.* **2017**, *13*, 4324–4335.

(2) Groenhof, G.; Climent, C.; Feist, J.; Morozov, D.; Toppari, J. J. Tracking Polariton Relaxation with Multiscale Molecular Dynamics Simulations. *J. Chem. Phys. Lett.* **2019**, *10*, 5476–5483.

(3) Tichauer, R. H.; Feist, J.; Groenhof, G. Multiscale simulations of molecular polaritons: the effect of multiple cavity modes on polariton relaxation. *J. Chem. Phys.* **2021**, *154*, 104112.

(4) Jaynes, E. T.; Cummings, F. W. Comparison of quantum and semiclassical radiation theories with to the beam maser. *Proc. IEEE* **1963**, *51*, 89–109.

(5) Tavis, M.; Cummings, F. W. Approximate solutions for an N-molecule radiation-field Hamiltonian. *Phys. Rev.* **1969**, *188*, 692–695.

(6) Michetti, P.; Rocca, G. C. L. Polariton states in disordered organic microcavities. *Phys. Rev. B.* **2005**, *71*, 115320.

(7) Tully, J. C. Nonadiabatic molecular dynamics. *Int.J. Quant. Chem.* **1991**, *25*, 299–309.

(8) Ehrenfest, P. Bemerkung über die angenäherte Gültigkeit der klassischen Mechanik innerhalb der Quantenmechanik. *Z. Phys.* **1927**, *45*, 445–457.

(9) Granucci, G.; Persico, M.; Toniolo, A. Direct semiclassical simulation of photochemical processes with semiempirical wave functions. *J. Chem. Phys.* **2001**, *114*, 10608–10615.

(10) Hohenberg, P.; Kohn, W. Imhomogeneous Electron Gas. *Phys. Rev.* **1964**, *136*, 864–871.

(11) Becke, A. D. A new mixing of Hartree-Fock and local density-functional theories. *J. Chem. Phys.* **1993**, *98*, 1372.

(12) Yanai, T.; Tew, D. P.; Handy, N. C. A new hybrid exchange-correlation functional using the Coulomb-attenuating method (CAM-B3LYP. *Chem. Phys. Lett.* **2004**, *393*, 51–57.

(13) Ditchfield, R.; Hehre, W. J.; Pople, J. A. Self-Consistent Molecular-Orbital Methods. IX. An Extended Gaussian-Type Basis for Molecular-Orbital Studies of Organic Molecules. *J. Chem. Phys.* **1971**, *54*, 724–728.

(14) Hirata, S.; Head-Gordon, M. Time-dependent density functional theory within the Tamm–Dancoff approximation. *Chem. Phys. Lett.* **1999**, *314*, 291–299.

(15) Bussi, G.; Donadio, D.; Parrinello, M. Canonical sampling through velocity rescaling. *J. Chem. Phys.* **2007**, *126*, 014101.

(16) Hess, B.; Kutzner, C.; van der Spoel, D.; Lindahl, E. GROMACS 4: Algorithms for Highly Efficient, Load-Balanced, and Scalable Molecular Simulation. *J. Chem. Theory Comput.* **2008**, *4*, 435–447.

(17) Ufimtsev, I.; Martínez, T. J. Quantum Chemistry on Graphical Processing Units. 3. Analytical Energy Gradients and First Principles Molecular Dynamics. *J. Chem. Theory Comput.* **2009**, *5*, 2619–2628.

(18) Titov, A.; Ufimtsev, I.; Luehr, N.; Martínez, T. J. Generating Efficient Quantum Chemistry Codes for Novel Architectures. *J. Chem. Theory Comput.* **2013**, *9*, 213–221.

(19) Frisch, M. J.; Trucks, G. W.; Schlegel, H. B.; Scuseria, G. E.; Robb, M. A.; Cheeseman, J. R.; Scalmani, G.; Barone, V.; Petersson, G. A.; Nakatsuji, H.; Li, X.; Caricato, M.; Marenich, A. V.; Bloino, J.; Janesko, B. G.; Gomperts, R.; Mennucci, B.; Hratchian, H. P.; Ortiz, J. V.; Izmaylov, A. F.; Sonnenberg, J. L.; Williams-Young, D.; Ding, F.; Lipparini, F.; Egidi, F.;

Goings, J.; Peng, B.; Petrone, A.; Henderson, T.; Ranasinghe, D.; Zakrzewski, V. G.; Gao, J.; Rega, N.; Zheng, G.; Liang, W.; Hada, M.; Ehara, M.; Toyota, K.; Fukuda, R.; Hasegawa, J.; Ishida, M.; Nakajima, T.; Honda, Y.; Kitao, O.; Nakai, H.; Vreven, T.; Throssell, K.; Montgomery, J. A., Jr.; Peralta, J. E.; Ogliaro, F.; Bearpark, M. J.; Heyd, J. J.; Brothers, E. N.; Kudin, K. N.; Staroverov, V. N.; Keith, T. A.; Kobayashi, R.; Normand, J.; Raghavachari, K.; Rendell, A. P.; Burant, J. C.; Iyengar, S. S.; Tomasi, J.; Cossi, M.; Millam, J. M.; Klene, M.; Adamo, C.; Cammi, R.; Ochterski, J. W.; Martin, R. L.; Morokuma, K.; Farkas, O.; Foresman, J. B.; Fox, D. J. Gaussian~16 Revision C.01. 2016; Gaussian Inc. Wallingford CT.

(20) Runge, E.; Gross, E. K. U. Density-Functional Theory for Time-Dependent Systems. *Phys. Rev. Lett* **1984**, *52*, 997–1000.

(21) Modi, V.; Donnini, S.; Groenhof, G.; Morozov, D. Protonation of the Biliverdin IXα Chromophore in the Red and Far- Red Photoactive States of a Bacteriophytochrome. *J. Phys. Chem. B* **2019**, *123*, 2325–2334.

(22) Lidzey, D.; Bradley, D.; Armitage, A.; Walker, S.; Skolnick, M. Photon-Mediated Hybridization of Frenkel Excitons in Organic Semiconductor Microcavities. *Science* **2000**, *288*, 1620–1623.

(23) Inc., W. R. Mathematica, Version 11.3. Champaign, IL, 2018.

(24) Liang, W.; Ma, H.; Zang, H.; Ye, C. Generalized time-dependent approaches to vibrationally resolved electronic and Raman spectra: Theory and applications. *International Journal of Quantum Chemistry* **2015**, *115*, 550–563.

(25) Epifanovsky, E.; Gilbert, A. T.; Feng, X.; Lee, J.; Mao, Y.; Mardirossian, N.; Pokhilko, P.; White, A. F.; Coons, M. P.; Dempwolff, A. L., et al. Software for the frontiers of quantum chemistry: An overview of developments in the Q-Chem 5 package. *The Journal of chemical physics* **2021**, *155*, 084801.

(26) Kasha, M. Characterization of Electronic Transitions in Complex Molecules. *Disc. Faraday Soc.* **1950**, *9*, 14–19.

(27) Vendrell, O. Collective Jahn-Teller Interactions through Light-Matter Coupling in a Cavity. *Phys. Rev. Lett.* **2018**, *121*, 253001.

(28) Ulusoy, I. S.; Gomez, J. A.; Vendrell, O. Modifying the Nonradiative Decay Dynamics through Conical Intersections via Collective Coupling to a Cavity Mode. *J. Phys. Chem. A* **2019**, *123*, 8832–8844.
