# Supplementary material for: Identifying Vibrations that Control Non-adiabatic Relaxation of Polaritons in Strongly Coupled Molecule–Cavity Systems
Source: J Phys Chem Lett. 2022 Jun 30;13(27):6259–67. doi: 10.1021/acs.jpclett.2c00826 (PMC9289944; doi:10.1021/acs.jpclett.2c00826)
Supplement: Supplementary file 2 — jz2c00826_si_002.pdf [file jz2c00826_si_002.pdf]

jz-2022-00826g.R1

Name: Peer Review Information for "Identifying Vibrations that Control Non-adiabatic Relaxation of Polaritons In Strongly Coupled Molecule-Cavity Systems"

First Round of Reviewer Comments

Reviewer: 1

Comments to the Author

The paper analyzes the vibronic coupling mechanism between bright and dark polaritonic states in ensembles of photo-excited tetracene (Tc) molecules in single- and multi-mode cavities. The simulations and theoretical analyses are based on a mixed quantum-classical approach where the atoms are described by their Cartesian positions as classical particles, whereas the electronic and photonic subsystems are considered within the Tavis- Cummings (TC) Hamiltonian.

The paper can be conceptually divided in two parts: (1) first, it characterizes the so-called DS_0 state, the dark polariton reached when one of the Tc molecules relaxes to the minimum of the first singlet excited state S_1 PES and the excitation localizes. (2) Second, it characterizes what kinds of molecular displacements are responsible for the population transfer from DS_0 to the lower bright polaritonic state LP.

The main conclusions of the paper can be summarized as follows:

1. Raman-type (fully symmetric) displacements are responsible for the population transfer. These overlap strongly with the non-adiabatic coupling (NAC) vector between the adiabatic DS_0 and LP states.

2. It is not necessarily required that a vibrational quantum be exchanged to couple DS_0 and LP. The same Raman modes drive the population transfer in vibrationally assisted scattering (VAS) and radiative pumping (RP).

3. The NAC vector scales as $1/\sqrt{N}$ for constant Rabi splitting.

The paper uses an impressive theoretical machinery based on on-the-fly molecular dynamics of many molecules run concurrently and coupled via the TC Hamiltonian. The insights it provides are relevant towards under- standing how complex organic molecules relax and interact in cavities. The use of a multi-mode cavity brings the simulations closer to actual experiments, and, interestingly, the results obtained with it seem to validate that it behaves similarly to single-mode cavities often used in simulations.

I find the connection of polaritonic couplings with Raman spectra an important message for the community, and the fact that VAS and RP appear to follow from the same mechanism is also a valuable insight.

My impression of the paper is, however, a bit mixed. I greatly appreciate the technology and its potential to deliver insights into specific systems and specific experiments. On the other hand, my impression is that the authors used an ax for something that could have been done with a scalpel, to put it graphically. The paper is rather long for a letter and some conclusions are, in my view, not very surprising and not completely new. I think, if some of these edges are polished and the content more tightly packed, it can still make for a good JPCL letter.

My “concerns” are detailed in the following:

A) Relaxation through symmetric modes: Conclusion 1 is not entirely surprising. Full quantum simulations of diatomic [1] and polyatomic molecules [2] in a cavity (also by others, e.g. Mukamel/Kowalewski) have shown how the modulation of the energy-gap difference constitutes the main coupling mechanism between bright and dark polaritonic states, and in dark states among themselves. Indeed, I am surprised at the fact that works based on a full quantum treatment and otherwise quite similar in the rest of the cavity-modeling assumptions (no molecule-molecule interactions, e.g.) were forgotten. Hamiltonian (1), or by the same token Hamiltonian (4) in [1], are of arrowhead form when written in the *diabatic* basis of Eq. (3). In the Condon approximation, the only modulation of their matrix elements, and therefore the driver of population transfer, is the modulation of the energy gap of individual molecules. For diatomics, the only vibrational mode is fully symmetric, for polyatomics one sees that the fully symmetric *tuning* modes are those that drive the energy gap and cause the fast transition among polaritonic states [2]. Same as in this manuscript. The other modes do so only in second order and are less important at short times.

For a more quantitative analysis, one can rotate the basis in Eq. (3) to symmetry-adapted states and show that the vibronic coupling between bright and dark polaritonic states is proportional to the gradient of the energy gap, as seen e.g. in Eqs. S17-S21 of [1].

The authors rightly point to the fact that fully symmetric modes are Raman active, and that therefore Raman spectroscopy can be used to identify modes active in mixing polaritonic states.

B) Reaching the localized DS_0 state: The dynamics studied for a couple of fs in Figure 2 are precisely the kind of relaxation process seen in the time domain in [1,2], and which lead to a localized molecular excitation after the selected molecule leaves the FC point. The only difference is that, here, the authors start from an already localized excitation instead of starting from LP, UP or some combination thereof. Indeed, in Figure 2, an interesting case to examine would be N = 1 *in the cavity*. In this case, the Rabi cycling will be fast enough to affect the S_1 population of molecule 1, and some difference to the bare molecule case might still show up. Presumably, the mean-field nature of Ehrenfest will result in some difficulties in this case, as the molecule will see an oscillatory mean-field between S_0 and S_1. Starting from LP for N > 1 might be even worse, as the Ehrenfest trajectory may not be able to spontaneously break the symmetry of the excitation. Full quantum treatments do not have this problem. Once 16 Tc do the same as 1 Tc *outside* the cavity, there is not much point in keep going, as the excited molecule becomes more and more decoupled from the cavity for 32, 64, etc..

My whole point here is that one could have started directly from the specific arrangement of molecule 1 on the S_1 minimum and all (N − 1) molecules at the FC points. This is where the system will relax with certainty after starting from LP (and UP if given slightly more time. Enough full quantum simulations have shown it. The discussion around Figure 2 seems therefore unnecessary.

C) Analysis in terms of the NAC: There a couple of thoughts here. Equation 23 of the SI shows that the NAC points toward the direction of the gap gradient (Only S_0 appears because S_1 is at its minimum for molecule 1) and in the direction of change of the transition dipole times polarization. As already mentioned, these are the only directions along which vibrations modulate the matrix elements in the *diabatic* basis and therefore induce coupling, so going through the adiabatic basis seems unnecessary. Using an adiababtic picture for polaritonic problems is not exempt of difficulties, as the authors surely know well. For one, once the gap closes, as in the gap between DS_0 and LP, the NAC diverges. The adiabatic representation may (or may not) be convenient for some kinds of dynamical approaches, but I am not convinced that it is the most insightful choice to explain the coupling mechanism among polaritonic states. The representation based on field-free molecular states and cavity states, what I have been calling *diabatic*, delivers the same insights more transparently.

Finally, the fact that the NAC scales as $1/\sqrt{N}$ at constant Rabi cycling appears to be a natural consequence of the fact that the dipolar polaritonic couplings, the off-diagonal elements in Hamiltonian (1), are scaled accordingly. Once Hamiltonian (1) is diagonalized, the NAC follows formally from the gradient of the diabatic- adiabatic transformation matrix, so, it is maybe not surprising that this scaling carries over to the NAC.

D) VAS vs RP:

What is the difference between VAS and RP in terms of the involved matrix elements of the TC Hamiltonian? This is maybe not mentioned very explicitly in the paper. The only difference seems to be related to whether specific vibrational quanta are exchanged during the interaction. Is it so? Could the authors please shed some more light on the this?

Along this line, I am confused by the statement at the end of page 14 that the rate of transfer would be highest when the gap between DS_0 and LP is zero. In this case the NAC diverges and the adiabatic picture definitely breaks down, so it cannot be used to draw conclusions. At constant (zero, e.g.) gap between two *diabatic* states there is population transfer because of the off-diagonal dipolar coupling with the cavity. If the nuclei move, the modulation of the gap in time enters like in the simple Landau-Zenner model [3]. Again, an adiabatic view in the event of close state encounters seems more problematic than helpful.

Minor points:

a) What are “atomically precise” models at the end of page 4? The fact that they are atomistic does not immediately mean that they are accurate: mixed quantum-classical dynamics, not sufficiently accurate electronic structure and insufficient statistical sampling are all drawbacks that these models have. I would suggest to briefly acknowledge them together with the advantages of atomistic simulations, which certainly exist.

b) Around page 15 I would suggest to sub-label *k* as k_z as to remind the reader that this is the in-plane component of the wave vector.

References:

[1]: [10.1103/PhysRevLett.121.253001]

[2]: [10.1021/acs.jpca.9b07404]

[3]: [10.1098/rspa.1932.0165]

Reviewer: 2

Comments to the Author

Summary:

The authors study the main two mechanisms for population transfer of the lower polariton from the dark states of a polaritonic system: vibrational assisted scattering (VAS) and radiative pumping (RP). Such mechanisms, central for applications such as modification of chemical reactions and polariton lasing and condensates, remain still poorly understood. Authors employ computational calculations using nonadiabatic dynamic methods to semiclassically simulate the dynamics of tetracene molecules collectively coupled to an optical microcavity. Authors identified both VAS and RP as non-adiabatic transitions between polariton and dark states, driven by specific vibrational modes. These results can potentially be used to rationally enhance these mechanisms by adjusting the detuning of the cavity to the frequency of such vibrational modes.

Overall, the manuscript is well written and provides significant new insights to the understanding of non- radiative energy transfer in polaritonic systems. After addressing the comments below, we believe this work is suitable for publication on JPCL

Questions and comments:

- In Eq. 1, the Hamiltonian acknowledges position dependent couplings. Are the molecules assumed to be on a regular grid so that H block-diagonalizes into Bloch Hamiltonians H(k)? Or is there positional disorder? Also, is there orientational disorder?

- The authors refer to non-adiabatic couplings causing the energy transfer from the lowest energy dark state into the lower polariton state. Does it mean such states are defined adiabatically as you change the position of the nuclei? Is the basis used in Eq. 3 nuclear-position dependent or is it evaluated at the Franck-Condon point?

- In page 3 authors write: “The eigenstates of the multi-mode Tavis-Cummings Hamiltonian are polaritons”. We think this statement is incorrect since it suggests Dark-States are polaritons despite not having any photonic component.

- Eigenfunctions in equation 3, which are the eigenstates of Hamiltonian in Eq. 1, seem to have no vibrational degrees of freedom (unlike Tavis-Cummings-Holstein model for example). We think the authors should clarify how they identify that the lowest energy dark state corresponds to molecule 1 being *excited in the vibrational ground state of S1*, while the rest remain on the corresponding ground states of S0.

- It would be helpful if the authors clarified whether what they call the dark states have any (even if small) photonic component.

- Also regarding wavefunctions on Eq. 3, why would the authors have a different coefficient for different molecules? Is this just because of nuclear fluctuations and positional and orientational

disorder?

- Functions \psi^{m} on Eq. 3 should be put on a ket.

- Are the polariton states defined by fixing the vibrations to the Franck-Condon region and then multiplied by the vibrational wavepackets? Or instead, they are defined adiabatically as the vibrational modes evolve?

- Throughout the document authors mention “bright polaritonic states”, isn’t it redundant to use the word “bright” since polaritonic already implies they are bright?

- In Figure 2, it is not clear to us what is the actual quantities the authors calculate to get the blue line. We would expect the blue line to be under the black line since the rest of the molecules are in their ground state.

- In page 8 authors write “For quantitative comparisons between the different ensemble sizes, we assigned the same initial velocities to the separate molecules in each of the ensembles by setting the seed of the random number generator that determines what velocities are selected for each atom in the molecule, equal to the index of that molecule.” Is this dynamical disorder fundamentally different to inhomogeneous broadening?

- Can the authors comment on the reasons why it is sufficient to consider only 1 cavity mode for studying VAS while for RP they used 60?

- In page 6th line 50: authors should refer the reader to Figure 1b so it is easier to understand why the lower polariton can be higher in energy than the lowest energy dark state.

- Can the authors elaborate why is there a clear correlation between the Raman spectrum resonances and the vibrational modes responsible for RP and VAS?

- Here’s an important semantic issue that we believe needs to be clarified in the paper. The authors claim that RP and VAS are mediated by nuclear motion. The operational definition the authors have endorsed is that VAS involves relaxation from a dark state that is higher in energy by a discrete number of quanta wrt the LP (page 11), while RP involves a Stokes-shifted dark-state that is resonant with the LP (page 15). However, this operational definition is confusing based on the more standard definition in the literature, where RP is due to dipole-emission from the Stokes-shifted dark-state into the photon component of the LP (in fact, this “standard definition” is also discussed in page 5); after all, RP is “radiative”, involving light. In our opinion, the less confusing claim that the authors could put forward is to say that what the literature identifies as RP IS NOT an RP mechanism whatsoever, but rather a form of VAS.

- However, the most important caveat we want to put forward is the following: It is clear that because of the 1/sqrt(N) scalings of the NACs going from dark to LP states, nuclear dynamics mediated relaxation will die in the thermodynamic limit, which is putatively what matters in microcavities, where N is much larger than 200 (presumably, N>>10^6) [similar scalings, but in the formalism of master equations have been discussed in **New J. Phys. 2015, 17, 053040; Sci. Adv. 5, 12, eaax4482 (2019); J. Chem. Phys. 151, 054106 (2019)]**. The reason why the authors don’t see the standard RP process occurring in their simulation is because nuclei-mediated processes are happening at much shorter timescale than the radiative one (~ns), but this is an artifact of N~200. Thus, we believe that the authors should take a

much more conservative stand in their claims in the abstract as well as in the main text, and restrict their conclusions to small N, which could be relevant for plexcitonic systems, for instance. It is certainly not obvious, based on the presented simulations, that the conclusion that RP and VAS processes (using the authors' operational definition) are triggered by nuclear motion still hold for N>>10^6.

Nonscientific comments:

- Page 6th line 29: I think it would sound better without the coma after "although in experiment"
- Page 15th line 43 typo: replace "tough" by "though"
- Page 10th line 40: remove comma before "increases with N"

Author's Response to Peer Review Comments:

Our response is included in the attached pdf file.

Dear Prof. Editor,

We thank both reviewers and you for the time and effort invested in the evaluation of our manuscript “Identifying Vibrations that Control Non-Adiabatic Relaxation of Polaritons in Strongly Coupled Molecule-Cavity Systems”. We were very pleased that both reviewers considered our work of high interest to the community and hence suitable for *The Journal of Physical Chemistry Letters.* The reviewers also raised several interesting questions and made important suggestions to improve our manuscript.

Reviewer one considered the manuscript too long for a letter, and based on the further comments by this reviewer, we have reduced the length by moving a part that this reviewer considered less surprising, into the Supporting Information. In particular, we now discuss the localization of the excitation onto a single molecule in the lowest-energy dark state after non-resonant excitation, in the Supporting Information. Although both reviewers indicated that the scaling of the relaxation rate had been addressed before, we decided to keep that discussion in the main manuscript, because previous works had approached the scaling from a phenomenological point of view, whereas we provide an explanation for the scaling of the rate with the number of molecules in terms of the non-adiabatic coupling vector, a well-defined quantity that can be computed from first principles with quantum chemistry software. We think this is an important message that we wish to share with the physical chemistry community.

Based on a comment of reviewer one, we furthermore performed additional simulations, which is why it took us more than two weeks to complete our revision. These simulations were motivated by the reviewer’s comment that we could have anticipated that the Raman spectrum provides a good prediction for modes that can drive relaxation under strong light-matter coupling, because for tetracene these modes have to be fully-symmetric and are hence also Raman-active. Thus, we could have predicted most (but not all, because also modes of other symmetries can be Raman-active) of these modes from the Raman spectrum, rather than computing their overlap with the non-adiabatic coupling vector. However, for molecules that lack internal symmetry (C_1), it is not only impossible to identify fully symmetric modes, but also all modes can in principle be Raman active. Therefore, for such molecules, the modes driving relaxation can only be identified by computing their overlap with the non-adiabatic coupling vector. The new simulations of 16 rhodamine molecules, which lack internal symmetry, strongly coupled to a confined light mode, confirm this and hence reinforce our main conclusions. We have added these new results to the manuscript.

We have also addressed all other concerns, answered all questions and revised our manuscript accordingly. On the following pages, you will find our detailed responses to the comments of both reviewers. We hope that these changes make our revision suitable for publication in the *Journal of Physical Chemistry Letters*.

On behalf of all authors,

Ruth Tichauer, Dmitry Morozov and Gerrit Groenhof

PS: Because we could not deduce which reviewer wrote what pdf file, we may have mixed them up in our response. We therefore kindly ask you to send our response to both reviewers to avoid confusion.

Responses to reviewer 1

Comment 1:The paper analyzes the vibronic coupling mechanism between bright and dark polaritonic states in ensembles of photo-excited tetracene (Tc) molecules in single- and multi-mode cavities. The simulations and theoretical analyses are based on a mixed quantum-classical approach where the atoms are described by their Cartesian positions as classical particles, whereas the electronic and photonic subsystems are considered within the Tavis- Cummings (TC) Hamiltonian.

The paper can be conceptually divided in two parts: (1) first, it characterizes the so-called DS_0 state, the dark polariton reached when one of the Tc molecules relaxes to the minimum of the first singlet excited state S_1 PES and the excitation localizes. (2) Second, it characterizes what kinds of molecular displacements are responsible for the population transfer from DS_0 to the lower bright polaritonic state LP.

The main conclusions of the paper can be summarized as follows:

1. Raman-type (fully symmetric) displacements are responsible for the population transfer. These overlap strongly with the non-adiabatic coupling (NAC) vector between the adiabatic DS_0 and LP states.
2. It is not necessarily required that a vibrational quanta be exchanged to couple DS_0 and LP. The same Raman modes drive the population transfer in vibrationally assisted scattering (VAS) and radiative pumping (RP).
3. The NAC vector scales as $1/\sqrt{N}$ for constant Rabi splitting.

Response 1: The reviewer provides a concise summary of our main findings. We agree with this summary and are pleased that all points discussed in our manuscript have been clearly understood by the reviewer.

Comment 2: The paper uses an impressive theoretical machinery based on on-the-fly molecular dynamics of many molecules run concurrently and coupled via the TC Hamiltonian. The insights it provides are relevant towards understanding how complex organic molecules relax and interact in cavities. The use of a multi-mode cavity brings the simulations closer to actual experiments, and, interestingly, the results obtained with it seem to validate that it behaves similarly to single-mode cavities often used in simulations.

I find the connection of polaritonic couplings with Raman spectra an important message for the community, and the fact that VAS and RP appear to follow from the same mechanism is also a valuable insight.

My impression of the paper is, however, a bit mixed. I greatly appreciate the technology and its potential to deliver insights into specific systems and specific experiments. On the other hand, my impression is that the authors used an ax for something that could have been done with a scalpel, to put it graphically. The paper is rather long for a letter and some conclusions are, in my view, not very surprising and not completely new. I think, if some of these edges are polished and the content more tightly packed, it can still make for a good JPCL letter.

Response 2: We are pleased that the reviewer appreciates our methodology and considers our main findings, namely the connection of polaritonic couplings with Raman spectra and the fact that VAS and RP appear to follow from the same mechanism, important. The reviewer however feels that the manuscript is rather long and that some of our results are not surprising or completely new.

We agree that the manuscript is rather long, and followed the suggestion to shorten it by moving a part that the reviewer considers of lower novelty (*i.e.,* the finding that under strong coupling the molecules can still relax into the S_1 minimum) to the Supporting Information (SI).

We discuss the comment of the reviewer about the novelty of our results below in our responses to Comments 3-7.

Comment 3: A) Relaxation through symmetric modes: Conclusion 1 is not entirely surprising. Full quantum simulations of diatomic [1] and polyatomic molecules [2] in a cavity (also by others, e.g. Mukamel/Kowalewski) have shown how the modulation of the energy-gap difference constitutes the main coupling mechanism between bright and dark polaritonic states, and in dark states among themselves. Indeed, I am surprised at the fact that works based on a full quantum treatment and otherwise quite similar in the rest of the cavity-modeling assumptions (no molecule-molecule interactions, e.g.) were forgotten. Hamiltonian (1), or by the same token Hamiltonian (4) in [1], are of arrowhead form when written in the diabatic basis of Eq. (3). In the Condon approximation, the only modulation of their matrix elements, and therefore the driver of population transfer, is the modulation of the energy gap of individual molecules. For diatomics, the only vibrational mode is fully symmetric, for polyatomics one sees that the fully symmetric tuning modes are those that drive the energy gap and cause the fast transition among polaritonic states [2]. Same as in this manuscript. The other modes do so only in second order and are less important at short times.

For a more quantitative analysis, one can rotate the basis in Eq. (3) to symmetry-adapted states and show that the vibronic coupling between bright and dark polaritonic states is proportional to the gradient of the energy gap, as seen e.g. in Eqs. S17-S21 of [1].

The authors rightly point to the fact that fully symmetric modes are Raman active, and that therefore Raman spectroscopy can be used to identify modes active in mixing polaritonic states.

Response 3: The reviewer argues that our finding that relaxation from the dark states into the lower polariton is driven by fully symmetric modes, is not entirely surprising, because similar results have been obtained from full quantum simulations on diatomics (Vendrell, *Phys. Rev. Lett*. 121 (2018) 253001) and polyatomic molecules, represented as a 4-dimensional vibronic coupling model (Ulusoy *et al. J. Phys. Chem. A* 123 (2019) 8832). Despite their relevance for the interpretation of our findings, we had indeed forgotten these papers. We therefore thank the reviewer for bringing these works back to our attention, in particular, because they helped us to better understand why for tetracene the Raman spectrum is such a good predictor of modes that can mediate the relaxation process.

We note, however, that while for molecules with D_{2h} symmetry, such as tetracene, only the fully symmetric modes of A_g symmetry modulate the energy gap, the Raman active modes can be of A_g, B_{1g}, B_{2g}, and B_{3g} symmetry. Indeed, upon closer inspection of the Raman spectrum (Figure 3), we observe for example that the Raman-active mode at 63 meV (mode 15), is of B_{1g} symmetry and does not induce population transfer from DS_0 to LP.

Nevertheless, for molecules that lack internal symmetry (*i.e.,* C_1) the modes that induce transitions between the dark state manifold and the lower polariton cannot be fully symmetric, while in principle all modes can be Raman-active. Therefore, as for tetracene, not all modes that can be identified from the Raman spectrum, will mediate the relaxation into the LP state and *vice versa*. Furthermore, without symmetry, it will be harder to predict what modes will modulate the energy gap, and hence drive the population transfer. In contrast, for such molecules, non-adiabatic coupling vectors can still be computed and used to predict which vibrational modes will drive the population transfer based on their overlap with the non-adiabatic coupling vector. Our approach, therefore, can be used to inform the design of a cavity system for any kind of molecule. To emphasize that

the Raman spectrum is not always a perfect predictor for modes driving relaxation, whereas the overlap with the non-adiabatic coupling can always be used to identify those modes, we have repeated our analysis for Rhodamine, which has C_1 symmetry.

We have revised the text of our manuscript not only to include a more detailed interpretation of our findings and the new results for the rhodamine cavity system, but also to acknowledge the aforementioned previous works and their relevance for our conclusions.

Page 12: "Because Raman spectra have been used to identify vibrational modes that can mediate relaxation,[26,29,31] we also computed the Raman spectrum. In Figure 3c we compare the Raman intensities of the vibrational modes to the overlap of these modes with the non-adiabatic coupling vector. Because tetracene belongs to the D_{2h} point group, both terms in the non-adiabatic coupling vector are fully symmetric (A_g, Equation 23 in SI).[72,73] Therefore, only vibrations of the same A_g symmetry can induce population transfer. Since fully symmetric vibrational modes are also Raman-active, these vibrations appear in the Raman spectrum of Figure 3c. However, because in tetracene, vibrational modes of B_{1g}, B_{2g} and B_{3g} symmetry are Raman-active as well, the Raman spectrum also contains peaks due to vibrations that cannot induce population transfer (*e.g.*, vibrational mode 15 of B_{1g} symmetry at 63 meV), in line with experiment.[29,31]

While for tetracene the modes that drive relaxation, can be identified based on symmetry arguments, for molecules that lack internal symmetry (*i.e.,* C_1), these modes can only be identified by computing their overlap with the non-adiabatic coupling vector. To illustrate this, we have also performed simulations of a single-mode cavity with 16 rhodamine molecules. The results of these simulations, discussed in SI and shown in Figure S8, suggest that indeed the Raman spectrum does not predict all of the modes that can drive the relaxation between the DS_0 and the LP state, whereas these modes are easily identified from their overlap with the non-adiabatic coupling vector."

In addition, we have added a new section to the Supporting Information to show and discuss the results for the Rhodamine system.

Page 21 in SI: "**Relaxation in a strongly coupled Rhodamine-cavity system**

In Figure S8a we plot the overlap between the non-adiabatic coupling vector connecting the DS_0 state and LP on the one hand, and the vibrational modes of Rhodamine on the other hand, as a function of the vibrational energy. To understand which of the vibrational modes can induce population transfer between the DS_0 and LP, we performed 105 short MD simulations of a cavity with 16 Rh molecules. These simulations were initiated in the lowest energy dark state, DS_0, in which one Rh molecule is in the S_1 minimum geometry, while the other 15 molecules are in the S_0 geometry. In each simulation, we selectively activated one of the 105 vibrational modes of the molecule in the S_1 minimum energy geometry by providing as initial atomic velocities the mass-unweighted normal mode vector, which was obtained within the harmonic approximation by diagonalizing the molecular Hessian. This vector was multiplied by a factor 5, such that the initial temperature was around 300 K.

To quantify the extent of population transfer from the DS_0 state into the more photonic LP state during the simulation, we projected the excitation of the single cavity mode (*i.e.*, $\langle 1|\langle S_0^1 S_0^2 .. S_0^{N-1} S_0^{N1}|$, Equation 11) onto the total time-dependent polaritonic wave function ($|\Psi(\mathrm{t})\rangle$, Equation 12). The top panel in Figure S8b shows this photonic weight (*i.e.*, $\left|\sum_m \sum_j c_m \alpha_j^m\right|^2$) at 1 fs as a function of the vibrational energy of the mode along which the initial velocities were directed. The observation that population transfer predominantly occurs if we activate vibrational modes that overlap with the non-adiabatic coupling vector, suggests that also for Rhodamine, which lacks internal symmetry, the relaxation from the dark state manifold into the LP is selectively mediated by these vibrations.

As for Tetracene, we also computed the IR, Raman and vibronic spectra (Figure S8). In contrast to Tetracene, not all modes that can drive population transfer are Raman-active. Therefore, for Rhodamine, which lacks internal symmetry, the Raman spectrum is less useful for predicting such modes. Instead, these modes can only be identified from their overlap with the non-adiabatic coupling vector."

Comment 4: B) Reaching the localized DS_0 state: The dynamics studied for a couple of fs in Figure 2 are precisely the kind of relaxation process seen in the time domain in [1,2], and which lead to a localized molecular excitation after the selected molecule leaves the FC point. The only difference is that, here, the authors start from an already localized excitation instead of starting from LP, UP or some combination thereof. Indeed, in Figure 2, an interesting case to examine would be N = 1 in the cavity. In this case, the Rabi cycling will be fast enough to

affect the S1 population of molecule 1, and some difference to the bare molecule case might still show up. Presumably, the mean-field nature of Ehrenfest will result in some difficulties in this case, as the molecule will see an oscillatory mean-field between S0 and S1. Starting from LP for $N > 1$ might be even worse, as the Ehrenfest trajectory may not be able to spontaneously break the symmetry of the excitation. Full quantum treatments do not have this problem. Once 16 Tc do the same as 1 Tc outside the cavity, there is not much point in keep going, as the excited molecule becomes more and more decoupled from the cavity for 32, 64, etc..

My whole point here is that one could have started directly from the specific arrangement of molecule 1 on the S1 minimum and all $(N - 1)$ molecules at the FC points. This is where the system will relax with certainty after starting from LP (and UP if given slightly more time. Enough full quantum simulations have shown it. The discussion around Figure 2 seems therefore unnecessary.

Response 4: The reviewer points out that also in previous simulations, based on a fully quantum mechanical description of (a reduced number of) molecular degrees of freedom, electrons and (single) cavity mode, localization of the excitation onto a single molecule was observed. Because of these previous observations, the reviewer suggests that we could have started our simulations from a configuration of molecules, in which the excitation is localized onto one molecule (*i.e.,* the DS_0 state).

We agree that the relaxation of a strongly-coupled molecule-cavity system into a localized S_1 state on one of the molecules has been observed before by Vendrel and co-workers, who excited both into the lower (LP) and upper polariton (UP) state in fully quantum mechanical simulations of diatomic molecules (Vendrell, *Phys. Rev. Lett*. 121 (2018) 253001; Ulusoy *et al. J. Phys. Chem. A* 123 (2019) 8832), and also by ourselves (Groenhof and Toppari, *J. Phys. Chem. Lett.* 9 (2018) 4848; Luk et al., *J. Chem. Theory Comput.* 13 (2017) 4324) after excitation into the LP state in semi-classical QM/MM MD simulations of over 1000 molecules. We do not agree that after starting from the LP the system will relax with certainty in the DS_0 state, for example if the LP is lower in energy than the DS_0 state (Luk et al. *J. Chem. Theory Comput.* 13 (2017) 4324), but this was not the case in the simulations shown in Figure 2. We therefore decided to move the discussion on the relaxation into the lowest energy dark state (DS_0), including Figure 2 and Table 1 to the Supporting Information.

We do want to point out that the aim of the simulations was to find out how, after non-resonant excitation into a molecular state, as is often employed in experiments, the system relaxes. That the system relaxes into the DS_0 state, as was seen in previous simulations that started in the LP or UP states, may not be a surprise, in particular in light of the aforementioned papers, but was nevertheless relevant to understand how the extent of localization depends on the size of the system (Table S1), which to the best of our knowledge had not been explored before. Because we found that the localization increases with the system size, we could conclude that also in reality the lowest energy dark state must have the (single-photon) excitation fully localized onto a single molecule, as already suggested by Agranovich and co-workers (Arganovich *et al. Phys. Rev. B* 67 (2003) 085311; Litinskaya *et al. J. Lumin*. 110 (2004) 364) Therefore, as also suggested by the reviewer, we had indeed started the rest of the simulations reported in our manuscript from the specific arrangement of molecule 1 in the S_1 minimum and all $(N-1)$ molecules at the Frack-Condon (FC) points.

Page 7: “The simulations were started in the lowest energy dark state DS_0 (*i.e.*, $\left|c_{\mathrm{DS}_0}(0)\right|^2 = 1$ in Equation 4), identified above, in which the excitation is localized onto a single Tc molecule that has relaxed into the S_1 minimum”

To acknowledge that similar findings about the nature and dynamics of the lowest-energy dark state had been reported before, we include references to the aforementioned papers. In addition, we followed the suggestion of the reviewer to also investigate what happens when there are fewer molecules in the cavity and also added these results to the SI (Figure S3). We found that even for a single molecule in the cavity the excited molecule can reach the S_1 minimum energy geometry, but indeed, as the reviewer pointed out, the dynamics only become virtually unchanged from 16 molecules on.

Page 6: “To understand the nature of the dark states, defined here as states with a total contribution of the cavity mode excitations below a numerical threshold (*i.e.*, $\sum_{k_z}^{n_{\max}} \alpha_{k_z}^m < 0.05$, Equation 3), and, in particular, identify the lowest-energy dark state (DS_0), we simulated the dynamics after non-resonant excitation into the S_1 electronic state of one of the *N* molecules (molecule 1). These simulations were performed with *N* = 1, 2, 4, 16, 32, 64 and 128 Tc molecules strongly coupled to a *single* confined light mode with energy $\hbar\omega_{cav} = 3.22$ eV. Here, we use a single rather than multi-mode cavity to control the energy gap between the dark states and the single LP state via the Rabi splitting, defined as

$$\hbar\Omega^{Rabi} = 2\sqrt{N}|\mu^{TDM}|\sqrt{\hbar\omega_{cav}/2\varepsilon_0 V_{cav}}$$

The Rabi splitting was kept constant at ~429 meV for all systems by scaling the mode volume of the cavities, V_{cav}, with the number of molecules, N, at the start of the simulation (Table S1).

The results of these simulations, discussed in SI, suggest that after non-resonant excitation into the S_1 electronic state of the first molecule, this molecule relaxes into the minimum on its S_1 potential energy surface, while the other molecules remain in their S_0 minimum geometries. This finding is in line with results from previous simulations that also suggest that in the strong coupling regime, molecules can still access the S_1 minimum.[71-74]"

Comment 5: C) Analysis in terms of the NAC: There a couple of thoughts here. Equation 23 of the SI shows that the NAC points toward the direction of the gap gradient (Only S_0 appears because S_1 is at its minimum for molecule 1) and in the direction of change of the transition dipole times polarization. As already mentioned, these are the only directions along which vibrations modulate the matrix elements in the diabatic basis and therefore induce coupling, so going through the adiabatic basis seems unnecessary. Using an adiabatic picture for polaritonic problems is not exempt of difficulties, as the authors surely know well. For one, once the gap closes, as in the gap between DS_0 and LP, the NAC diverges. The adiabatic representation may (or may not) be convenient for some kinds of dynamical approaches, but I am not convinced that it is the most insightful choice to explain the coupling mechanism among polaritonic states. The representation based on field-free molecular states and cavity states, what I have been calling diabatic, delivers the same insights more transparently.

Finally, the fact that the NAC scales as $1/\sqrt{N}$ at constant Rabi cycling appears to be a natural consequence of the fact that the dipolar polaritonic couplings, the off-diagonal elements in Hamiltonian (1), are scaled accordingly. Once Hamiltonian (1) is diagonalized, the NAC follows formally from the gradient of the diabatic- adiabatic transformation matrix, so, it is maybe not surprising that this scaling carries over to the NAC.

Response 5: The reviewer considers that using the adiabatic representation was not necessary to reach some of the conclusions of our work, as the diabatic basis of uncoupled molecular states and cavity states would have delivered the same

insights more transparently, in particular the $1/\sqrt{N}$ dependence of the non-adiabatic coupling vector (NAC).

While we do agree that for the analysis of the scaling of the non-adiabatic coupling vector, and the identification of the normal modes that can induce population transfer between the lowest-energy dark state and the lower polariton, for the highly symmetric tetracene molecule, the diabatic basis of uncoupled molecular states and cavity states would have delivered the same insights, perhaps with less efforts, the results of the simulations do not depend on the representation, adiabatic or diabatic. We understand from the comment that the reviewer is not insisting that we redo our simulations in the diabatic basis, but rather that we justify our choice for the adiabatic representation and take away any concerns about the potential problems of this representations at or near surface crossings, where the energy gap between polaritonic states disappears.

Thus, the main concern of the reviewer seems to be associated with the singularity of the non-adiabatic coupling vector when the energy gap closes, as shown for example in Figure S2 in the Supporting Information of Vendrell, *Phys. Rev. Lett*. 121 (2018) 253001. We agree that such singularities can potentially lead to problems in the propagation of the polaritonic wavefunction when there are (trivial) surface crossings, in which two or more (uncoupled) polaritonic states cross. Therefore, we do *not* use the non-adiabatic coupling vector for the propagation, but instead propagate the polaritonic wave function in the local diabatic basis, as suggested by Granucci and co-workers (Granucci *et al. J. Chem. Phys*. 114 (2001) 10608).

We have extensively tested our implementation of the unitary propagator in this local diabatic basis and can confirm that the polaritonic wave function is propagated correctly, even at (trivial) crossings. For example, in simulations of large ensembles with hundreds of *uncoupled* molecules, the excitation remains localized, despite the very many crossing in the large manifold of near degenerate states. In addition, for systems with up to 1024 molecules, in which one molecule was oriented such that its transition dipole moment is perpendicular to the cavity field and hence uncoupled, there is no population transfer from this molecule into other states, until the transition dipole moment starts aligning with the cavity field due to thermal fluctuations. However, we do realize that we may have not emphasized strongly enough that we do not propagate the polaritonic wave function in the adiabatic basis using the non-

adiabatic coupling vector, but rather in the local diabatic basis. To avoid that readers may have the same concerns as the reviewer about the singularities of the non-adiabatic coupling vectors in the dense manifold of (near) degenerate states of a strongly coupled cavity system with many molecules, we have added a sentence to motivate our choice for propagating in the local diabatic basis:

Page 6: "Here, the c_m(t) are the time-dependent expansion coefficients that are integrated along with the classical trajectories, using the unitary propagator in the local diabatic basis, which is inherently stable even in the case of (trivial) crossings between potential energy surfaces.[69]"

Because the results should not depend on the representation, we acknowledge that our analysis of the non-adiabatic coupling vector in the adiabatic basis could have been done in the diabatic basis as well. However, in our view the results would have not been less surprising, as also the analysis in the adiabatic basis is rather straightforward. While the analysis of the NAC vector in either the adiabatic or diabatic basis yields the expected 1/√N dependence, it is based on a number of assumptions concerning the molecular configuration of the lowest-energy dark state and lower polariton. We therefore used semi-classical atomistic MD simulations to further test this dependency. The good agreement between the predictions and the results of the simulations, confirms in our view that despite the approximations, our model can capture polariton relaxation mechanisms in complex high-dimensional systems. We therefore decided to not repeat the analysis in the diabatic basis, nor remove it completely. Instead, we have added a sentence to acknowledge that the $1/\sqrt{N}$ dependence at constant Rabi splitting is a consequence of the scaling of the off-diagonal elements of the Tavis-Cummings Hamiltonian in the diabatic basis of molecular plus cavity states.

Page 9: "Because we keep the Rabi splitting constant by scaling the cavity mode volume with the number of molecules, the non-adiabatic coupling vector is inversely proportional to the square root of the number of molecules strongly coupled to the cavity (see SI for a derivation)."

Comment 6: D) VAS vs RP: What is the difference between VAS and RP in terms of the involved matrix elements of the TC Hamiltonian? This is maybe not mentioned very explicitly in the paper. The only difference seems to be related to whether specific vibrational quanta are exchanged during the interaction. Is it so? Could the authors please shed some more light on this?

Along this line, I am confused by the statement at the end of page 14 that the rate of transfer would be highest when the gap between DS0 and LP is zero. In this case the NAC diverges and the adiabatic picture definitely breaks down, so it cannot be used to draw conclusions. At constant (zero, e.g.) gap between two diabatic states there is population transfer because of the off-diagonal dipolar coupling with the cavity. If the nuclei move, the modulation of the gap in time enters like in the simple Landau-Zenner model [3]. Again, an adiabatic view in the event of close state encounters seems more problematic than helpful.

Response 6: The reviewer would like to know if there is a difference between vibrationally assisted scattering (VAS) and radiative pumping (RP) in terms of the matrix elements of the Tavis-Cumming Hamiltonian. Also, the reviewer is confused by our statement that the rate of transfer is highest when the lowest-energy dark state and the lower polariton states overlap. Finally, the reviewer points out that when there is such overlap, or (near) degeneracy between states, there would be problems if the non-adiabatic coupling vector in the adiabatic representation were used to propagate the polaritonic wavefunction. Because we already addressed the concern related to the singularity of the non-adiabatic coupling vector near or at degeneracies in our response to comment 5, we will only address the first two issues here.

Concerning the TC matrix elements, we do not change the Hamiltonian. We only create an initial setup of the cavity in our simulations, such that the energy of the DS_0 state is close to that of states on the lower polariton branch. It is under such conditions that RP is reported experimentally. Because we see efficient transfer under these conditions only when vibrational modes that overlap with the non-adiabatic coupling vector, are activated, we conclude that the same mechanism and selection rules apply to RP as to VAS. Because the energy gap is (nearly) zero, there is no need to match the energy gap by excitation of a vibrational quantum. We have rewritten the discussion on the difference between VAS and RP in order to avoid confusion.

In addition, we also removed the statement that “the rate at which the non-adiabatic population transfers, would be highest if the energies of the DS_0 and the LP are degenerate”, as we cannot directly compare the relaxation rates between the various simulations in this work, due to the differences in cavity setups (multi-mode versus single mode, different cavity field strengths, detuning, etc.).

Page 14: "To mimic the initial conditions under which RP is speculated to occur, we modeled a molecule-cavity system with 512 Tc molecules in a periodic one-dimensional cavity (Figure 1b),[42,54] in which the DS_0 state at 2.85 eV is nearly degenerate with several states on the LP branch. The cavity was red-detuned with $\hbar\omega_0 = 2.69$ eV at $k = 0\ \mu\text{m}^{-1}$. To increase the number of LP states with energies near the DS_0 state, the dispersion of this cavity, $\omega_{cav}(k_z) = \sqrt{\omega_0^2 + c^2 k_z^2}$, was modeled with 60 discrete modes, i.e., $k = 2\pi n/L_{cav}$ with $L_{cav} = 15\ \mu\text{m}$ the cavity length and $0 \leq n \leq 59$ the mode index."

In addition, we also write the following on

Page 15: "We therefore suggest that the mechanism previously identified as RP,[57,58] does not involve a direct exchange of a photon, but rather should be considered a form of VAS, which does not require a change in the vibrational eigenstates, because there is no gap between the DS_0 and LP states."

Comment 7:) a) What are "atomically precise" models at the end of page 4? The fact that they are atomistic does not immediately mean that they are accurate: mixed quantum-classical dynamics, not sufficiently accurate electronic structure and insufficient statistical sampling are all drawbacks that these models have. I would suggest to briefly acknowledge them together with the advantages of atomistic simulations, which certainly exist.

Response 7: We agree with the reviewer that with "atomically precise" we may unintentionally imply that we consider our simulations precise or highly accurate, whereas what we meant to convey is that we represent all atoms of the molecules. We have removed this expression, and also added a few sentences to discuss the advantages and disadvantages of our approach.

Page 4: "The latter requires relaxation to be predicted from first principles with quantum chemistry methods, which is the purpose of the present work."

In addition, we added the following text to the SI.

Page 4 SI: "The $V_0(\mathbf{R}_j)$ and $V_1(\mathbf{R}_j)$ adiabatic potential energy surface can be modeled with *ab initio*, density functional theory (DFT), or hybrid Quantum Mechanics / Molecular Mechanics (QM/MM) methods. While these methods provide access to both ground state and excited state potential energy surfaces as well as transition dipole moments for complex systems with many molecular

degrees of freedom, the accuracy critically depends on the level of theory in combination with the size of the atomic basis set. High accuracy results therefore require very large computational efforts. In practice, a trade-off is sought between accuracy and computational efficiency, which often render the results of calculations qualitative, rather than quantitative. Furthermore, because the high dimensionality of the potential energy surfaces precludes a quantum mechanical description of the nuclear degrees of freedom, classical mechanics is used instead. Therefore, nuclear vibrations are not quantized and population transfers between the adiabatic states have to be modeled in an *ad hoc* manner with surface hopping,[7] or Ehrenfest dynamics.[8] In addition, the sampling of all relevant configurations may require more computational resources (and time) than is available. Nevertheless, the main advantage of atomistic models is that despite their limited accuracy, large and complex systems can be modeled directly, and provide *qualitative* insights into the effect of the chemical structure on the dynamics and energetics of a process.“

Comment 8: b) Around page 15 I would suggest to sub-label k as k_z to remind the reader that this is the in-plane component of the wave vector.

Response 8: We agree and thank the reviewer for this typographic suggestion. We have added sub-labels to all instances of *k* in the text.

Responses to reviewer 2

Comment 1: The authors study the main two mechanisms for population transfer of the lower polariton from the dark states of a polaritonic system: vibrational assisted scattering (VAS) and radiative pumping (RP). Such mechanisms, central for applications such as modification of chemical reactions and polariton lasing and condensates, remain still poorly understood. Authors employ computational calculations using nonadiabatic dynamic methods to semiclassically simulate the dynamics of tetracene molecules collectively coupled to an optical microcavity. Authors identified both VAS and RP as non-adiabatic transitions between polariton and dark states, driven by specific vibrational modes. These results can potentially be used to rationally enhance these mechanisms by adjusting the detuning of the cavity to the frequency of such vibrational modes.

Overall, the manuscript is well written and provides significant new insights to the understanding of non- radiative energy transfer in polaritonic systems. After addressing the comments below, we believe this work is suitable for publication on JPCL

Response 1: we are pleased that the reviewer considers that our manuscript provides significant new insights and is supportive of publication. We thank the reviewer for the comments and questions, which we address in detail below and which have helped us to improve the manuscript.

Comment 2: In Eq. 1, the Hamiltonian acknowledges position dependent couplings. Are the molecules assumed to be on a regular grid so that H block-diagonalizes into Bloch Hamiltonians H(k)? Or is there positional disorder? Also, is there orientational disorder?

Response 2: The reviewer wants to know if the molecules are regularly spaced in our one-dimensional periodic cavity model. Indeed, the molecules are positioned at equal distances along the z-axis of the cavity, as illustrated in Figure S1 in the Supporting Information. While we considered neither positional, nor orientational disorder in this work, we had found in previous work that positional disorder has no effect on the relaxation process in multi-mode cavities (Tichauer *et al. J. Chem. Phys.* 154 (2021) 104112). We added a sentence in the revision to emphasize that we make these assumptions.

Page 5: “Using Ehrenfest,[68] or mean-field, dynamics, we computed semi-classical MD trajectories of *N* Tc molecules with their geometric centers evenly distributed along the *z*-axis of the cavity (Figure S1) and with their electronic transition dipole moments aligned to the vacuum field of both single-mode and multi-mode optical cavities.”

Comment 3: The authors refer to non-adiabatic couplings causing the energy transfer from the lowest energy dark state into the lower polariton state. Does it mean such states are defined adiabatically as you change the position of the nuclei? Is the basis used in Eq. 3 nuclear-position dependent or is it evaluated at the Franck-Condon point?

Response 3: The reviewer asks how the adiabatic basis is defined with respect to the nuclear positions, which change during the molecular dynamics simulation. The basis functions in which we expand the adiabatic states ψ^m in Equation 3, are indeed position dependent and are hence evaluated at every configuration of the MD simulation. Because we employ the Born-Oppenheimer approximation we compute at every step of the simulation the adiabatic states by diagonalizing the Tavis-Cummings Hamiltonian in the basis of the field-free molecular plus cavity states. Thus, indeed, as the reviewer points out, also the adiabatic states are (parametric) functions of the coordinates of the nuclei.

The non-adiabatic coupling corrects the limitations of the adiabatic Born-Oppenheimer approximation by inducing population transfers between the various adiabatic states due to nuclear displacements. To avoid confusion, we now more explicitly define the basis functions and emphasize that these states are position dependent:

Page 7 of SI: “Because the energies of the electronic ground and excited state, as well as the transition dipole moments of the molecules, are functions of the positions of their atoms, the basis functions in which the adiabatic polaritonic eigenstates are expanded, depend on the coordinates of all atoms in all molecules, and this dependency carries over to the polaritonic states.”

Comment 4: In page 3 authors write: “The eigenstates of the multi-mode Tavis-Cummings Hamiltonian are polaritons”. We think this statement is incorrect since it suggests Dark-States are polaritons despite not having any photonic component.

Response 4: We thank the reviewer for pointing out that without a photonic contribution a state cannot be considered a polariton. We have changed our manuscript accordingly. For example, on page 3:

"The eigenstates of the multi-mode Tavis-Cummings Hamiltonian are coherent superpositions of excitations in the molecules and of excitations in cavity modes"

Comment 5: Eigenfunctions in equation 3, which are the eigenstates of Hamiltonian in Eq. 1, seem to have no vibrational degrees of freedom (unlike Tavis-Cummings-Holstein model for example). We think the authors should clarify how they identify that the lowest energy dark state corresponds to molecule 1 being *excited in the vibrational ground state of S1*, while the rest remain on the corresponding ground states of S0.

Response 5: Because it was not clear how the vibrational degrees of freedom are included in our model, the reviewer wants us to clarify how we identify that the lowest energy dark state corresponds to molecule 1 being excited in the vibrational ground state of S_1, while the rest remain on the corresponding ground states of S_0.

In our model, the nuclei of the molecules are represented by classical point masses and Newton's equations are used to compute classical trajectories of these point charges under the influence of the forces exerted by the hybrid electronic/photonic (polaritonic, or dark state) wave function. These forces are computed on-the-fly by evaluating the expectation value of the gradient of the total polaritonic energy with respect to the positions of the nuclei. All vibrations are thus purely classical and not quantized. This is the key approximation in all classical and semi-classical molecular dynamics approaches.

To identify the lowest energy dark state, we performed simulations with all molecules initially in the energy minimum of the ground state (0 K, no zero-point energy). In these simulations we observe that the molecule that is excited to the S_1 state, rapidly relaxes into the S_1 minimum. Next, we created such a configuration, (*i.e.,* one molecule in the S_1 minimum and all other molecules in the S_0 minimum) and inspected the character of the light-matter hybrid state. We found that with increasing numbers of molecules, the contribution of the field-free state with the excitation on the molecule in the S_1 minimum, becomes unity. We therefore conclude that in large ensembles (> 2000 molecules) the lowest energy dark state consists of an excitation localized on a single molecule that has

relaxed into the lowest energy state on the S_1 potential energy surface, while all other molecules remain in the lowest energy state on their S_0 potential energy surfaces.

Because the relaxation rate into the lower polariton state, from where the excitation can decay radiatively, is inversely proportional to the number of molecules and the number of molecules is very high, we speculate that the system can survive in that lowest energy dark state sufficiently long to reach thermal equilibrium, in which case all molecules will be in their vibrational ground states.

Note that based on the comments of the other reviewer, we have moved the discussion on the relaxation into the DS_0 state to the Supporting information, and kept a minimal discussion in the main text. To clarify why we concluded that in the lowest energy dark state, the excitation is localized on a single molecule, which has relaxed into the lowest energy vibrational state on the S_1 potential energy surface, while the other molecules remain in their lowest-energy vibrational states on the S_0 potential energy surface, we added the following on page 9:

"Nevertheless, because the rate decreases with increasing N,[22,51,78] we speculate that in real Fabry-Pérot cavities with 10^6 molecules inside the mode volume,[51,78] the lifetime of the DS_0 state will be sufficiently long to reach thermal equilibrium, in particular if the energy gap to the LP states is large. We therefore assume that before relaxation from the DS_0 state into the LP occurs, all molecules are in their vibrational ground states."

Comment 6: It would be helpful if the authors clarified whether what they call the dark states have any (even if small) photonic component.

Response 6: We agree that we should clarify better when we consider a state dark or bright. Because during the molecular dynamics simulations, the molecular coordinates evolve in time, the excitation energies and transition dipole moments change accordingly. Therefore, also the contributions of the molecular excitations and of cavity excitations to the eigenstates of the Tavis-Cummings Hamiltonian vary with time. Because even small geometric displacements can mix in a small fraction of photonic components, we introduce a numerical threshold and consider a state "dark" if the total photonic contribution is below 0.05. For example, the lowest energy dark state with 16 molecules in the single-

mode cavity has a phononic contribution of 0.044 and is thus considered dark. We note that the photonic fraction reduces with increasing the number of molecules in the single-mode cavity and for 128 molecules, the photonic contribution to the lowest energy dark state is 0.0068. We now explicitly report our criteria for considering a state dark:

Page 6: "To understand the nature of the dark states, defined here as states with a total contribution of the cavity mode excitations below a numerical threshold (*i.e.*, $\sum_{k_z}^{n_{\max}} \alpha_{k_z}^{m} < 0.05$, Equation 3)"

Comment 7: Also regarding wavefunctions on Eq. 3, why would the authors have a different coefficient for different molecules? Is this just because of nuclear fluctuations and positional and orientational disorder?

Response 7: The reviewer asks why the coefficients of the field-free molecular plus cavity states are all different and correctly infers that this is due to (thermal) fluctuations in the nuclear positions, which also can lead to orientational disorder. Note that in many of our initial conditions, the coefficients corresponding to excitation of molecules that are in the same configuration (*i.e.,* S_0 minimum energy geometry), are identical. However, during the dynamics, the geometries of the molecules change independently, and hence also their excitation energies and transition dipole moments. Therefore, after some time, the coefficients are all different. To avoid that this important aspect of our model is missed, we have added text.

Page 4: "Due to thermal fluctuations, the excitation energies and transition dipole moments of the molecules span a distribution rather than a single value. Therefore, at thermal equilibrium, the expansion coefficients are all different."

Comment 8: Functions \psi^{m} on Eq. 3 should be put on a ket.

Response 8: We agree and have put the functions in kets everywhere in the text.

Comment 9: Are the polariton states defined by fixing the vibrations to the Franck-Condon region and then
multiplied by the vibrational wavepackets? Or instead, they are defined adiabatically as the vibrational modes evolve?

Response 9: The polariton states (as well as dark states, see comment 3) are defined adiabatically and hence depend parametrically on the nuclear

coordinates. Thus, when the vibrational modes (or in general the nuclear degrees of freedom) evolve, the polaritonic (and dark) states change along.

We have added a statement to clarify this dependency.

Page 6: "which was expanded in the basis of the time-independent eigenfunctions of the Tavis-Cummings Hamiltonian (Equation 3) that depend only on the positions of the atoms in the molecules:[52,54]"

Comment 10: Throughout the document authors mention "bright polaritonic states", isn't it redundant to use the word "bright" since polaritonic already implies they are bright?

Response 10: Since we followed the reviewer's suggestion to distinguish between polariton and dark states based on the contribution of the cavity modes to the eigenfunctions of the Tavis-Cummings Hamiltonian, we also agree to remove the "bright" statements, as these are now redundant.

Comment 11: In Figure 2, it is not clear to us what is the actual quantities the authors calculate to get the blue line. We would expect the blue line to be under the black line since the rest of the molecules are in their ground state.

Response 11: We thank the reviewer for alerting us to the lack of clarity in this figure. Based on a comment by the other reviewer we moved the discussion on the relaxation into the lowest-energy dark state, including Figure 2 and Table 1, into the Supporting Information. In addition, based on what the reviewer points out here, we decided to plot both ground and excited state potential energies for each of the molecules and for the various ensemble sizes. The ground and excited state potential energy change significantly for the molecule that was excited, indicating a geometrical relaxation into the minimum of the excited state potential energy surface. Because the coordinates of the atoms in the other molecules hardly change on the short simulation timescale, the ground and excited state potential energies of these molecules remain highly similar. These profiles thus overlap and cannot be distinguished in the graphs. We explain this in the caption of Figure S3.

Page 13, SI: "

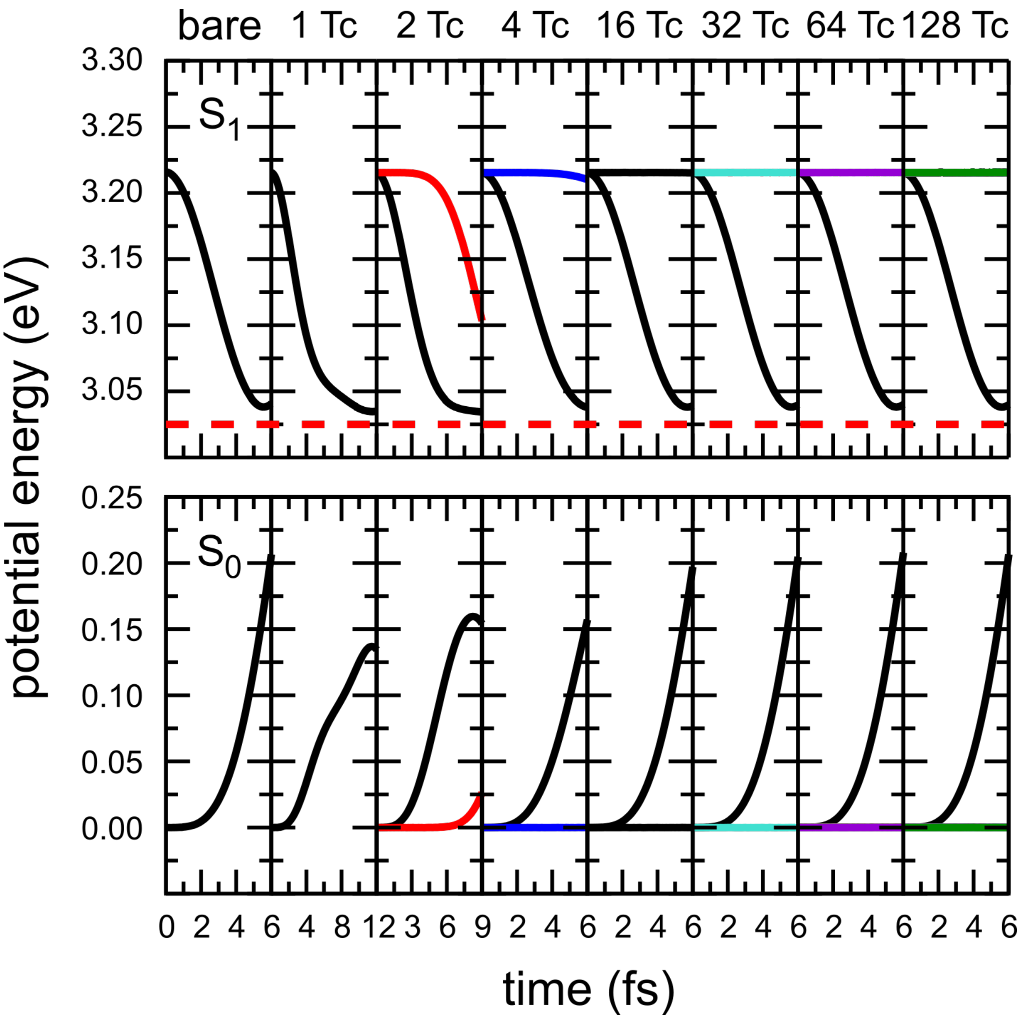

Figure S3: Evolution of the ground (S_0) and excited state (S_1) potential energy of a bare Tc molecule (*i.e.*, outside of the cavity), and of 16, 32, 64 and 128 Tc molecules strongly coupled to a single-mode cavity resonant with the S_1 excitation energy of Tc. The vacuum field strength was adjusted to have the same Rabi splitting (~429 meV) in all cavity systems at the start of the simulation. The black line corresponds to the potential energy of molecule 1, the potential energies of the other molecules are shown in various colors. Note that because the molecules were all in the same configuration at the start of the simulation, their S_0 and S_1 potential energies remain similar and the curves overlap. The red dotted line indicates the energy value of the S_1 minimum, obtained by geometry optimization of the bare molecule in the S_1 state."

Comment 12: In page 8 authors write "For quantitative comparisons between the different ensemble sizes, we assigned the same initial velocities to the separate molecules in each of the ensembles by setting the seed of the random number

generator that determines what velocities are selected for each atom in the molecule, equal to the index of that molecule." Is this dynamical disorder fundamentally different to inhomogeneous broadening?

Response 12: The reviewer would like to understand if our procedure of assigning initial velocities to the molecules for inducing dynamical disorder is fundamentally different from inhomogeneous broadening. Indeed, by assigning velocities from a Maxwell-Boltzmann distribution to the nuclei, we generate an initial condition that corresponds to the canonical ensemble. Because with such initial velocities each molecule samples a Boltzmann distribution independently, their excitation energies can become inhomogeneously broadened, but longer simulations would be needed to see this effect more clearly. We have added a short statement to the text:

Page 7: "These initial conditions can cause inhomogeneous broadening, but only on longer timescales."

Comment 13: Can the authors comment on the reasons why it is sufficient to consider only 1 cavity mode for studying VAS while for RP they used 60?

Response 13: VAS is speculated to occur if there is an energy gap between the dark state manifold and the lower polariton, for example in cavities with TDBC J-aggregates that have a very narrow absorption linewidth and a negligible Stokes shift. Because in a single-mode cavity we can precisely control that energy gap by tuning the vacuum field in our simulations, we used a single-mode cavity for investigating VAS. RP is speculated to occur if there is overlap between the energies of the lowest-energy dark states (or uncoupled molecular excited states) and states of the LP branch. Because during the simulations, the energies of all states fluctuate, we decided to use a multi-mode cavity, in which there are multiple LP states with energies near the energy of the lowest energy dark state. We have added two statements to the main text to emphasize our motivation for the different cavity systems.

Page 6: "Here, we use a single rather than a multi-mode cavity to control the energy gap between the dark states and the single LP state via the Rabi splitting"

Page14: "To increase the number of LP states with energies near the DS_0 state, the dispersion of this cavity was modeled with 60 discrete modes"

Comment 14: In page 6th line 50: authors should refer the reader to Figure 1b so it is easier to understand why the lower polariton can be higher in energy than the lowest energy dark state.

Response 14: We agree and now refer to Figure 1b as follows:

Page 10: “In the 16 molecule system used above, the LP is higher in energy than the DS_0 (In the cavity of Figure 1, such situation would correspond to LP states with $k_z > 8.4\ \mu m^{-1}$)”

Comment 15: Can the authors elaborate why is there a clear correlation between the Raman spectrum resonances and the vibrational modes responsible for RP and VAS?

Response 15: The reviewer would like us to elaborate on why there is a correlation between the Raman spectrum resonances and vibrational modes responsible for RP and VAS. As also brought up by the other reviewer, for molecules with D_{2h} symmetry, such as tetracene, only fully symmetric modes of A_g symmetry can drive the relaxation, while the Raman active modes can be of A_g, B_{1g}, B_{2g}, or B_{3g} symmetry. Indeed, upon closer inspection of the Raman spectrum, we observe for example that the Raman-active mode at 63 meV (mode 15), is of B_{1g} symmetry and does not induce population transfer from DS0 to LP. Nevertheless, apart from this B_{1g} mode, the other Raman-active modes in Tetracene are the same fully symmetric A_g modes that also overlap with the non-adiabatic coupling vector. We have revised the text to reflect this new insight.

Page 12: “Because Raman spectra have been used to identify vibrational modes that can mediate relaxation,[26,29,31] we also computed the Raman spectrum. In Figure 3c we compare the Raman intensities of the vibrational modes to the overlap of these modes with the non-adiabatic coupling vector. Because tetracene belongs to the D_{2h} point group, both terms in the non-adiabatic coupling vector are fully symmetric (A_g, Equation 23 in SI).[72,73] Therefore, only vibrations of the same A_g symmetry can induce population transfer. Since fully symmetric vibrational modes are also Raman-active, these vibrations appear in the Raman spectrum of Figure 4c. However, because in tetracene, vibrational modes of B_{1g}, B_{2g} and B_{3g} symmetry are Raman-active as well, the Raman spectrum also contains peaks due to vibrations that cannot induce population transfer (*e.g.*, the vibrational mode at 63 meV), in line with experiment.[29,11]

While for tetracene the modes that drive relaxation, can be identified based on symmetry arguments, for molecules that lack internal symmetry (*i.e.,* C_1), these modes can only be identified by computing their overlap with the non-adiabatic coupling vector. To illustrate this, we have also performed simulations of a single-mode cavity with 16 rhodamine molecules. The results of these simulations, discussed in SI and shown in Figure S8, suggest that indeed the Raman spectrum does not predict all of the modes that can drive the relaxation between the DS_0 and the LP state, whereas these modes are easily identified from their overlap with the non-adiabatic coupling vector."

Comment 16: Here's an important semantic issue that we believe needs to be clarified in the paper. The authors claim that RP and VAS are mediated by nuclear motion. The operational definition the authors have endorsed is that VAS involves relaxation from a dark state that is higher in energy by a discrete number of quanta wrt the LP (page 11), while RP involves a Stokes-shifted dark-state that is resonant with the LP (page 15). However, this operational definition is confusing based on the more standard definition in the literature, where RP is due to dipole-emission from the Stokes-shifted dark-state into the photon component of the LP (in fact, this "standard definition" is also discussed in page 5); after all, RP is "radiative", involving light. In our opinion, the less confusing claim that the authors could put forward is to say that what the literature identifies as RP IS NOT an RP mechanism whatsoever, but rather a form of VAS.

Response 16: Because in the literature the Radiative Pumping is defined in terms of the dipole-emission from a molecule into the photon component of the LP, the reviewer suggests that we claim, based on our results, that the RP process proposed in the literature, is a form of VAS. We like this suggestion and therefore have revised the text accordingly.

Page 15: "Thus, even though we do not explicitly account for the emission and re-absorption of photons in our simulations, we still observe relaxation from the DS_0 into the LP branch when these states overlap, as required for RP. We also observe that this process is driven by the same vibrational modes as when there is an energy gap between these states. We therefore suggest that the mechanisms previously identified as RP,[57,58] does not involve a direct exchange of a photon, but rather should be considered a form of VAS, which does not require a change in the vibrational eigenstates, because there is no gap between the DS_0 and LP states."

Comment 17: However, the most important caveat we want to put forward is the following: It is clear that because of the 1/sqrt(N) scalings of the NACs going from dark to LP states, nuclear dynamics mediated relaxation will die in the thermodynamic limit, which is putatively what matters in microcavities, where N is much larger than 200 (presumably, N>>10^6) [similar scalings, but in the formalism of master equations have been discussed in New J. Phys. 2015, 17, 053040; Sci. Adv. 5, 12, eaax4482 (2019); J. Chem. Phys. 151, 054106 (2019)]. The reason why the authors don't see the standard RP process occurring in their simulation is because nuclei-mediated processes are happening at much shorter timescale than the radiative one (~ns), but this is an artifact of N~200. Thus, we believe that the authors should take a much more conservative stand in their claims in the abstract as well as in the main text, and restrict their conclusions to small N, which could be relevant for plexcitonic systems, for instance. It is certainly not obvious, based on the presented simulations, that the conclusion that RP and VAS processes (using the authors' operational definition) are triggered by nuclear motion still hold for N>>10^6.

Response 17:

The reviewer points out that because of the $1/\sqrt{N}$ scaling of the non-adiabatic coupling vector, the nuclear dynamics mediated relaxation will disappear in the thermodynamic limit, where N can be of the order of 10^6, or higher. Our findings indeed suggest that without the non-adiabatic coupling, neither VAS or RP (as a special form of VAS), would occur. However, in contrast to the usual thermodynamic limit, in which $N \to \infty$, the number of molecules that can be strongly coupled, is restricted by the mode volume. Even with 10^6 molecules inside the mode volume, the non-adiabatic coupling vector would only be less than two orders of magnitude smaller than the non-adiabatic coupling vector in our 512-molecule system, and thus in line with the much slower relaxation rates observed in experiments. We therefore do not agree to restrict our conclusions to small systems, but instead added a line to the conclusion to speculate on the implications of our finding for real systems.

Page 15: "In the context of our work, the scaling implies that in the thermodynamic limit with approximately 10^6 molecules inside the mode volume of a Fabry-Pérot cavity,[51,78] the non-adiabatic coupling vectors would approximately be 50 times smaller than in the simulations with 512 molecules, and hence still

be in line with the (sub) picosecond relaxation times measured experimentally.[25,39,82,83]"

Comment 18: Nonscientific comments:

- Page 6th line 29: I think it would sound better without the comma after "although in experiment"
- Page 15th line 43 typo: replace "tough" by "though"

- Page 10th line 40: remove comma before "increases with N"

Response 18: We thank the reviewer for spotting these typos, which are now corrected.

jz-2022-00826g.R2

Name: Peer Review Information for "Identifying Vibrations that Control Non-adiabatic Relaxation of Polaritons In Strongly Coupled Molecule-Cavity Systems"

Second Round of Reviewer Comments

Reviewer: 1

Comments to the Author

The authors have addressed all concerns in great detail. They have substantially shortened the manuscript by bringing the description of the structure of the relaxed DS_0 state to the SI. They have also performed new calculations involving a chromophore without symmetry and in this way clarified the role of the Raman modes in mediating the coupling between dark and bright polaritonic states.

In Response 5, the authors correctly assume that I was not asking them to perform new calculations in the diabatic representation. They justify their approach in a clear manner for the reader.

In Response 3 the authors write: "Furthermore, without symmetry, it will be harder to predict what modes will modulate the energy gap, and hence drive the population transfer. In contrast, for such molecules, non-adiabatic coupling vectors can still be computed and used to predict which vibrational modes will drive the population transfer based on their overlap with the non-adiabatic coupling vector."

I do not agree with this statement. The modulation of the energy gap corresponds directly to $\nabla R(V_1(\vec{R}) - V_0(\vec{R}))$. At the FC point, this gradient is non-zero only for totally symmetric modes. If the chromphore has no symmetry beyond C_1, all modes contribute to it. At the end of Page 10, the authors mention that the direction of the NAC vector determines the modes active in the population transfer. I think, the gradient difference serves the same purpose and is easily understood and calculated. If the authors agree with this statement, I think such a remark might be added, as it connects their work with existing results and helps the reader understand the broader picture.

I leave these final considerations to the discretion of the authors and I do not need to see the manuscript again.

Summarizing, this is a clear, very timely and important paper, and I recommend its publication in JPCL.

Reviewer: 2

Comments to the Author

The authors have successfully answered all our concerns and we endorse the publication of such nice work.

Author's Response to Peer Review Comments:

Dear Professor Editor,

We again want to thank you and the reviewers for evaluating the revision of our manuscript. We are very pleased that both reviewers recommend publication. Reviewer 1 suggests that we include a brief statement in the manuscript to indicate that also the gradient difference vector can be used to predict what modes can drive the relaxation from the dark state manifold into the lower polariton. We agree with this suggestion and have hence added such a statement. A more detailed response to the comments of both Reviewers is included below.

We hope that with the additional statement, our manuscript is acceptable for publication in the *Journal of Physical Chemistry Letters*.

On behalf of all authors,

Ruth Tichauer, Dmitry Morozov and Gerrit Groenhof

Response to Reviewer 1

Comment 1: The authors have addressed all concerns in great detail. They have substantially shortened the manuscript by bringing the description of the structure of the relaxed DS state to the SI. They have also performed new calculations involving a chromophore without symmetry and in this way clarified the role of the Raman modes in mediating the coupling between dark and bright polaritonic states.

Response 1: We are pleased that we could address the concerns of the reviewer. We thank the reviewer once more for helping us improve our manuscript.

Comment 2: In Response 5, the authors correctly assume that I was not asking them to perform new calculations in the diabatic representation. They justify their approach in a clear manner for the reader.

Response 2: We are pleased that we understood the comment correctly.

Comment 3: In Response 3 the authors write: "Furthermore, without symmetry, it will be harder to predict what modes will modulate the energy gap, and hence drive the population transfer. In contrast, for such molecules, non-adiabatic coupling vectors can still be computed and used to predict which vibrational modes will drive the population transfer based on their overlap with the non-adiabatic coupling vector."

I do not agree with this statement. The modulation of the energy gap corresponds directly to $\nabla_R(V_1(\mathbf{R}) - V_0(\mathbf{R}))$. At the FC point, this gradient is non-zero only for totally symmetric modes. If the chromophore has no symmetry beyond C_1, all modes contribute to it. At the end of Page 10, the authors mention that the direction of the NAC vector determines the modes active in the population transfer. I think, the gradient difference serves the same purpose and is easily understood and calculated. If the authors agree with this statement, I think such a remark might be added, as it connects their work with existing results and helps the reader understand the broader picture.

I leave these final considerations to the discretion of the authors and I do not need to see the manuscript again.

Response 3: We agree with the reviewer that the gradient difference vector modulates the energy gap, and that for molecules that lack internal symmetry, all modes in principle contribute to this vector. However, our observations in the Rhodamine-cavity simulations suggest that in practice not all vibrational modes can modulate the gap, but rather only a subset of these modes. Nevertheless, because the non-adiabatic coupling vector (Equation 20 of the Supporting Information, SI) contains two terms, the first of which actually is the gradient difference vector $\nabla_R(V_1(\mathbf{R}) - V_0(\mathbf{R}))$, modes overlapping with the NAC must also overlap with the gradient difference, as shown for tetracene in Figure S6 in SI. To acknowledge that the active modes could therefore not only be identified from their overlap with the complete non-adiabatic coupling

vector but also from their overlap with the gradient difference vector, and connect our work more strongly to previous papers, in particular Vendrell *et al. Phys. Rev. Lett.* 121 (2018) 253001 and Ulusoy *et al. J. Phys. Chem. A* 123 (2019) 8832, we have added the following sentence to our manuscript on page 12:

"The non-adiabatic coupling vector contains two terms (Equation 20, SI), the first of which is the gradient difference vector (*i.e.*, $\nabla_R V_{S_1}(\mathbf{R}) - \nabla_R V_{S_0}(\mathbf{R})$). Therefore, as in previous works,[72,73] the modes that drive the relaxation process could be identified from their overlap with the gradient difference vector as well. Indeed, because the gradient difference vector at the S_1 or S_0 minima, describes the displacement between these minima, all modes that are Frank-Condon active can induce population transfer from the DS_0 state into the LP. These modes can either be computed or identified from the vibronic progression in an absorption or emission spectrum (Figure 3d for Tc, Figure S8d for Rhodamine). In contrast to the Raman and vibronic spectra, the IR spectrum, also shown in Figure 3c, is of limited value for predicting vibrational modes that enhance relaxation into the LP for both Tc and Rhodamine."

Comment 4: Summarizing, this is a clear, very timely and important paper, and I recommend its publication in JPCL.

Response 4: We are very pleased that the reviewer considers our manuscript clear, timely and important and that the reviewer recommends publication of our revision.

Response to Reviewer 2

Comment 1: The authors have successfully answered all our concerns and we endorse the publication of such nice work.

Response 1: We thank the reviewer for evaluating the revision and are pleased we could answer all concerns. We’re pleased also that the reviewer endorses the publication of our manuscript.
